# Effect of resistance exercise dose components for tendinopathy management: a systematic review with meta-analysis

**DOI:** 10.1136/bjsports-2022-105754

**Published:** 2023-05-11

**Authors:** Anastasia Vladimirovna Pavlova, Joanna S C Shim, Rachel Moss, Colin Maclean, David Brandie, Laura Mitchell, Leon Greig, Eva Parkinson, Lyndsay Alexander, Victoria Tzortziou Brown, Dylan Morrissey, Kay Cooper, Paul A Swinton

**Affiliations:** 1 School of Health Sciences, Robert Gordon University, Aberdeen, UK; 2 Library Services, Robert Gordon University, Aberdeen, UK; 3 Physiotherapy, Sportscotland Institute of Sport, Stirling, UK; 4 Physiotherapy, NHS Grampian, Aberdeen, UK; 5 Queen Mary University of London, Wolfson Institute of Population Health, London, UK; 6 Sports and Exercise Medicine, Queen Mary University of London, London, UK; 7 Physiotherapy Department, Barts Health NHS Trust, London, UK

**Keywords:** Tendinopathy, Exercise Therapy, Systematic review

## Abstract

**Objective:**

To investigate potential moderating effects of resistance exercise dose components including intensity, volume and frequency, for the management of common tendinopathies.

**Design:**

Systematic review with meta-analysis and meta-regressions.

**Data sources:**

Including but not limited to: MEDLINE, CINAHL, SPORTDiscus, ClinicalTrials.gov and ISRCTN Registry.

**Eligibility criteria for selecting studies:**

Randomised and non-randomised controlled trials investigating resistance exercise as the dominant treatment class, reporting sufficient information regarding ≥2 components of exercise dose.

**Results:**

A total of 110 studies were included in meta-analyses (148 treatment arms (TAs), 3953 participants), reporting on five tendinopathy locations (rotator cuff: 48 TAs; Achilles: 43 TAs; lateral elbow: 29 TAs; patellar: 24 TAs; gluteal: 4 TAs). Meta-regressions provided consistent evidence of greater pooled mean effect sizes for higher intensity therapies comprising additional external resistance compared with body mass only (large effect size domains: *β*_BodyMass: External_ = 0.50 (95% credible interval (CrI): 0.15 to 0.84; p=0.998); small effect size domains (*β*_BodyMass: External_ = 0.04 (95% CrI: −0.21 to 0.31; p=0.619)) when combined across tendinopathy locations or analysed separately. Greater pooled mean effect sizes were also identified for the lowest frequency (less than daily) compared with mid (daily) and high frequencies (more than once per day) for both effect size domains when combined or analysed separately (p≥0.976). Evidence for associations between training volume and pooled mean effect sizes was minimal and inconsistent.

**Summary/conclusion:**

Resistance exercise dose is poorly reported within tendinopathy management literature. However, this large meta-analysis identified some consistent patterns indicating greater efficacy on average with therapies prescribing higher intensities (through inclusion of additional loads) and lower frequencies, potentially creating stronger stimuli and facilitating adequate recovery.

**What is already known on this topic?**

- Tendinopathy is a prevalent condition in both athletic and non-athletic populations commonly affecting the Achilles, rotator cuff, lateral elbow, patellar and hip tendons.
- Exercise therapy is the main mode of conservative treatment for tendinopathies with a focus on resistance exercise, which is shown to be effective in improving patient outcomes.
- Little is known about the effect of different resistance exercise dose components, including intensity, volume and frequency, on patient improvement.
- Previous systematic reviews and meta-analyses that have attempted to investigate exercise dose in tendinopathy have been limited to small numbers of studies.

**What this study adds?**

- This extensive systematic review with meta-analysis included 91 studies (126 treatment arms), and identified common patterns despite large variations across interventions.
- Interventions involving higher intensity resistance exercise, with the addition of external loads, showed greater efficacy compared with body mass only exercise.
- Greater efficacy was seen with interventions performed less frequently, potentially allowing for adequate recovery, compared with higher frequencies of once or more per day.
- There were no consistent results or patterns identified from analyses of resistance exercise volume.

**How this study might affect research, practice or policy**

- Clinicians prescribing resistance exercise therapy for tendinopathy should consider including higher intensities of resistance exercise (through addition of external loads) and allowing adequate recovery between sessions.
- We urge future research on exercise interventions to make use of reporting guidelines and to include full details of all components of exercise dose (intensity, volume, frequency).

## Introduction

Tendinopathy is a prevalent condition involving degenerative changes within tendons of both children and adults, commonly in the Achilles, rotator cuff, lateral elbow, patellar and hip tendons.^1^ It affects athletic and non-athletic populations^2^ and can manifest in persistent pain,^3 4^ swelling,^1^ loss of function and diminished movement.^5^ Exercise therapy is the mainstay of conservative management and has focused largely on resistance exercise, often eccentric actions,^6^ to encourage load tolerance leading to structural adaptations at the musculotendinous unit and functional restoration.^7 8^ Its effectiveness is likely to be influenced not only by the specific exercises but also the magnitude of the stimulus, quantified by the concept of exercise dose.^9^ At the most basic level in clinical settings, exercise dose comprises three variables: intensity, volume and frequency, with overall exercise dose quantified as the product of all three.^10^ As evidence has accumulated on the potential effectiveness of exercise therapies across a range of populations and tendinopathies, it has been recommended that primary studies and evidence syntheses attempt to better quantify dose-response relationships.^9 11 12^ The potential to quantify dose-response relationships may be most feasible within resistance exercise due to the ability to appropriately quantify dose variables including intensity. Initial attempts to synthesise evidence and identify dose-response relationships for exercise therapy in tendinopathy management have been limited by setting restrictive inclusion criteria. Meyer *et al* 12 only included three studies when investigating the effect of eccentric exercise protocols for Achilles tendinopathy. A follow-up review included eight studies,^13^ although the authors concluded that heterogenous outcomes and methodological limitations meant that data could not be pooled, nor recommendations made regarding dose-response. An alternative strategy is to increase the amount of data available by combining heterogenous sources and exploring the variability in results. Young *et al* 14 increased available data for their meta-analysis to 14 studies by including studies investigating multiple common disorders (Achilles tendinopathy, ankle sprains and plantar heel pain). Several trends were identified, including greater effects with increased frequency and progressive exercise compared with pre-prescribed sets and repetitions.^14^ However, no formal statistical comparisons of exercise dose were made, limiting the conclusions that can be drawn. Given the limited attempts to explore dose-response relationships across the wider exercise therapy and tendinopathy literature, the present systematic review with meta-analysis combined data from studies investigating the effectiveness of resistance exercise across the most prevalent tendinopathies (rotator cuff related shoulder pain (RCRSP), lateral elbow, patellar, gluteal or Achilles). The aim was to investigate potential moderating effects of resistance exercise dose components, including intensity, volume and frequency, through contemporary meta-analysis and meta-regression approaches; allowing us to explore the heterogeneity and assess for general trends regarding dose-response relationships.

## Methods

This review was part of a project funded by the National Institute for Health Research (Health Technology Assessment 129388 Exercise therapy for the treatment of tendinopathies) and adhered to an a priori protocol (PROSPERO 2020 CRD42020168187).

### Inclusion criteria

Inclusion criteria and methods were influenced by the project aims, the results of an initial scoping review^15^ and two subsequent stakeholder workshops (n=13). The first included nine individuals who delivered exercise therapy for tendinopathy and had an academic interest. The second included four women with lived experience. Finally, an online survey (n=26) was conducted to gather the views of a more diverse international sample of purposefully selected clinicians and academics. A completed Preferred Reporting Items for Systematic review and Meta-Analysis checklist for reporting of systematic reviews can be found in online supplemental file 1. The inclusion criteria were framed according to a modified PICOS (participant, intervention, comparator, outcomes, study type) approach which also included context.

10.1136/bjsports-2022-105754.supp1 Supplementary data

#### Participants

This meta-analysis included people of any age or gender with a diagnosis of RCRSP, lateral elbow, patellar, Achilles or gluteal tendinopathy of any severity or duration. Due to difficulty in diagnosing the patho-anatomical cause of shoulder pain^16 17^ the term RCRSP is defined here as pain, impaired movement and function of the shoulder from one or more structures (encompassing subacromial pain/impingement syndrome (SIS), rotator cuff tendinopathy and subacromial bursitis).^17^ We included studies that described participants as having tendinopathy, SIS or RCRSP, thereby acknowledging that participants may have tendinopathy+/−involvement of other structures. Full thickness or large tears were excluded, for all tendinopathies. Trial authors’ diagnoses were accepted where a clearly verifiable group of clinical features was reported including: pathognomonic location of pain; a symptom altering response to applied load and/or stretch, with there being a specific test for most tendinopathies; strategies to rule out differential diagnoses; ultrasound or MRI confirmation of structural change. Typically, a minimum of two clinical features were acceptable, however often more were reported. Data from studies with mixed groups were included where there was clear reporting of the tendinopathic group, or they comprised >90% of the investigated cohort.

#### Intervention

The intervention being assessed was exercise therapy where resistance exercise represented the dominant class (see online supplemental file 2 for definitions). Intervention arms combining exercise with other non-exercise therapies were not included. We included resistance exercise delivered in a range of settings by a range of health and exercise professionals or support workers, as well as supervised or unsupervised (including home) exercise. Studies had to report sufficient information regarding exercise dose, including frequency (number of training sessions performed per week), volume (total number of repetitions) and intensity (limb/bodyweight vs additional external load expressed in absolute or relative terms). Where insufficient information was presented, the publishers’ website was searched for supplementary files. Studies were included if a minimum of two of three dose components could be quantified.

10.1136/bjsports-2022-105754.supp2 Supplementary data

#### Comparator

No head-to-head comparators were included, and analyses were conducted across levels of the dose moderator variables.

#### Outcomes

Based on initial review results^15 18^ and stakeholder workshops we included outcomes that assessed six domains: (1) disability; (2) function; (3) pain (eg, pain on loading, pain over a specified time, pain without further specification); (4) range of motion for RCRSP; (5) physical function capacity; and (6) quality of life. Definitions of each domain and example tools are presented in online supplemental file 3.

10.1136/bjsports-2022-105754.supp3 Supplementary data

#### Types of studies

We included randomised controlled trials and non-randomised controlled trials where at least one intervention arm comprised an exercise therapy where resistance exercise was judged to be the dominant treatment class based on the composition of the therapy.

#### Context

The context included primary care, secondary care or community locations in nations defined as very high or high on the Human Development Index (top 62 countries at the time of protocol development)^19^ for the findings to be relevant to the UK context.

### Search strategy

The search strategy used for this study was part of a larger search conducted to scope the entire exercise for tendinopathy research base. We employed a three-step search strategy. First, a limited search of MEDLINE and CINAHL using initial keywords (MH tendinopathy OR TX tendin* OR TX tendon*) AND (MH exercise OR TX exercis*) was conducted to develop a full search strategy. Second, the full search strategy was adapted to each database and applied systematically to: MEDLINE, CINAHL, AMED, Embase, SPORTDiscus, Cochrane library (Controlled trials, Systematic reviews), JBI Evidence Synthesis, PEDRo and Epistemonikos. The following trial registries were also searched: ClinicalTrials.gov, ISRCTN The Research Registry, EU-CTR (European Union Clinical Trials Registry), ANZCTR (Australia and New Zealand Clinical Trials Registry) (all search strategies are presented in online supplemental file 4). Finally, the third step involved a search of cited and citing articles using Scopus and hand-searching 130 systematic reviews that were identified to include information relevant to exercise therapy and tendinopathy. As a final check, the list of identified studies was sent to experts external to the research team to identify any potentially missing studies. Research studies published in languages other than English were translated via Google Translate or international collaborations of the review team. Searches were initiated from 1998 as (1) the heavy load eccentric calf-training protocol for Achilles tendinosis by Alfredson *et al* 20 was published in 1998 and may be considered seminal work in the field of tendinopathy, and (2) there has been a proliferation of research on exercise interventions for tendinopathies post 1998. The final search was conducted on 25 March 2022.

10.1136/bjsports-2022-105754.supp4 Supplementary data

### Study selection

Proquest Refworks was used to manage references and remove duplicates, before importing to Covidence (Melbourne, Australia) for screening and further de-duplication. Each title and abstract was independently reviewed by any two members of the review team (PAS/KC/LA/RM/LG/EP/JSCS/AVP). Full-texts of included studies were similarly screened independently by any two team members. Conflicts were resolved by discussion or by a third reviewer.

### Data extraction

Following extraction training, data were extracted independently by eight members of the review team (PAS/KC/LA/RM/LG/EP/JSCS/AVP) into prepiloted excel spreadsheets and independently coded as described in the accompanying extraction codebook (online supplemental file 5). Each entry on the spreadsheet was double-checked by a different member of the team. Where pre-post intervention data were not presented in text but in figures, data were extracted using PlotDigitizer V.2.6.8 Windows (WebPlotDigitizer - Copyright 2010–2021 Ankit Rohatgi (automeris.io)).

10.1136/bjsports-2022-105754.supp5 Supplementary data

### Risk of bias assessment

Risk of bias was assessed using the earlier version of the Cochrane risk of bias (RoB) tool^21^ since a recent review of RoB tools in tendinopathy management studies did not identify one tool as being superior to the others.^22^ Furthermore, it allowed us to streamline the process by combining with RobotReviewer,^23^ a machine learning software that semi-automates the Cochrane tool. A risk of bias judgement was made for each outcome and time point within studies for each of the seven domains^21^ and reported as either ‘low risk’, ‘high risk’ or ‘uncertain’ when there was insufficient detail or the outcome was not addressed. RobotReviewer was used to make initial assessments on domains 1, 2 and 3 and validated manually using the extracted free text to agree on a final selection of risk of bias. This semi-automated process provided greater efficiency and consistency during the review process. Results are presented using an overall summary risk of bias assessment, obtained for each domain by selecting the mode risk category across all outcomes and time points. An assessment was made by any two members of the team (AVP, JSCS, RM, EP, LG] with comments made to justify scoring and regular consultation between team members where uncertainties arose.

### Coding of resistance exercise therapies

Attempts were made to code exercise dose components (intensity, volume and frequency) for each study; however, sufficient information was not always available to code all three components. Coding of exercise intensity was initially achieved by identifying whether exercise load was prescribed in absolute (eg, kilogrammes when using dumbbells or isoinertial loads) or relative terms (as a percentage of the maximum load that can be lifted) and the magnitude of the load recorded. Additionally, a binary coding was used to identify whether exercise was performed with body mass only (eg, whole body mass or mass of a limb), or with the addition of external loads (such as a loaded backpack, dumbbell or elastic resistance). Exercise volume was coded by quantifying the number of sets and repetitions. Exercise frequency was recorded as the total number of resistance exercise sessions performed per week (including where there were multiple sessions a day). In cases where several resistance exercises were prescribed, intensity and volume were extracted for the primary resistance exercise only, which we defined as the exercise that was the focus of the paper or, if unclear, whichever had the greatest volume. In cases where exercise dose progressed, we took the average value for the primary exercise. Where progressions led from an initial mobility component to a resistance exercise focus, the latter was extracted.

### Statistical analysis

The purpose of the meta-analysis was to investigate responses to exercise therapies where resistance exercise was the dominant treatment class. A broad modelling perspective was selected where outcomes across a range of tendinopathies and outcome domains were combined to investigate whether central estimates (eg, pooled mean) were associated with different levels of moderator variables representing exercise dose (frequency, intensity or volume). Due to the use of different outcome domains and different tests within the same outcome domain, pooling of data required standardisation. This was achieved using the standardised mean difference (SMD_pre_) effect size, dividing the mean group change by the pre-intervention SD. Where baseline SD values were not presented these were estimated using statistical information presented^24^ (eg, CIs, SEs, t values, p values, F values) or imputed based on the simple linear regression quantifying the relationship between the log-transformed means (explanatory) and log-transformed SDs (response) from studies with complete data.^25^ Where required, SMD_pre_ values were reflected by multiplying by –1 to ensure that positive values represented an improved clinical effect. Where multiple outcomes were reported from the same study (different outcomes and/or the same outcome at multiple time points), all possible SMD_pre_ values were calculated and included in the meta-analysis models. To account for covariances created, all meta-analyses were conducted using a nested four-level model^26^ comprising the individual study (level 4), the outcome (level 3), the measurement occasion (level 2) and the sampling variance (level 1) levels. A comprehensive description of the model and further details of statistical analysis can be found in online supplemental file 6.

10.1136/bjsports-2022-105754.supp6 Supplementary data

### Confidence in cumulative evidence

Assessments were made using the Grading of Recommendations Assessment Development and Evaluation guidelines^27^ in addition to recommendations on transparent reporting of evidence for tendinopathy management.^28^ Confidence in evidence was assessed at the outcome level with: (1) overall risk of bias ranked as high, low or unclear risk, as identified by the mode rating across all data in the specific analysis; (2) inconsistency assessed based on meta-analysis results and comparisons of central and variance parameter estimates (downgraded where $\sigma_{r}$ >0.9 ${\hat{\gamma }}_{0}$ ); (3) imprecision judged by the number of available data points (studies, treatment arms, outcome measures) and the width of credible intervals for central estimates; (4) indirectness identified as low risk for all outcomes based on inclusion criteria from our previous scoping review and stakeholder recommendation; and (5) small study effects assessed by visual inspection of effect size distribution and sampling variance (downgraded when substantive number of points outside bounds). Overall confidence in evidence for each analysis was recorded as either high, moderate, low or very low. Categorisations began with high confidence in cumulative evidence and were downgraded to a level for each domain not judged as low risk.

### Protocol deviations

A deviation from our PROSPERO registered protocol in relation to the included tendinopathies was made. We intended to include all tendinopathies but were guided by identification of studies reporting on resistance exercise following our scoping review. Our final inclusion criteria incorporated RCRSP, lateral elbow, patellar, Achilles and gluteal tendinopathies.

### Equality, diversity and inclusion statement

The authors on this project were chosen on merit and came from a diverse range of backgrounds, occupations and levels of seniority. Although we excluded studies from countries not on the ‘very high’ Human Development Index (HDI) list, this was done to make findings more generalisable to the UK.

## Results

### Study selection

**Figure 1:**
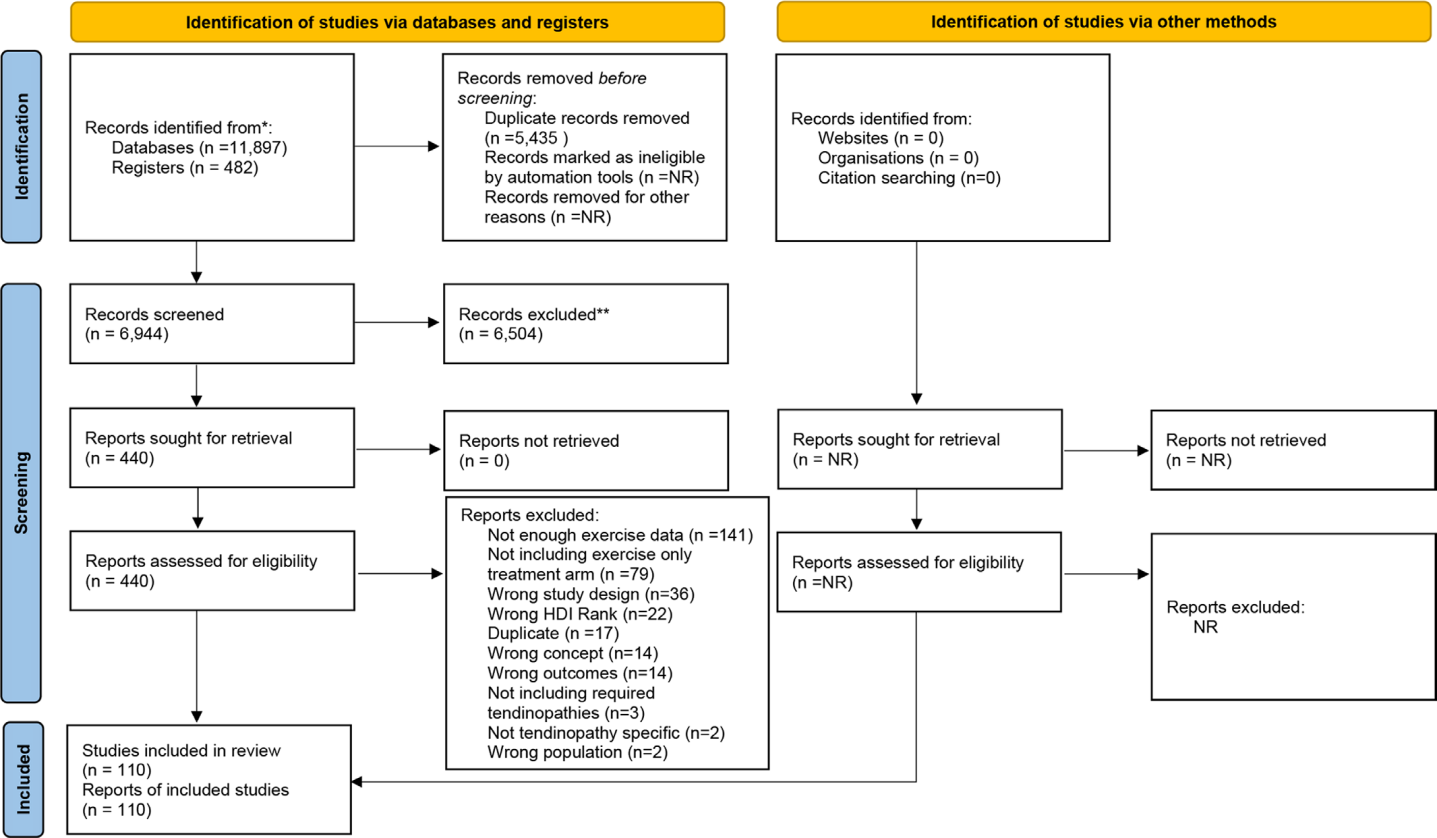
PRISMA flow chart of study selection process. From Page *et al*.^41^ HDI, Human Development Index; PRISMA, Preferred Reporting Items for Systematic review and Meta-Analysis.

The search strategy identified a total of 12 379 potential studies, with 6944 remaining following de-duplication (figure 1). After title and abstract screening 440 studies were retained for full-text screening. Of these studies, a further 330 were excluded (online supplemental file 7A: Excluded studies with reasons reference list) based primarily on insufficient description of the exercise stimulus (141 studies) and not including exercise-only treatment arms (79 studies). In total, data from 110 studies comprising 148 treatment arms and 3953 participants were included in the meta-analyses (online supplemental file 7B, C: Table of included studies and reference list). Exercise therapies for the treatment of five different tendinopathies (RCRSP: 48 (32%) treatment arms; Achilles: 43 (29.0%) treatment arms; lateral elbow: 29 (20%) treatment arms; patellar: 24 (16%) treatment arms; and gluteal: 4 (3%) treatment arms) were identified (table 1). Over half of the treatment arms (82/55%) comprised resistance only therapies, with the remaining predominantly including additional flexibility exercises (table 1). The dominant resistance exercise treatments are presented in table 1, with eccentric only exercise the most common for Achilles (79% of relevant treatment arms), lateral elbow (62% of relevant treatment arms) and patellar (38% of relevant treatment arms) tendinopathies; and both concentric and eccentric resistance exercise most common for gluteal (75% of relevant treatment arms) and RCRSP (73% of relevant treatment arms). Overall, eccentric-only (68 treatment arms) was the most common dominant treatment, followed by concentric and eccentric (55 treatment arms) then isometric (16 treatment arms).

10.1136/bjsports-2022-105754.supp7 Supplementary data

**Table 1:** Dominant resistance exercise treatments presented according to tendinopathy type

Tendinopathy type	Resistance exercise treatment	Number (%) of treatment arms
Achilles	Eccentric only	34 (79)
	Concentric and eccentric	6 (14)
	Concentric only	2 (5)
	Isometric	1 (2)
Gluteal (including greater trochanteric pain syndrome)	Concentric and eccentric	3 (75.0)
	Isokinetic	1 (25.0)
Lateral elbow	Eccentric only	18 (62)
	Isometric	6 (21)
	Concentric and eccentric	3 (10)
	Concentric only	2 (7)
Patellar	Eccentric only	9 (38)
	Concentric and eccentric	8 (33)
	Isometric	5 (21)
	Concentric only	1 (4)
	Isokinetic	1 (4)
Rotator cuff related shoulder pain	Concentric and eccentric	35 (73)
	Eccentric only	7 (15)
	Isometric	4 (8)
	Concentric only	1 (2)
	Isokinetic	1 (2)

### Risk of bias and confidence in cumulative evidence

RoB and confidence in evidence assessments are presented for the primary meta-analyses and more broadly in online supplemental file 8. Summarised according to studies, the most frequent risk of bias for randomised controlled trials was blinding of participants (40% studies high risk of bias) and ‘other bias’ (54% studies high risk of bias). Similarly, ‘other bias’ was also the most frequent risk of bias for non-randomised studies as assessed by the RoB tool. In general, confidence in evidence was frequently low based on imprecision due to wide credible intervals and inconsistency due to large between study variance estimates. Overall confidence in cumulative evidence varied from very low to moderate with low confidence most commonly identified.

10.1136/bjsports-2022-105754.supp8 Supplementary data

### Resistance exercise intensity

Of the 148 treatment arms included, 123 provided sufficient information to categorise the intensity as lower intensity in the form of body mass only (31 treatment arms; 25%), or higher intensity with the addition of external resistance (92 treatment arms; 75%) prescribed based on absolute loads (eg, addition of weights to a backpack, isoinertial loads, resistance band and dumbbells) or percentage of a maximum. Meta-regressions provided consistent evidence of greater pooled mean effect sizes for increased training intensity with the addition of external loads. Primary meta-analyses pooling data across all tendinopathy locations identified median increases of *β*_BodyMass: External_ = 0.50 (95% credible interval (CrI): 0.15 to 0.84; p=0.998) for outcomes generating large effect sizes, and an increase of *β*_BodyMass: External_ = 0.04 (95% CrI: −0.21 to 0.31; p=0.619) for outcomes generating small effect sizes (individual levels presented in table 2). Similarly, point estimates indicated greater pooled mean values for the addition of external resistance for all analyses separated by tendinopathy location for which there was sufficient data (online supplemental file 9A).

10.1136/bjsports-2022-105754.supp9 Supplementary data

**Table 2:** Moderator analysis comparing average pooled effect size for body weight interventions versus interventions including additional external load

Moderator	Pooled SMD_pre_ estimate (95% CrI)	Probability	Study VPC (75% CrI)	Outcome VPC (75% CrI)	Measurement occasion VPC (75% CrI)	Confidence in evidence
Large effect outcomes						
Body mass (169 outcomes 28 treatment arms)	0.9 (0.58 to 1.2)	*p* (body weight < additional) = 0.998	0.78 (0.74 to 0.84)	0.18 (0.14 to 0.23)	0.02 (0.00 to 0.06)	Low
Additional external (544 outcomes 90 treatment arms)	1.4 (1.2 to 1.6)					Moderate
Small effect outcomes						
Body weight (96 outcomes 11 treatment arms)	0.40 (0.21 to 0.53)	*p* (body weight < additional) = 0.619	0.70 (0.63 to 0.77)	0.27 (0.20 to 0.34)	0.02 (0.00 to 0.05)	Low
Additional external (331 outcomes 49 treatment arms)	0.44 (0.33 to 0.55)					Moderate

Results presented across all tendinopathies combined.

Large effect outcomes: Effect sizes obtained from outcomes measuring: (1) Disability; (2) Pain on loading/activity; (3) Pain without further specification; (4) Function; and (5) Pain over a specified time. Small effect outcomes: Effect sizes obtained from outcomes measuring: (1) Quality of Life and (2) Physical functional capacity.

CrI, credible interval; VPC, variance partition coefficient.

### Frequency of resistance exercise

Of the 148 treatment arms included, 135 provided sufficient information to categorise the frequency as low frequency (less than daily: 48 treatment arms; 36%), moderate frequency (daily: 34 treatment arms; 25%) or high frequency (more than once per day: 53 treatment arms; 39%). Consistent evidence of a moderating effect was also identified for resistance exercise frequency with greater pooled mean effect sizes identified for the lowest frequency of less than once per day. Primary meta-analyses pooling data across all tendinopathy locations identified median increases of *β*_<*Daily: Daily*_ = −0.50 (95% CrI: −0.88 to −0.11; p=0.992) between less than once per day and once per day, and *β*_<*Daily: >Daily*_ = −0.44 (95% CrI: −0.829 to −0.05; p=0.951) between less than once per day and more than once per day for outcomes generating large effect sizes. Similarly, median increases of *β*_<*Daily: Daily*_ = −0.32 (95% CrI: −0.55 to −0.09; p=0.999) and *β*_<*Daily: Daily*_ = −0.21 (95% CrI: −0.42 to −0.00; p=0.976) were identified across the comparisons for outcomes generating small effect sizes (individual levels presented in table 3). Consistent evidence of increased pooled mean effect sizes for resistance exercise performed less than once per day was also obtained when analyses were separated by tendinopathy location (online supplemental file 9B). In contrast, effect size estimates tended to be similar for exercising once per day or more than once per day (table 3) with wide overlap of potential values also identified when analyses were separated by tendinopathy location (online supplemental file 9B).

**Table 3:** Moderator analysis comparing average pooled effect size for different training frequencies

Moderator	Pooled SMD_pre_ estimate (95% CrI)	Probability	Study VPC (75% CrI)	Outcome VPC (75% CrI)	Measurement occasion VPC (75% CrI)	Confidence in evidence
Large effect outcomes						
Less than daily (270 outcomes 45 treatment arms)	1.5 (1.3 to 1.7)	*p* (less than daily > once per day) = 0.992	0.77 (0.71 to 0.82)	0.19 (0.15 to 0.24)	0.04 (0.00 to 0.08)	Low
Once per day (192 outcomes 33 treatment arms)	1.0 (0.69 to 1.3)	*p* (once per day < more than once per day) = 0.678				Moderate
More than once per day (305 outcomes 51 treatment arms)	1.2 (1.0 to 1.4)	*p* (less than daily > more than once per day) = 0.951				Moderate
Small effect outcomes						
Less than daily (174 outcomes 25 treatment arms)	0.60 (0.46 to 0.74)	*p* (less than daily > once per day) = 0.999	0.67 (0.58 to 0.74)	0.30 (0.23 to 0.39)	0.02 (0.00 to 0.06)	Moderate
Once per day (156 outcomes 20 treatment arms)	0.28 (0.10 to 0.45)	*p* (once per day < more than once per day) = 0.802				Moderate
More than once per day (107 outcomes 19 treatment arms)	0.39 (0.22 to 0.53)	*p* (less than daily > more than once per day) =0.976				Low

Results presented across all tendinopathies combined.

Large effects: Effect sizes obtained from outcomes measuring: (1) Disability; (2) Pain on loading/activity; (3) Pain without further specification; (4) Function; and (5) Pain over a specified time. Small effects: Effect sizes obtained from outcomes measuring: (1) Quality of Life and (2) Physical functional capacity.

CrI, credible interval; VPC, variance partition coefficient.

### Resistance exercise volume

Resistance exercise volume was categorised for 128 treatment arms as the product of the number of sets and repetitions for the primary resistance exercise. The most common number of total repetitions was 45 (eg, 3 sets of 15 repetitions) and this accounted for almost half of the training interventions (51 treatment arms; 40%). As a result, training volume was coded as a binary variable characterised as lower volume (<45 total repetitions: 67 treatment arms; 52%) and higher volume (≥45 total repetitions: 61 treatment arms; 48%). In general, considerable overlap was identified between pooled mean effect size estimates of lower and higher volume exercise including primary meta-analyses of outcomes generating large effect sizes (*β*_Lower: Higher_ = −0.02 (95% CrI: −0.40 to 0.37; p=0.553)) and outcomes generating small effect sizes (*β*_Lower: Higher_ = −0.14 (95% CrI: −0.35 to 0.09; p=0.782; individual levels presented in table 4)). While the median point estimates from the primary meta-analyses favoured lower volume exercise, this ordering was not consistently maintained when analyses were separated by tendinopathy location (online supplemental file 9C).

**Table 4:** Moderator analysis comparing average pooled effect size for binary resistance volume categorisation

Moderator	Pooled SMD_pre_ estimate (95% CrI)	Probability	Study VPC (75% CrI)	Outcome VPC (75% CrI)	Measurement occasion VPC (75% CrI)	Confidence in evidence
Large effect outcomes						
Lower volume (377 outcomes 63 treatment arms)	1.5 (1.3 to 1.7)	*p* (higher volume < lower vol) = 0.995	0.80 (0.74 to 0.85)	0.17 (0.13 to 0.21)	0.03 (0.00 to 0.07)	Moderate
Higher volume (355 outcomes 60 treatment arms)	1.2 (0.95 to 1.3)					Moderate
Small effect outcomes						
Lower volume (224 outcomes 34 treatment arms)	0.56 (0.37 to 0.74)	*p* (higher volume < lower vol) = 0.782	0.71 (0.63 to 0.78)	0.27 (0.20 to 0.35)	0.02 (0.00 to 0.05)	Moderate
Higher volume (183 outcomes 25 treatment arms)	0.42 (0.26 to 0.59)					Moderate

Results presented across all tendinopathies and individual tendinopathies.

Large effects: Effect sizes obtained from outcomes measuring: (1) Disability; (2) Pain on loading/activity; (3) Pain without further specification; (4) Function; and (5) Pain over a specified time. Small effects: Effect sizes obtained from outcomes measuring: (1) Quality of Life and (2) Physical functional capacity.

CrI, credible interval; VPC, variance partition coefficient.

### Combined analysis

As a final analysis, a meta-regression including the above intensity, frequency and volume variables were included to assess for differences in the pooled mean effect size while controlling for each other across all tendinopathy locations. A total of 76 studies (101 treatment arms) provided sufficient information for simultaneous coding of all three dose variables for outcomes generating large effect sizes, and 40 studies (53 treatment arms) provided sufficient information for outcomes generating small effect sizes. Results were consistent with analyses conducted individually on dosing variables, with evidence of increased pooled means with greater intensity for both outcomes generating large ( $\beta_{BodyMass:External}=$ 0.38 (95% CrI: 0.00 to 0.77; p=0.975)) and small (*β*_BodyMass: External_ = 0.17 (95% CrI: −0.11 to 0.46; p=0.888)) effect sizes. Similarly, evidence of increased pooled means was obtained for the lowest frequency therapies for both outcomes generating large (*β*_<*Daily: Daily*_ = −0.60 (95% CrI: −1.1 to −0.13; p*=*0.993); *β*_<*Daily: >Daily*_ = −0.32 [95% CrI:−0.76 to −0.02; p*=*0.977)) and small (*β*_<*Daily: Daily*_ = −0.37 (95% CrI:−0.63 to −0.08; p*=*0.995); *β*_<*Daily: >Daily*_ = −0.26 (95% CrI:−0.51 to −0.01; p=0.976)) effect sizes. Finally, minimal evidence was obtained for a moderating effect of training volume for outcomes generating either large (*β*_Lower: Higher_ = −0.12 (95% CrI: −0.43 to 0.17; p=0.614)) or small (*β*_Lower: Higher_ = −0.08 (95% CrI: −0.30 to 0.13; p=0.707)) effect sizes.

## Discussion

Our review provides the largest synthesis of training dose in resistance exercise therapy for tendinopathy management to date. We included 110 studies across the five most common tendinopathy locations. Studies included diverse therapies with many comprising resistance exercise only, and others frequently combining resistance exercise with flexibility training. Despite the extensive variability in therapies, some general patterns were identified, indicating that increased loading with greater time for recovery may produce superior results. Meta-regressions consistently identified greater effect size estimates for therapies employing higher intensity exercise through the addition of external loads compared with body mass only. Similarly, meta-regressions consistently identified greater effect size estimates for therapies performed with a low frequency (less than once per day) compared with very high frequencies (once per day or more than once per day) that were also likely to comprise reduced loading to enable recovery. Less consistent results were obtained for moderator analyses investigating exercise volume.

One of the challenges in investigating resistance intensity was the lack of clear reporting of actual intensities used. Studies using resistance bands did not report the relative resistance provided or in general comment on intensity progression. Although some studies identified progression in intensity through additional loading using, for example, a dumbbell or loaded backpack, many did not state the actual loads recommended or used. Due to these limitations a cruder proxy of resistance intensity was investigated in this review based on the binary categorisation of lower intensity exercise involving just body mass, or higher intensity exercise involving additional external resistance. Evidence from our review indicating superior results with greater resistance training intensities is consistent with findings from previous studies that have also reported better adaptive responses in the mechanical properties of tendons.^29 30^

In our review, consistent evidence was obtained indicating that performing resistance exercises less frequently throughout the week (less than once per day) was more effective compared with once per day or greater. To achieve musculotendinous unit hypertrophy with resistance exercise requires high levels of activation.^31 32^ Taking into consideration the microtrauma caused by resistance exercise in the tendon tissue this would be optimised with adequate rest periods between sessions.^31 32^ Allowing greater recovery times between sessions may play a role in the effectiveness of interventions. This is in contrast to the results in a recent review by Young *et al* 14 who reported larger effect sizes with greater frequencies of exercise. However, these differences are likely due to the differences in the evidence-base (14 vs 110 studies) and inclusion of a wider range of protocols and tendinopathies in our review.

Comparisons of exercise volume, commonly reported as the product of sets and repetitions, did not produce consistent results in our review. However, it is worth noting that meta-regressions investigating volume for RCRSP tendinopathies provided some evidence of increased effectiveness of higher volume exercise for both outcome domains producing large and small effect sizes. The included RCRSP studies commonly prescribed lower intensities of resistance for the upper limb with a focus on range of motion and mobility.^33–35^ A recent review by Malliaras^36^ found low quality evidence suggesting that higher volume and intensity exercise (or higher volume alone) may have superior functional outcomes compared with lower doses, but not for pain outcomes, in RCRSP tendinopathies. However, they were limited to just three studies due to their inclusion criteria and lack of clear reporting in the literature.^36^ Tendons of the shoulder facilitate repetitive movements of daily tasks with less overall load than larger weight bearing tendons like the Achilles and may require programmes that imitate that repetitive nature through higher volume of exercise.

### Limitations

We did not search Web of Science, however, together with our information scientist we are confident that our search was comprehensive and rigorous. We may have missed some potentially relevant high-quality studies by excluding those that were not from countries ranked ‘very high’ on the HDI (eg, Brazil and South Africa). However, this number was small and allowed us to generalise the findings to the UK. One of the limitations of this study, and a challenge for future evidence syntheses, is the lack of clear reporting. We found that in general, exercise volume and frequency were better reported, with reporting of intensity often poor. Similarly, our review identified that although load progression was frequently stated, studies rarely reported the actual loads or intensity used. The use of reporting guidelines such as Consolidated Standards of Reporting Trials^37^ or Consensus on Exercise Reporting Template^38^ in primary research would greatly enhance future reviews. Understandably, progressions are matched to individuals, however, more detailed reporting of loads prescribed and ultimately used across participants would be useful for future evidence syntheses and to better inform clinicians. Relative intensity measures such as per cent of maximum repetition are likely to provide the most useful information for future evidence syntheses and the most precise comparisons. Another limitation to note is that we extracted resistance dose data for the primary exercise, therefore other exercises prescribed as part of the wider intervention were not accounted for in analysis. This means that variables such as exercise volume may not be fully representative of the true volume of overall exercise performed, but rather specifically the dominant resistance exercise. Although most studies made clear which exercise was the focus of investigation, it was unclear in a small number and in these cases, we identified the exercise with the higher volume. The comparability of our RoB judgements with recent reviews using the updated RoB2 tool may be limited due to poor inter-tool reliability with the older version of Cochrane’s RoB tool.^22^ While we used RobotReviewer pragmatically for efficiency, we wish to point out that decisions were checked to improve accuracy and consistency.

An additional substantive limitation of this review includes the use of non-controlled effect sizes. This approach was adopted due to the ability to greatly increase the amount of data available and address limitations of previous reviews based largely on small sample sizes and subsequent decisions not to quantitatively synthesise results. The major limitation of this approach is the potential for unbalanced treatment moderators including a range of intervention and population characteristics to associate with different levels of the dose variables defined, thus biasing results. Given the likely interaction between intensity, volume and frequency, we attempted to control for these interactions by including a more complete meta-regression with all three variables included. While the results of the analysis supported those obtained with the individual meta-regressions, there are likely to be many other effect moderators including intervention duration, follow-up duration, adherence and baseline characteristics of patients^39 40^ that may have been imbalanced and could not be controlled. Additionally, in clinical settings there is potential for extreme values to occur and if these errors are asymmetric then regression to the mean effects can create poor estimates further limiting non-controlled effect sizes. In a previous analysis conducted with similar data we showed that effect size values are greatly influenced by outcome domains and could be summarised by a binary classification.^18^ As a result, we conducted analyses in the present review based on outcome domains that tended to generate large and small effects. However, it is possible that this binary classification represents too much of a simplification and imbalances in outcomes may also have biased results. Large increases in data were obtained by pooling results across the different tendinopathy locations. This approach was adopted based on our previous analysis indicating that the distribution of effect sizes following exercise therapy is likely to be similar.^18^ However, where sufficient data were available to conduct meta-regressions for individual tendinopathy locations, results tended to be consistent. Finally, another limitation of the review included the confidence in cumulative evidence which was most frequently identified as low and in a number of cases very low. This was predominantly due to extensive heterogeneity in studies resulting in issues of inconsistency and imprecision in effect size estimates.

## Conclusions and clinical implications

The results of this large systematic review with meta-analysis suggest that where resistance exercise is being prescribed for tendinopathy management, clinicians should consider whether a sufficient stimulus with regards to exercise intensity is being adopted and whether there is appropriate time for recovery. For certain patients this may require a substantive period of progression before reaching higher intensities. However, when appropriate, clinicians should consider prescribing higher intensities of resistance exercise through the application of external loads rather than just body mass; and given the increased loading, prescribing lower frequencies of sessions (less than daily) to allow for adequate recovery. Further refinement of the interrelations between exercise dose parameters and patient characteristics are required, including better understanding of the influence of exercise volume.
