# Supplementary material for: Effect of resistance exercise dose components for tendinopathy management: a systematic review with meta-analysis
Source: Br J Sports Med. 2023 May 11;57(20):1327–34. doi: 10.1136/bjsports-2022-105754 (PMC10579176; doi:10.1136/bjsports-2022-105754)
Supplement: Supplementary data [file bjsports-2022-105754supp002.pdf]

SF2: Definitions used to define exercise treatments and treatment classes.

**Treatment Class**	**Definition**	**Treatment**	**Definition**
Resistance	Exercise designed primarily to increase strength of muscles by causing them to produce substantive force against an applied resistance which can take several forms including the mass of the body or its segments, isoinertial resistance, elastic resistance, or strength training equipment such as isokinetic devices.	Concentric Only	Includes movements where force produced overcomes the resistance such that muscle shortening occurs.
		Eccentric Only	Includes movements where force produced is less than the resistance such that controlled muscle lengthening occurs.
		Concentric and eccentric	Includes movements where force produced exceeds the resistance in one phase and is less than the resistance in another such that controlled muscle lengthening and shortening occurs.
		Isokinetic	Uses specialised exercise equipment such that the resistance is adjusted in real-time to ensure joint angular velocity remains constant.
		Isometric	Includes muscular actions against a resistance such that joint angle remains constant.
