# Supplementary material for: Effect of resistance exercise dose components for tendinopathy management: a systematic review with meta-analysis
Source: Br J Sports Med. 2023 May 11;57(20):1327–34. doi: 10.1136/bjsports-2022-105754 (PMC10579176; doi:10.1136/bjsports-2022-105754)
Supplement: Supplementary data [file bjsports-2022-105754supp003.pdf]

BMJ Publishing Group Limited (BMJ) disclaims all liability and responsibility arising from any reliance placed on this supplemental material which has been supplied by the author(s)

SF3: Outcome domains and example outcomes included in review.

Domain	ICON Definition	Example Tools
Disability	Composite scores of a mix of patient-rated pain & disability due to the pain, usually relating to tendon-specific activities/tasks	VISA scales; DASH; quick DASH; SPADI; Patient-rated tennis-elbow evaluation questionnaire; Constant Murley Score; WORC (Western Ontario Rotator Cuff Index); AOFAS (American Orthopaedic Foot & Ankle Society); Roles and Maudsley score; ASES (American Shoulder & Elbow Surgeons Index); Hip & Groin Outcome Score; Foot & Ankle outcome score (FAOS)/Questionnaire (FAOQ); Oxford hip score (OHS); Hip disability & outcome OA score (HOOS); Tegner activity score; Lysholm knee scale; Pain free function questionnaire; Ankle activity score; Subjective elbow Value (SEV); Placzek score; Shoulder disability questionnaire; Foot function index (FFI); International Knee Documentation Committee form (IKDC); Penn Shoulder score (university of Pennsylvania shoulder score) (PSS); Brief pain inventory (BPI); UCLA Shoulder Rating Scale; FILLA - functional index of leg and lower limb; Neer Shoulder Score; Nirschl phase rating scale; Manchester–Oxford Foot Questionnaire (MOXFQ); American Shoulder and Elbow Surgeon's (MASES) questionnaire; Mayo Elbow Performance Score (MEPS); Shoulder rating questionnaire (SRQ);
Function	Participant/patient rated level of function (and not referring to the intensity of their pain; eg, Patient Specific Function Scale on a VAS or NRS).	Patient-specific functional scale
Pain on loading/activity	Patient reported intensity of pain performing a task that loads the tendon	VAS; NRS; Pain experience scale
Pain over a specified time	Patient-reported pain intensity over period of time e.g. morning/night/24-hours/1-week	VAS; NRS Painful days in 3 months
Pain without further specification	Patient asked about pain levels without reference to activity or timeframe	VAS; NRS; Borg CR10 Scale; Pain status
Physical function capacity	Quantitative measures of physical tasks (e.g. hops, times walk, single leg squat) includes muscle strength	Counter movement jump; one-leg triple hop; single-leg decline squat; muscle strength measured by dynamometry (hand-held, isokinetic); manual muscle testing.
Quality of Life	General wellbeing	EQ5D; EQ3D; SF-36 or SF-12; Assessment of Quality of Life (AQoL); Nottingham Health Profile; Gothenburg QoL Instrument
Range of Motion (shoulder only)	Active or passive range of motion in specified plane, measured in degrees.	Hand-held goniometer; inclinometer

VISA= Victorian Institute of Sport Assessment; DASH =Disabilities of the Arm, Shoulder and Hand; OA= osteoarthritis; VAS= visual analogue scale; NRS= Numerical Rating Scale.
