# Supplementary material for: Effect of resistance exercise dose components for tendinopathy management: a systematic review with meta-analysis
Source: Br J Sports Med. 2023 May 11;57(20):1327–34. doi: 10.1136/bjsports-2022-105754 (PMC10579176; doi:10.1136/bjsports-2022-105754)
Supplement: Supplementary data [file bjsports-2022-105754supp004.pdf]

SF4: Search strategies for each source

Embase (Ovid)	(exercise OR exercise*.mp OR "isometric exercise" OR kinesiotherapy OR Eccentric.mp OR concentric.mp OR "heavy slow resistance".mp OR "isokinetic exercise" OR plyometrics OR "muscle stretching" OR "muscle training") AND (tendinitis OR Tendinopathy.mp OR "tendon injury" OR "shoulder injury" OR "rotator cuff injury" OR "tennis elbow" OR tendin.mp OR tendon.mp OR bursitis OR "shoulder impingement syndrome" OR 2posterior tibial tendon dysfunction" OR "Greater trochanteric pain syndrome".mp)
CINAHL (EBSCO-host)	(MH Exercise OR AB exercise* OR MH "muscle strengthening" OR MH "rehabilitation" OR MH "eccentric contraction" OR TX "heavy slow resistance exercis*" OR AB eccentric OR AB concentric OR AB isokinetic OR MH "therapeutic exercise") AND (MH tendinopathy OR MH "arm injuries" OR "tendon injuries" OR MH tendons OR TX tendin* OR TX tendon* OR AB bursitis OR MH Bursitis OR MH "Posterior tibial tendon dysfunction" OR MH "shoulder impingement syndrome" OR AB "Greater trochanteric pain syndrome")
Medline (EBSCO-host)	(MH exercise OR AB exercise* OR MH "isometric contraction" OR MH rehabilitation OR TX eccentric OR TX concentric OR TX "heavy slow resistance" OR TX isokinetic) AND (MH tendinopathy OR MH "shoulder injuries" OR MH tendons OR MH "tendon injuries OR TX tendin* OR tendon* OR MH bursitis OR AB bursitis OR MH "posterior tibial tendon dysfunction" OR MH "shoulder impingement syndrome" OR AB "greater trochanteric pain syndrome")
SPORTDiscus (EBSCO-host)	(DE exercise OR DE "exercise therapy" OR AB exercise* OR TX eccentric OR TX concentric OR TX "heavy slow resistance" OR DE "isokinetic exercise" OR DE plyometrics OR DE "strength training" OR DE "stretch (physiology)" OR DE "isometric exercise" OR DE rehabilitation) AND (DE tendinitis OR DE tendinosis OR AB tendinopathy OR DE "tendon injuries" OR "shoulder injuries" OR DE "tennis elbow" OR AB tendin* OR AB tendon* OR DE bursitis OR AB "shoulder impingement syndrome" OR AB "posterior tibial tendon dysfunction" OR AB "greater trochanteric pain syndrome")
Amed (EBSCO-host)	(ZU exercise OR ZU "exercise therapy" OR AB exercise OR ZU "muscle stretching exercises" OR ZU "isometric contraction" OR ZU rehabilitation OR TZ eccentric OR TZ concentric OR TX "heavy slow resistance" OR TX isokinetic OR AB plyometric) AND (ZU tendinopathy OR ZU "tendon injuries" OR ZU tendons OR ZU "shoulder injuries" OR ZU "tennis elbow" OR TX tendin* OR TX tendon* OR ZU bursitis OR AB bursitis OR ZU "shoulder impingement syndrome" OR ZU "posterior tibial tendon dysfunction" OR AB "greater trochanteric pain syndrome")
JBI Evidence Synthesis	Tendinopathy AND exercise
Cochrane Library	Tendinopathy AND exercise

	Tendin* AND exercise<br>Tendon AND exercise
PEDro	Tendinopathy AND exercise<br>Tendin* AND exercise<br>Tendon AND exercise
Epistemonikos	(tendinopathy OR tendon* OR tendin*) AND exercise
Clinicialtrials.gov	Tendinopathy AND exercise<br>Tendin* AND exercise<br>Tendon AND exercise
ISRCTN	Tendinopathy AND exercise<br>Tendin* AND exercise<br>Tendon AND exercise
EU CTR	Tendinopathy AND exercise<br>Tendin* AND exercise<br>Tendon AND exercise
ANZCTR	Tendinopathy AND exercise<br>Tendin* AND exercise<br>Tendon AND exercise

ISRCTN – the Research Registry; EU CTN – European Clinical Trials Registry; ANZCTR – Australia and New Zealand Clinical Trials Registry.
