# Supplementary material for: Effect of resistance exercise dose components for tendinopathy management: a systematic review with meta-analysis
Source: Br J Sports Med. 2023 May 11;57(20):1327–34. doi: 10.1136/bjsports-2022-105754 (PMC10579176; doi:10.1136/bjsports-2022-105754)
Supplement: Supplementary data [file bjsports-2022-105754supp005.pdf]

SF5: Extraction codebook

Column		Heading	Description
**Study Details**	A	Initials Reviewer	Identification of individual extracting information
	B	Covidence Identifier	Reference number for Covidence
	C	Author	First author surname *et al.,*
	D	Year	Year of publication
	E	Title	Study title
	F	Country	Country where study was conducted
	G	Journal	Journal name
	H	Aims/Purpose	Study aims/purpose
	I	Tendinopathy type	1=Achilles; 2= Lateral elbow (tennis); 3 = Patellar; 4 = Rotator cuff related shoulder pain (RCRSP)
	J	Study Design	RCT = 1; Quasi-experimental = 2
	K	Age Mean	Mean age of study sample as a whole
	L	Age SD	Standard deviation age of study sample as a whole
	M	Baseline Total N	Total sample across all interventions measured at baseline
	N	Training Status Description	Brief description of training status of study sample as a whole
	O	Training Status Code	1 = Performance; 2 = Sporting; 3 = Other
	P	Sex	Percentage female of study sample as a whole
	Q	BMI Mean	Mean BMI of study sample as a whole
	R	BMI SD	Standard deviation of BMI of study sample as a whole
	S	Symptom Severity Mean	Mean severity measure at baseline of study sample as a whole
	T	Symptom Severity SD	Standard deviation of severity measure at baseline of study sample as a whole
	U	Symptom Duration Mean (Months)	Mean symptom duration reported in months
	V	Symptom Duration SD (Months)	Standard deviation symptom duration reported in months
	W	Population Comments	Any additional information relevant to the participants investigated including diagnostic criteria
**Outcomes**	X	Outcome Category	1 = Disability; 2 = Pain on loading/activity; 3 = Pain over a specified time; 4 = Pain without further specification; 5 = Physical function capacity; 6 = Participant/patient rating overall condition; 7) Participation; 8) Quality of life; 9) Range of motion
	Y	Outcome Tool	Description of outcome tool
	Z	Reflection	1 = Increase in outcome indicates positive treatment; -1 = Decrease in outcome indicates positive treatment
	AA	Measurement Time (Weeks)	Time of measurement in weeks
**Intervention**	AB	Dominant Treatment Class	**Only one dominant theme to be selected** 1 = Resistance; 2 = Plyometric; 3 = Vibration; 4 = Flexibility; 5 = Movement pattern retraining
	AC	Total Treatment class	**Multiple themes to be selected as required** 1 = Resistance; 2 = Plyometric; 3 = Vibration; 4 = Flexibility; 5 = Movement pattern retraining

	AD	Dose Comparison	1 = Lower dose intervention; 2 = Higher dose intervention
	AE	Intervention N	Intervention sample size at specified time
	AF	Intervention Total Duration	Total duration of exercise intervention in weeks
	AG	Intervention Adherence %	Reporting of adherence to exercise (reported as a percentage) if applicable
	AH	Intervention Location	Location exercise was performed<br>1 = Home; 2 = Clinic; 3 = Fitness facility; 4 = NR; 5 = NA
	AI	Intervention Volume	Numerical value describing volume
	AJ	Intervention Volume Category	1 = Duration of session (mins); 2 = sets * repetitions; 3 = number of repetitions; 4 = number of sets
	AK	Intervention Volume Comments	Any additional information relevant.
	AL	Intervention Intensity	Numerical value describing intensity
	AM	Intervention Intensity Category	1 = Absolute; 2 = Relative; 3=Bodyweight; 4=bodyweight+
	AN	Intervention Frequency	Number of sessions per week. Where there is progression, average value is to be entered.
	AO	Intervention Frequency Comments	Any additional information relevant.
	AP	Intervention Progression	**Multiple themes to be selected as required**<br>1 = No progression; 2 = NR; 3 = Progression volume; 4 = Progression intensity; 5 = Progression frequency; 6 = Progression specificity; 7 = Progression capacity; 8 = Other
	AQ	Intervention Progression Comments	Any additional information relevant.
**Data**	AR	Intervention Baseline Mean	Baseline mean for exercise therapy
	AS	Intervention Baseline SD	Baseline standard deviation for exercise therapy
	AT	Intervention Measurement Mean	Mean of outcome for exercise therapy at stated time point
	AU	Intervention Measurement SD	Standard deviation of outcome for exercise therapy at stated time point
	AV	Measurement Comments	State if a different value has been entered for means (e.g. median), a different value for standard deviations (e.g. standard error, IQR, percentiles, distance from mean to upper bound). Provide the relevant statistic (width of CI's, width of percentiles). Also state if data has extracted by digitization

* Outcome Specific. RCT=randomised controlled trial; SD=standard deviation; BMI= body mass index; NR= not reported; NA= not applicable; IQR=Inter Quartile Range; CI= confidence intervals.
