# Supplementary material for: Effect of resistance exercise dose components for tendinopathy management: a systematic review with meta-analysis
Source: Br J Sports Med. 2023 May 11;57(20):1327–34. doi: 10.1136/bjsports-2022-105754 (PMC10579176; doi:10.1136/bjsports-2022-105754)
Supplement: Supplementary data [file bjsports-2022-105754supp006.pdf]

SF6: Statistical Analysis

Note: This supplement should be read together with the 'Statistical Analysis' section in the main manuscript to ensure completeness of information.

The null model with no meta-regression terms was expressed as Level 1: $d_{ijk} = \beta_{0ijk} + e_{ijk}$; Level 2: $\beta_{0ijk} = \eta_{0jk} + r_{ijk}$; Level 3: $\eta_{0jk} = \theta_{0k} + u_{0jk}$; Level 4: $\theta_{0k} = \gamma_0 + v_{0k}$, where d_{ijk} is the observed effect size at measurement occasion $i\ (i = 1,2,\dots,I_{jk})$, from outcome $j\ (j = 1,2,\dots,J_k)$ and from study $k\ (k = 1,2,\dots,K)$. The random effects $e_{ijk} \sim N(0,\sigma_e^2), r_{ijk} \sim N(0,\sigma_r^2), u_{0jk} \sim N(0,\sigma_u^2)$ and $v_{0k} \sim N(0,\sigma_v^2)$ were assumed to be independent. The relative contributions of variance sources were described by variance partition coefficients (VPCs) calculated by dividing each estimated variance level by the total sum. Meta-analyses were conducted within a Bayesian framework providing additional flexibility in the handling of within study variances and enabled model estimates to be interpreted more intuitively through reporting of subjective probabilities.[1] Inferences in Bayesian analyses are generally made using credible intervals (CrI's) that provide more information than confidence intervals used in frequentist analyses that describe a uniform range of values that are plausible with the data.[1] In comparison CrI's can be interpreted probabilistically such that values at the centre are judged more probable than those at the tails. Similarly, inferences regarding the range of values a parameter may take (e.g., greater than zero) can be calculated and interpreted probabilistically within Bayesian analyses, compared to frequentist *p* values that do not provide information on the probability of parameter values.[1]

To assess the effects of dose variables, meta-regressions for d_{ijk} were performed with intensity (body mass vs. additional external) and volume (lower volume: <45 repetitions vs. ≥45 repetitions) comprising binary categorisations, and frequency (< once per day vs. once per day vs. > once per day) comprising a trinary categorisation. Meta-regressions were presented by selecting one level of the variable as a reference to make comparisons ($\beta_{\text{Reference:Comparison}}$ = Median [95% Credible Interval

(CrI): Lower Bound (LB) to Upper Bound (UB)], such that β>0 indicates an increased effect of the comparison relative to the reference). Based on previous analyses with a similar data set showing large differences in effect sizes across outcome types, separate meta-regressions were performed for outcomes typically generating large effect sizes (disability, function and pain), and small effect sizes (physical function capacity, range of motion and quality of life). Initially, meta-regressions were performed on data pooled across all tendinopathy locations. Sub-analyses were then performed with data from single tendinopathy locations where sufficient data were available. It was determined *a priori* that meta-regressions would only be performed when each level of the variable comprised a minimum of ten effect sizes from at least two studies. Inferences from all analyses were performed on posterior samples generated by Markov Chain Monte Carlo simulations and through use of credible intervals and probabilities calculated from the proportion of the posterior sample that met the given condition (e.g. β>0). Default weakly informative Student-t and Half-Student-t priors with 3 degrees of freedom and scale parameter equal to max{2.5, Median Absolute deviation(d_{ijk})} were used for location (γ_0, β) and variance parameters ($\sigma_r^2, \sigma_u^2, \sigma_v^2$), respectively.[2] Convergence of parameter estimates were obtained for all models with Gelman-Rubin R-hat values below 1.1.[3] Suitability of model assumptions were investigated by analysing standardised residuals by multiplying the difference in the observed value and median estimates of location parameters by the square root of the reciprocal of the total variance ($\sqrt{1/(\sigma_e^2 + \sigma_r^2 + \sigma_u^2 + \sigma_v^2)}\,(d_{ijk} - (\hat{\gamma}_0 + \hat{r}_{ijk} + \hat{u}_{0jk} + \hat{v}_{0k})$). Analyses were performed using the R wrapper package brms interfaced with Stan to perform sampling.[4]

References:

(1) Kruschke JK, Liddell TM. The Bayesian New Statistics: Hypothesis testing, estimation, meta-analysis, and power analysis from a Bayesian perspective. *Psychonomic bulletin & review; Psychon Bull Rev*. 2017; 25(1):178-206.

(2) Gelman A. Prior distributions for variance parameters in hierarchical models (comment on article by Browne and Draper). *Bayesian analysis*. 2006; 1(3):515-534.

(3) Gelman A, Carlin JB, Stern HS, Rubin DB. *Bayesian data analysis*. : Chapman and Hall/CRC; 1995. https://citeseerx.ist.psu.edu/viewdoc/download?doi=10.1.1.550.6951&rep=rep1&type=pdf

(4) Bürkner P. brms: An R package for Bayesian multilevel models using Stan. *Journal of statistical software*. 2017; 80(1):1-28.
