# Supplementary material for: Effect of resistance exercise dose components for tendinopathy management: a systematic review with meta-analysis
Source: Br J Sports Med. 2023 May 11;57(20):1327–34. doi: 10.1136/bjsports-2022-105754 (PMC10579176; doi:10.1136/bjsports-2022-105754)
Supplement: Supplementary data [file bjsports-2022-105754supp007.pdf]

SF7: Included studies

SF7A: Excluded studies with reasons

Reference	Reason for Exclusion
Abat F, Gelber PE, Polidori F, et al. 1 Clinical Results After EPI ® and Eccentric Exercise in Patellar Tendinopathy at 10 Years Follow-Up. Br J Sports Med 2014;48:A1. https://bjsm.bmj.com/lookup/doi/10.1136/bjsports-2014-094114.1 (accessed 12 Jun 2021).	Duplicate
Balius R, Álvarez G, Baró F, et al. A 3-Arm Randomized Trial for Achilles Tendinopathy: Eccentric Training, Eccentric Training Plus a Dietary Supplement Containing Mucopolysaccharides, or Passive Stretching Plus a Dietary Supplement Containing Mucopolysaccharides. Curr Ther Res 2016;78:1–7.	Duplicate
Blume CL. Comparison of an eccentric exercise intervention to a concentric exercise intervention in adults with subacromial impingement syndrome (Doctoral dissertation, Texas Woman's University). 2014:1-218.	Duplicate
Cannell LJ, Taunton JE, Clement DB, et al. A randomised clinical trial of the efficacy of drop squats or leg extension/leg curl exercises to treat clinically diagnosed jumper's knee in athletes: pilot study. Br J Sports Med 2001;35:60-64.	Duplicate
de Vos RJ, Weir A, van Schie HT, et al. Platelet-rich plasma injection for chronic Achilles tendinopathy. J - Am Med Assoc 2010;303:144–9.	Duplicate
Frohm A, Saartok T, Halvorsen K, et al. Eccentric treatment for patellar tendinopathy: a prospective randomised short-term pilot study of two rehabilitation protocols. Br J Sports Med 2007;41:e7.	Duplicate
Ganderton C, Semciw A, Cook J, et al. Gluteal loading versus sham exercises to improve pain and dysfunction in postmenopausal women with greater trochanteric pain syndrome: a randomized controlled trial. J Women's Heal 2018;27:815–29.	Duplicate
Heron SR, Woby SR, Thompson DP. Comparison of three types of exercise in the treatment of rotator cuff tendinopathy/shoulder impingement syndrome: A randomized controlled trial. Physiotherapy 2017;103:167–73.	Duplicate
Jensen B, Bliddal H, Danneskiold-Samsøe B. Comparison of two different treatments of lateral humeral epicondylitis--" tennis elbow". A randomized controlled trial. Ugeskr Laeg 2001;1:1427-31.	Duplicate
Jonsson P, Alfredson H. Superior results with eccentric compared to concentric quadriceps training in patients with jumper's knee: a prospective randomised study. Br J Sports Med 2005;39:847–50	Duplicate
Manias P, Stasinopoulos D. A controlled clinical pilot trial to study the effectiveness of ice as a supplement to the exercise programme for the	Duplicate

management of lateral elbow tendinopathy. Br J Sports Med 2006;40:81-85.	
Senbursa G, Baltacı G, Atay A. Comparison of conservative treatment with and without manual physical therapy for patients with shoulder impingement syndrome: a prospective, randomized clinical trial. Knee Surg Sports Traumatol Arthrosc. 2007;15:915-921.	Duplicate
Stergioulas A, Stergioula M, Aarskog R, et al. Effects of low-level laser therapy and eccentric exercises in the treatment of recreational athletes with chronic achilles tendinopathy. Am J Sports Med 2008;36:881–7.	Duplicate
Tumilty S, Baxter GD. Heavy load eccentric exercise for Achilles tendinopathy; too much of a good thing?. Physiotherapy 2015;101:e1546-1547.	Duplicate
van Ark M. Patellar tendinopathy: Physical therapy and injection treatments (Doctoral dissertation, University of Groningen).2015:1-136.	Duplicate
Walther M, Werner A, Stahlschmidt T, et al. The subacromial impingement syndrome of the shoulder treated by conventional physiotherapy, self-training, and a shoulder brace: results of a prospective, randomized study. J Shoulder Elbow Surg 2004;1:417-23.	Duplicate
Wetke E, Johannsen F, Langberg H. A hilles tendinopathy: A prospective study on the effect of active rehabilitation and steroid injections in a clinical setting. Scan J Med Sci Sports 2015;25:e392-399.	Duplicate
Aceituno-Gómez J, Avendaño-Coy J, Gómez-Soriano J, et al. Efficacy of high-intensity laser therapy in subacromial impingement syndrome: a three-month follow-up controlled clinical trial. Clin Rehabil 2019;33:894-903.	Insufficient exercise data
Akgün K, Birtane M, Akarirmak U. Is local subacromial corticosteroid injection beneficial in subacromial impingement syndrome? Clin Rheumatol 2004;23:496–500.	Insufficient exercise data
Akkaya N, Akkaya S, Gungor HR, et al. Effects of weighted and un-weighted pendulum exercises on ultrasonographic acromiohumeral distance in patients with subacromial impingement syndrome. J Back Musculoskelet Rehabil 2017;30:221-228.	Insufficient exercise data
Akkurt HE, Kocabas H, Yilmaz H, et al. Comparison of an epicondylitis bandage with a wrist orthosis in patients with lateral epicondylitis. Prosthet Orthot Int 2018;42:599–605.	Insufficient exercise data
Al Dajah SB. Soft tissue mobilization and PNF improve range of motion and minimize pain level in shoulder impingement. J Phys Ther Sci 2014;26:1803–5.	Insufficient exercise data
Alfredson H, Lorentzon R. Intratendinous glutamate levels and eccentric training in chronic Achilles tendinosis: a prospective study using microdialysis technique. Knee Surg Sports Traumatol Arthrosc 2003;11(3):196-199.	Insufficient exercise data
Apostolos S. The influence of low level laser and pyrometric exercises in the treatment of patients with tennis elbow. a pilot study. 2004. http://cev.org.br/biblioteca/the-influence-of-low-level-laser-and-	Insufficient exercise data

plyometric-exercises-in-the-treatment-of-patients-with-tennis-elbow-pilot-study/ (accessed 21 Jun 2021)	
Aytar A, Baltaci G, Uhl TL, et al. The effects of scapular mobilization in patients with subacromial impingement syndrome: a randomized, double-blind, placebo-controlled clinical trial. J Sport Rehabil 2015;24:116–29.	Insufficient exercise data
Bae YH, Lee GC, Shin WS, et al. Effect of motor control and strengthening exercises on pain, function, strength and the range of motion of patients with shoulder impingement, syndrome. Journal of Physical Therapy Science 2011;23:687-92.	Insufficient exercise data
Bal A, Eksioglu E, Gurcay E, et al. Low-level laser therapy in subacromial impingement syndrome. Photomed Laser Surg 2009;27:31–6.	Insufficient exercise data
Bang MD, Deyle GD. Comparison of supervised exercise with and without manual physical therapy for patients with shoulder impingement syndrome. J Orthop Sports Phys Ther 2000;30:126-137.	Insufficient exercise data
Barra López ME, López de Celis C, Fernández Jentsch G, et al. Effectiveness of Diacutaneous Fibrolysis for the treatment of subacromial impingement syndrome: a randomised controlled trial. Man Ther 2013;18:418–24.	Insufficient exercise data
Başkurt F, Özcan A, Algun C. Comparison of effects of phonophoresis and iontophoresis of naproxen in the treatment of lateral epicondylitis. Clin Rehabil 2003;17:96–100.	Insufficient exercise data
Başkurt Z, Başkurt F, Gelecek N, et al. The effectiveness of scapular stabilization exercise in the patients with subacromial impingement syndrome. J Back Musculoskelet Rehabil 2011;24:173-179.	Insufficient exercise data
Baumer TG, Peltz CD, Drake A, et al. Effects of Rotator Cuff Pathology and Physical Therapy on In Vivo Shoulder Motion and Clinical Outcomes in Patients With a Symptomatic Full-Thickness Rotator Cuff Tear. Orthop J Sports Med 2016;4: 2325967116666506.	Insufficient exercise data
Bisset L, Beller E, Jull G, et al. Mobilisation with movement and exercise, corticosteroid injection, or wait and see for tennis elbow: Randomised trial. Br Med J 2006;333:939–41.	Insufficient exercise data
Bisset L, Yelland M, Ryan M, et al. Testing the effectiveness of emerging injection therapies compared to physiotherapy for tennis elbow: a randomised control trial. Physiotherapy 2015;101:e155.	Insufficient exercise data
Bisset LM, Coppieters MW, Vicenzino B. Sensorimotor deficits remain despite resolution of symptoms using conservative treatment in patients with tennis elbow: A randomized controlled trial. Arch Phys Med Rehabil 2009;90:1–8.	Insufficient exercise data
Bostrøm K, Mæhlum S, Småstuen MC, et al. Clinical comparative effectiveness of acupuncture versus manual therapy treatment of lateral epicondylitis: feasibility randomized clinical trial. Pilot feasibility Stud 2019;5:110.	Insufficient exercise data
Branson R, Naidu K, du Toit C, et al. Comparison of corticosteroid, autologous blood or sclerosant injections for chronic tennis elbow. J Sci Med Sport 2017;20:528-533.	Insufficient exercise data

Brown R, Orchard J, Kinchington M, et al. Aprotinin in the management of Achilles tendinopathy: a randomised controlled trial. Br J Sports Med 2006;40:275-279.	Insufficient exercise data
Brown R, Orchard J, Kinchington M, et al. Aprotinin in the management of Achilles tendinopathy: a randomised controlled trial. Br J Sports Med 2006;40:275–9.	Insufficient exercise data
Brox JI, Gjengedal E, Uppheim G, et al. Arthroscopic surgery versus supervised exercises in patients with rotator cuff disease (stage II impingement syndrome): a prospective, randomized, controlled study in 125 patients with a 2 1/2-year follow-up. J Shoulder Elbow Surg 1999;8:102-111.	Insufficient exercise data
Calis HT, Berberoglu N, Calis M. Are ultrasound, laser and exercise superior to each other in the treatment of subacromial impingement syndrome? A randomized clinical trial. Eur J Phys Rehabil Med 2011;47:375-380.	Insufficient exercise data
Canbulat N, Seyahi A, Eren SM, et al. 24. The effect of core stabilization exercises in the rehabilitation of patients with subacromial impingement syndrome [Abstract]. Türkiye Fiz Tıp ve Rehabil Derg 2013;59:431.	Insufficient exercise data
Chapman-Jones D, Hill D. Novel microcurrent treatment is more effective than conventional therapy for chronic Achilles tendinopathy: randomised comparative trial. Physiotherapy 2002;1:471-80.	Insufficient exercise data
Cherry E, Agostinucci J, McLinden J. The effect of cryotherapy and exercise on lateral epicondylitis: a controlled randomised study. Int J Ther Rehabil 2012;19:641-650.	Insufficient exercise data
Chung B, Wiley JP, Rose MS. Long-term effectiveness of extracorporeal shockwave therapy in the treatment of previously untreated lateral epicondylitis. Clin J Sport Med 2005;15:305–12.	Insufficient exercise data
Citaker S, Taskiran H, Akdur H, et al. Comparison of the mobilization and proprioceptive neuromuscular facilitation methods in the treatment of shoulder impingement syndrome. Pain Clin 2005;17:197–202.	Insufficient exercise data
Cloke DJ, Watson H, Purdy S, et al. A pilot randomized, controlled trial of treatment for painful arc of the shoulder. J Shoulder Elbow Surg 2008;17:S17-21.	Insufficient exercise data
Coff L, Massy-Westropp N, Caragianis S. Randomized controlled trial of a new electrical modality (InterX) and soft tissue massage, stretching, ultrasound and exercise for treating lateral epicondylitis. Hand Ther 2009;14:46-52.	Insufficient exercise data
Cook C, Learman K, Houghton S, et al. The addition of cervical unilateral posterior–anterior mobilisation in the treatment of patients with shoulder impingement syndrome: A randomised clinical trial. Man Ther 2014;19:18–24 .	Insufficient exercise data
Coombes BK, Connelly L, Bisset L, et al. Economic evaluation favours physiotherapy but not corticosteroid injection as a first-line intervention for chronic lateral epicondylalgia: evidence from a randomised clinical trial. Br J Sports Med 2016;50:1400–5.	Insufficient exercise data
Crawshaw DP, Helliwell PS, Hensor EMA, et al. Exercise therapy after corticosteroid injection for moderate to severe shoulder pain: large pragmatic randomised trial. BMJ 2010;340:e3037–e3037.	Insufficient exercise data

Croisier JL, Forthomme B, Foidart-Dessalle M, et al. Isokinetic eccentric exercises in treating chronic tendinitis [Abstract]. Isokinet Exerc Sci 2002;10:25–6.	Insufficient exercise data
de Jonge S, de Vos J. R, Weir A, et al. One-year follow-up of platelet-rich plasma treatment in chronic Achilles tendinopathy: a double-blind randomized placebo-controlled trial. Am J Sports Med 2011;39:1623–9.	Insufficient exercise data
De Jonge S, de Vos RJ, Van Schie HTM, et al. One-year follow-up of a randamised controlled trial on added splinting to eccentric exercises in chronic midportion Achilles tendinopathy. Br J Sports Med 2010;44:673-677.	Insufficient exercise data
de Oliveira, FC L, Pairot de Fontenay B, Bouyer LJ, et al. Kinesiotaping for the Rehabilitation of Rotator Cuff-Related Shoulder Pain: A Randomized Clinical Trial. Sports health 2021;13:161-72.	Insufficient exercise data
Devereaux M, Velanoski KQ, Pennings A, et al. Short-Term Effectiveness of Precut Kinesiology Tape Versus an NSAID as Adjuvant Treatment to Exercise for Subacromial Impingement: A Randomized Controlled Trial. Clin J Sport Med 2016;26:24-32.	Insufficient exercise data
Di Lorenzo L, Pappagallo M, Gimigliano R, et al. Pain relief in early rehabilitation of rotator cuff tendinitis: any role for indirect suprascapular nerve block? Eura Medicophys 2006;42:195–204.	Insufficient exercise data
Dickens VA, Williams JL, Bhamra MS. Role of physiotherapy in the treatment of subacromial impingement syndrome: a prospective study. Physiotherapy 2005;91:159–64.	Insufficient exercise data
Dilek B, Gulbahar S, Gundogdu M, et al. Efficacy of Proprioceptive Exercises in Patients with Subacromial Impingement Syndrome: A Single-Blinded Randomized Controlled Study. Am J Phys Med Rehabil 2016;95:169–82.	Insufficient exercise data
Dragoo JL, Braun HJ, Wasterlain AS. Platelet-Rich Plasma as a Treatment for Patellar Tendinopathy: A Double-Blind Randomized Controlled Trial. Am J Sports Med 2014;42:610-618.	Insufficient exercise data
Dragoo JL, Wasterlain AS, Braun HJ, et al. Platelet-Rich Plasma as a Treatment for Patellar Tendinopathy. Am J Sports Med 2014;42:610–8.	Insufficient exercise data
Elsodany AM, Alayat MSM, Ali MME, et al. Long-Term Effect of Pulsed Nd:YAG Laser in the Treatment of Patients with Rotator Cuff Tendinopathy: A Randomized Controlled Trial. Photomed Laser Surg 2018;36:506–13.	Insufficient exercise data
Engebretsen K, Grotle M, Bautz-Holter E, et al. Radial extracorporeal shockwave treatment compared with supervised exercises in patients with subacromial pain syndrome: single blind randomised study. BMJ 2009;339.	Insufficient exercise data
Engebretsen K, Grotle M, Bautz-Holter E, et al. Supervised exercises compared with radial extracorporeal shock-wave therapy for subacromial shoulder pain: 1-year results of a single-blind randomized controlled trial. Phys Ther 2011;91:37-47.	Insufficient exercise data
Entrellardat Tortillol E. Effectiveness of percutaneus needle electrolysis and eccentric exercise in chronic patellar tendinopathy. Rev Fisioter Invasiva / J Invasive Tech Phys Ther 2019;02:75.	Insufficient exercise data

Eraslan L, Baltaci G, Yuce D, et al. Effects of Physiotherapy Approaches on Pain and Strength in Lateral Epicondylitis: A Randomized Clinical Trial [abstract]. Med Sci Sport Exerc 2015;47:614.	Insufficient exercise data
Farfaras S, Sernert N, Hallström E, et al. Comparison of open acromioplasty, arthroscopic acromioplasty and physiotherapy in patients with subacromial impingement syndrome: a prospective randomised study. Knee Surgery, Sport Traumatol Arthrosc 2016;24:2181–91.	Insufficient exercise data
Furia JP. High-energy extracorporeal shock wave therapy as a treatment for insertional Achilles tendinopathy. Am J Sports Med 2006;34:733-740.	Insufficient exercise data
Giombini A, Di Cesare A, Safran MR, et al. Short-term Effectiveness of Hyperthermia for Supraspinatus Tendinopathy in Athletes. Am J Sports Med 2006;34:1247–53.	Insufficient exercise data
González PP, Brahim MB. Treatment of Shoulder Impingement Syndrome in Adolescent Tennis Players. / Tractament de la síndrome subacromial en tennistes adolescents. Apunt Educ Física i Esports 2018;132:32–47.	Insufficient exercise data
Granviken F, Vasseljen O. Home exercises and supervised exercises are similarly effective for people with subacromial impingement: a randomised trial. Journal of Physiotherapy 2015;61:135-141.	Insufficient exercise data
Grävare Silbernagel K, Crossley KM. A proposed return-to-sport program for patients with midportion Achilles tendinopathy: rationale and implementation. journal of orthopaedic & sports physical therapy. 2015 Nov;45(11):876-86.	Insufficient exercise data
Güler H, Turhanoğlu AD, Inanoğllu K, et al. Comparison of ketoprofen phonophoresis with ketoprofen and lidocaine- prilocaine phonophoresis in patients with subacromial impingement syndrome. Turkish J Rheumatol 2009;24:88–93.	Insufficient exercise data
Gunay Ucurum S, Kaya DO, Kayali Y, et al. Comparison of different electrotherapy methods and exercise therapy in shoulder impingement syndrome: A prospective randomized controlled trial. Acta Orthop Traumatol Turc 2018;52:249–55.	Insufficient exercise data
Haahr JP, Andersen JH. Exercises may be as efficient as subacromial decompression in patients with subacromial stage II impingement: 4-8-years' follow-up in a prospective, randomized study. Scand J Rheumatol 2006;35:224–8.	Insufficient exercise data
Haahr JP, Andersen JH. Prognostic factors in lateral epicondylitis: A randomized trial with one-year follow-up in 266 new cases treated with minimal occupational intervention of the usual approach in general practice. Rheumatology 2003;42:1216–25.	Insufficient exercise data
Hernández Herrero D, Berjillos Donamayor A, de la Corte Rodríguez H, et al. Elbow tendinosis treated by several electrotherapy techniques: a prospective randomized study. 2006;4:131–138	Insufficient exercise data
Jensen B, Bliddal H, Danneskiold-Samsøe B. Comparison of two different treatments of lateral humeral epicondylitis--" tennis elbow". A randomized controlled trial. Ugeskr Laeg 2001;163:1427-1431.	Insufficient exercise data
Jerosch J, Wustner P. The effect of a sensorimotor exercise program in patients with subacromial pain syndrome. Unfallchirurg 2002;105:36–43.	Insufficient exercise data

Johansson K, Bergström A, Schröder K, et al. Subacromial corticosteroid injection or acupuncture with home exercises when treating patients with subacromial impingement in primary care--a randomized clinical trial. Fam Pract 2011;28:355–65.	Insufficient exercise data
Jonsson P. Eccentric training in the treatment of tendinopathy (Doctoral dissertation, Department of Surgical and Perioperative Sciences, Sports Medicine). 2009.	Insufficient exercise data
Jowett S, Crawshaw DP, Helliwell PS, et al. Cost-effectiveness of exercise therapy after corticosteroid injection for moderate to severe shoulder pain due to subacromial impingement syndrome: a trial-based analysis. Rheumatology 2013;52:1485–91.	Insufficient exercise data
Juul-Kristensen B, Larsen CM, Eshoj H, et al. Positive effects of neuromuscular shoulder exercises with or without EMG-biofeedback, on pain and function in participants with subacromial pain syndrome - A randomised controlled trial. J Electromyogr Kinesiol 2019;48:161-168.	Insufficient exercise data
Kaya E, Zinnuroglu M, Tugcu I. Kinesio taping compared to physical therapy modalities for the treatment of shoulder impingement syndrome. Clin Rheumatol 2011;30:201–7.	Insufficient exercise data
Kesikburun S, Tan AK, Yilmaz B, et al. Platelet-rich plasma injections in the treatment of chronic rotator cuff tendinopathy: a randomized controlled trial with 1-year follow-up. Am J Sports Med 2013;41:2609–16.	Insufficient exercise data
Ketola S, Lehtinen J, Elo P, et al. No difference in long-term development of rotator cuff rupture and muscle volumes in impingement patients with or without decompression: A randomized MRI study of 140 patients. Acta Orthop 2016;87:351–5.	Insufficient exercise data
Ketola S, Lehtinen J, Rousi T, et al. No evidence of long-term benefits of arthroscopic acromioplasty in the treatment of shoulder impingement syndrome. Bone & Joint Research 2013;2:132-139.	Insufficient exercise data
Ketola S, Lehtinen J, Rousi T, et al. Which patients do not recover from shoulder impingement syndrome, either with operative treatment or with nonoperative treatment? Acta Orthop 2015;86:641–6.	Insufficient exercise data
Ketola S, Lehtinen JT, Arnala I. Arthroscopic decompression not recommended in the treatment of rotator cuff tendinopathy. Bone Joint J 2017;99-B:799–805.	Insufficient exercise data
Kim J, Lee SC, Chun Y, et al. Effects of a 4-Week Short-Foot Exercise Program on Gait Characteristics in Patients With Stage II Posterior Tibial Tendon Dysfunction. J Sport Rehabil 2020;30:120-128.	Insufficient exercise data
Kim J, Shin D, Song C. Visual feedback to improve the effects of scapular stabilization exercises on pain intensity, range of motion, strength, and disability in patients with shoulder impingement syndrome. Medical Science Technology 2017;58:42-48.	Insufficient exercise data
Kim S, Kwon O, Weon J, et al. The effect of the neurac training on shoulder isokinetic performance in patients with acute-phase subacromial impingement syndrome [Absract]. Man Ther 2016;25:e59 https://www.infona.pl/resource/bwmeta1.element.elsevier-7217cec5-508b-3ebf-bb33-8aef41a575e1 (accessed 01 Jul 2021)	Insufficient exercise data

Kim SJ, Yeo SM, Noh SJ, et al. Effect of platelet-rich plasma on the degenerative rotator cuff tendinopathy according to the compositions. J Orthop Surg Res 2019;14:408.	Insufficient exercise data
Kim SY, Dvir Z, Oh JS. The application of the Neurac technique vs. manual therapy in patients during the acute phase of subacromial impingement syndrome: A randomized single-blinded controlled trial. J Back Musculoskelet Rehabil2019.	Insufficient exercise data
Kolk A, Auw Yang KG, Tamminga R, et al. Radial extracorporeal shock-wave therapy in patients with chronic rotator cuff tendinitis: a prospective randomised double-blind placebo-controlled multicentre trial. Bone Joint J 2013;95:1521-6.	Insufficient exercise data
Krogh TP, Ellingsen T, Christensen R, et al. Ultrasound-guided injection therapy of Achilles tendinopathy with platelet-rich plasma or saline: a randomized, blinded, placebo-controlled trial. Am J Sports Med 2016;44:1990-1997.	Insufficient exercise data
Kumar N, Nehru A, Rajalakshmi D. Effect of taping as a component of conservative treatment for subacromial impingement syndrome. Health 2012;26:237-241.	Insufficient exercise data
Kvalvaag E, Brox JI, Engebretsen KB, et al. Effectiveness of radial extracorporeal shock wave therapy (rESWT) when combined with supervised exercises in patients with subacromial shoulder pain. Am J Sports Med 2017;45:2547–54.	Insufficient exercise data
Kvalvaag E, Roe C, Engebretsen KB, et al. One year results of a randomized controlled trial on radial Extracorporeal Shock Wave Treatment, with predictors of pain, disability and return to work in patients with subacromial pain syndrome. Eur J Phys Rehabil Med 2018;54:341–50.	Insufficient exercise data
Leduc BE, Caya J, Tremblay S, et al. Treatment of calcifying tendinitis of the shoulder by acetic acid iontophoresis: a double-blind randomized controlled trial. Arch Phys Med Rehabil 2003;84:1523–7.	Insufficient exercise data
Littlewood C, Malliaras P, Mawson S, et al. Self-managed loaded exercise versus usual physiotherapy treatment for rotator cuff tendinopathy: a pilot randomised controlled trial. Physiotherapy 2014;100:54–60.	Insufficient exercise data
López-de-Celis C, Barra-López ME, González-Rueda V, et al. Effectiveness of diacutaneous fibrolysis for the treatment of chronic lateral epicondylalgia: a randomized clinical trial. Clin Rehabil 2018;32:644–53.	Insufficient exercise data
Ludewig PM, Borstad JD. Effects of a home exercise programme on shoulder pain and functional status in construction workers. Occup Environ Med 2003;60:841-849.	Insufficient exercise data
Melegati G, Tornese D, Bandi M. Effectiveness of extracorporeal shock wave therapy associated with kinesitherapy in the treatment of subacromial impingement: A randomised, controlled study. Journal of Sports Traumatology and Related Research 2000;22:58-64.	Insufficient exercise data
Mellor R, Bennell K, Grimaldi A, et al. Education plus exercise versus corticosteroid injection use versus a wait and see approach on global outcome and pain from gluteal tendinopathy: prospective, single blinded, randomised clinical trial. Br J Sports Med 2018;52:1464–72.	Insufficient exercise data

Miccinilli S, Bravi M, Morrone M, et al. A Triple Application of Kinesio Taping Supports Rehabilitation Program for Rotator Cuff Tendinopathy: a Randomized Controlled Trial. Ortop Traumatol Rehabil 2018;20:499–505.	Insufficient exercise data
Munteanu SE, Scott LA, Bonanno DR, et al. Effectiveness of customised foot orthoses for Achilles tendinopathy: a randomised controlled trial. Br J Sports Med 2015;49:989–94.	Insufficient exercise data
Newcomer KL, Laskowski ER, Idank DM, et al. Corticosteroid injection in early treatment of lateral epicondylitis. Clin J Sport Med 2001;11:214–22.	Insufficient exercise data
Nishizuka T, Iwatsuki K, Kurimoto S, et al. Efficacy of a forearm band in addition to exercises compared with exercises alone for lateral epicondylitis: A multicenter, randomized, controlled trial. J Orthop Sci 2017;22:289-294.	Insufficient exercise data
O'Neill S, Watson P, Barry S. Eccentric rehabilitation for runners with Achilles tendinopathy improves endurance capacity of the plantarflexors [Abstract]. Physiotherapy 2015;101:e1143–4.	Insufficient exercise data
Ohberg L, Lorentzon R, Alfredson H. Eccentric training in patients with chronic Achilles tendinosis: normalised tendon structure and decreased thickness at follow up. Br J Sports Med 2004;38:8–11.	Insufficient exercise data
Oken O, Kahraman Y, Ayhan F, et al. The Short-term Efficacy of Laser, Brace, and Ultrasound Treatment in Lateral Epicondylitis: A Prospective, Randomized, Controlled Trial. J Hand Ther 2008;21:63–8.	Insufficient exercise data
Østerås H, Torstensen TA, Haugerud L, Østerås BS. Dose–response effects of graded therapeutic exercises in patients with long-standing subacromial pain. Advances in physiotherapy. 2009 Jan 1;11(4):199-209.	Insufficient exercise data
Özgen M, Fırat S, Sarsan A, et al. Short- and long-term results of clinical effectiveness of sodium hyaluronate injection in supraspinatus tendinitis. Rheumatol Int 2012;32:137–44.	Insufficient exercise data
Paavola M, Malmivaara A, Taimela S, et al. Subacromial decompression versus diagnostic arthroscopy for shoulder impingement: randomised, placebo surgery controlled clinical trial. BMJ 2018;19:362.	Insufficient exercise data
Paoloni JA, Appleyard RC, Nelson J, et al. Topical Glyceryl Trinitrate Treatment of Chronic Noninsertional Achilles Tendinopathy. J Bone Jt Surg 2004;86:916–22.	Insufficient exercise data
Pasin T, Ataoglu S, Pasin O, et al. Comparison of the effectiveness of platelet-rich plasma, corticosteroid, and physical therapy in subacromial impingement syndrome. Arch Rheumatol 2019;34:308–16.	Insufficient exercise data
Pienimaki T, Karinen P, Kemila T, et al. Long-term follow-up of conservatively treated chronic tennis elbow patients. A prospective and retrospective analysis. Scand J Rehabil Med 1998;30:159–66.	Insufficient exercise data
Polimeni V, Panuccio A, Furfari P, et al. Preliminary study on the efficacy of various rehabilitation therapies for shoulder pain. Europa Medicophysica 2003;39:59-63.	Insufficient exercise data
Prat PI, Cibrowski D, Zuliani A, et al. Efficacy of fascial manipulation and eccentric exercise for lateral elbow pain. J Bodyw Mov Ther 2018;22:855.	Insufficient exercise data

Ram R, Meeuwisse W, Patel C, et al. The Limited Effectiveness of a Home-Based Eccentric Training for Treatment of Achilles Tendinopathy. Clin Investig Med 2013;36:197.	Insufficient exercise data
Rasmussen S, Christensen M, Mathiesen I, et al. Shockwave therapy for chronic Achilles tendinopathy: a double-blind, randomized clinical trial of efficacy. Acta Orthop 2008;79:249-256.	Insufficient exercise data
Razavi M, Jansen GB. Effects of acupuncture and placebo TENS in addition to exercise in treatment of rotator cuff tendinitis. Clin Rehabil 2004;18:872–8.	Insufficient exercise data
Reyhan AC, Sindel D, Dereli EE. The effects of Mulligan's mobilization with movement technique in patients with lateral epicondylitis. J Back Musculoskelet Rehabil2020;33:99-107.	Insufficient exercise data
Riley SP, Cote MP, Leger RR, et al. Short-term effects of thoracic spinal manipulations and message conveyed by clinicians to patients with musculoskeletal shoulder symptoms: a randomized clinical trial. J Man Manip Ther 2015;23:3–11.	Insufficient exercise data
Rio E, Kidgell D, Purdam C, et al. Isometric exercise induces analgesia and reduces inhibition in patellar tendinopathy. Br J Sports Med 2015;49:1277–1283.	Insufficient exercise data
Romero-Morales C, Martín-Llantino PJ, Calvo-Lobo C, et al. Effectiveness of eccentric exercise and a vibration or cryotherapy program in enhancing rectus abdominis muscle thickness and inter-rectus distance in patients with chronic mid-portion achilles tendinopathy: A randomized clinical trial. Int J Med Sci 2018;15:1764-1770.	Insufficient exercise data
Saggini R, Di Stefano A, Galati V, et al. Long-term effectiveness of combined mechanotransduction treatment in jumper's knee. Eur J Inflamm 2012;10:515–24.	Insufficient exercise data
Schmitt J, Haake M, Tosch A, et al. Low-energy extracorporeal shock-wave treatment (ESWT) for tendinitis of the supraspinatus: a prospective, randomised study. J Bone Surg Joint Am 2001;83:873-876.	Insufficient exercise data
Selvanetti A, Barrucci A, Antonaci A, et al. L'esercizio eccentrico nella rieducazione funzionale dell' epicondilite: studio randomizzato controllato (Role of the eccentric exercise in the functional reeducation of lateral epicondylitis: a randomised controlled clinical trial) [Italian]. Med Dello Sport 2003;56:103–13.	Insufficient exercise data
Seven MM, Ersen O, Akpancar S, et al. Effectiveness of prolotherapy in the treatment of chronic rotator cuff lesions. Orthop Traumatol Surg Res 2017;103:427-433.	Insufficient exercise data
Speed CA, Richards C, Nichols D, Burnet S, Wies JT, Humphreys H, Hazleman BL. Extracorporeal shock-wave therapy for tendonitis of the rotator cuff: a double-blind, randomised, controlled trial. The Journal of Bone and Joint Surgery. British volume. 2002 May;84(4):509-12.	Insufficient exercise data
Stefanou A, Marshall N, Holdan W, et al. A randomized study comparing corticosteroid injection to corticosteroid iontophoresis for lateral epicondylitis. J Hand Surg Am 2012; 37:104-109.	Insufficient exercise data
Struijs PAA, Korthals-de Bos IBC, van Tulder MW, et al. Cost effectiveness of brace, physiotherapy, or both for treatment of tennis elbow. Br J Sports Med 2006;40:637–43.	Insufficient exercise data

Struyf F, Nijs J, Mollekens S, et al. Scapular-focused treatment in patients with shoulder impingement syndrome: a randomized clinical trial. Clin Rheumatol 2013;32:73–85.	Insufficient exercise data
Subaşı V, Çakır T, Arıca Z, et al. Comparison of efficacy of kinesiological taping and subacromial injection therapy in subacromial impingement syndrome. Clin Rheumatol 2016;35:741–6.	Insufficient exercise data
Subaşi V, Toktaş H, Demirdal ÜS, et al. Water-Based versus Land-Based Exercise Program for the Management of Shoulder Impingement Syndrome. / Omuz Subakromiyal Sıkışma Sendromunun Tedavisinde Su İçi Egzersizler ile Kara Egzersizlerinin Karşılaştırılması. Turkish J Phys Med Rehabil 2012;58:79–84.	Insufficient exercise data
Szczurko O, Cooley K, Mills EJ, et al. Naturopathic treatment of rotator cuff tendinitis among Canadian postal workers: A randomized controlled trial. Arthritis Rheum 2009;61:1037–45.	Insufficient exercise data
Tahran Ö, Yeşilyaprak SS. Effects of Modified Posterior Shoulder Stretching Exercises on Shoulder Mobility, Pain, and Dysfunction in Patients With Subacromial Impingement Syndrome. Sports health 2020;12:139-148.	Insufficient exercise data
Taskaynatan MA, Ozgul A, Ozdemir A, et al. Effects of Steroid Iontophoresis and Electrotherapy on Bicipital Tendonitis. J Musculoskelet Pain 2007;15:47–54.	Insufficient exercise data
Tetschke E, Rudolf M, Lohmann CH, et al. Autologous proliferative therapies in recalcitrant lateral epicondylitis. Am J Phys Med Rehabil 2015;1:696-706.	Insufficient exercise data
Thanasas C, Papadimitriou G, Charalambidis C, et al. Platelet-rich plasma versus autologous whole blood for the treatment of chronic lateral elbow epicondylitis: a randomized controlled clinical trial. Am J Sports Med 2011;39:2130–4.	Insufficient exercise data
Thompson G, Pearson JF. No attributable effects of PRP on greater trochanteric pain syndrome. N Z Med J 2019;132:22–32.	Insufficient exercise data
Tonks JH. Evaluation of short-term conservative treatment in patients with tennis elbow (lateral epicondylitis): A prospective randomised, assessor-blinded trial (Doctoral dissertation, University of Central Lancashire).	Insufficient exercise data
Turgut E, Duzgun I. AB1428-HPR Two-year follow-up of the therapeutic exercise program for patients with rotator cuff tendinopathy: a single group study to investigate the effects on pain and disability. In: Saturday, 16 JUNE 2018. BMJ Publishing Group Ltd and European League Against Rheumatism 2018. 1847.3-1848.	Insufficient exercise data
van der Plas A, de Jonge S, de Vos RJ, et al. A 5-year follow-up study of Alfredson's heel-drop exercise programme in chronic midportion Achilles tendinopathy. Br J Sport Med 2012;46:214–8.	Insufficient exercise data
van der Vlist AC, Veldhoven PLJ, Oosterom RF, et al. Isometric exercises do not provide immediate pain relief in Achilles tendinopathy: A quasi-randomized clinical trial. Scand J Med Sci Sports 2020;30:1712–21.	Insufficient exercise data
van der Worp H, Zwerver J, Hamstra M, et al. No difference in effectiveness between focused and radial shockwave therapy for treating patellar tendinopathy: a randomized controlled trial. Knee Surgery, Sport Traumatol Arthrosc 2014;22:2026–32.	Insufficient exercise data

Wang CJ, Ko JY, Chan YS, et al. Extracorporeal shockwave for chronic patellar tendinopathy. Am J Sports Med 2007;35.	Insufficient exercise data
Weir A, Jansen J, Van de Port IGL, et al. Manual or exercise therapy for long-standing adductor-related groin pain: a randomised controlled clinical trial. Man Ther 2011;16:148–54.	Insufficient exercise data
Wiener M, Mayer F. Effects of physiotherapy on peak torque and pain in patients with tendinitis of the supraspinatus muscle. Dtsch Z Sportmed 2005;56:383–7.	Insufficient exercise data
Wilson JK, Sevier TL, Helfst R, et al. Comparison of rehabilitation methods in the treatment of patellar tendinitis. J Sport Rehabil 2000;9:304–14.	Insufficient exercise data
Worsley P, Mottram S, Warner M, et al. Clinical outcomes following motor control rehabilitation for shoulder impingement. Rheumatology 2012;51:95.	Insufficient exercise data
Yelland M, Rabago D, Ryan M, et al. Prolotherapy injections and physiotherapy used singly and in combination for lateral epicondylalgia: a single-blinded randomised clinical trial. BMC Musculoskelet Disord 2019;20:509.	Insufficient exercise data
Yerlikaya M, Talay Çaliş H, Tomruk Sütbeyaz S, et al. Comparison of Effects of Leukocyte-Rich and Leukocyte-Poor Platelet-Rich Plasma on Pain and Functionality in Patients With Lateral Epicondylitis. Archives of rheumatology 2017;33:73-79	Insufficient exercise data
Yildirim MA, Ones K, Celik EC. Comparison of ultrasound therapy of various durations in the treatment of subacromial impingement syndrome. J Phys Ther Sci 2013;25:1151–4.	Insufficient exercise data
Young M, Cook J, Purdam C, et al. Conservative treatment of patellar tendinopathy: A randomised trial comparing two treatment regimes [Abstract]. J Sci Med Sport 2002;5:120.	Insufficient exercise data
Yuksel E, Yesilyaprak SS. The Effectiveness of Scapular Stabilization Exercises in Patients with Subacromial Impingement Syndrome and Scapular Dyskinesis. Ann Rheum Dis 2015;74:1316.	Insufficient exercise data
Abat F, Sánchez-Sánchez JL, Martín-Nogueras AM, Calvo-Arenillas JI, Yajeya J, Méndez-Sánchez R, Monllau JC, Gelber PE. Randomized controlled trial comparing the effectiveness of the ultrasound-guided galvanic electrolysis technique (USGET) versus conventional electro-physiotherapeutic treatment on patellar tendinopathy. Journal of experimental orthopaedics. 2016 Dec;3(1):1-8.	Not including exercise only treatment arm
Aktas I, Akgun K, Cakmak B. Therapeutic effect of pulsed electromagnetic field in conservative treatment of subacromial impingement syndrome. Clinical rheumatology. 2007 Aug;26(8):1234-9.	Not including exercise only treatment arm
Akyol Y, Ulus Y, Durmus D, Canturk F, Bilgici A, Kuru O, Bek Y. Effectiveness of microwave diathermy on pain, functional capacity, muscle strength, quality of life, and depression in patients with subacromial impingement syndrome: a randomized placebo-controlled clinical study. Rheumatology international. 2012 Oct;32(10):3007-16.	Not including exercise only treatment arm
Bağcıer F, Yılmaz N. The Impact of Extracorporeal Shock Wave Therapy and Dry Needling Combination on the Pain, Grip Strength and Functionality in Patients Diagnosed with Lateral Epicondylitis.	Not including exercise only treatment arm

Turkish Journal of Osteoporosis/Turk Osteoporoz Dergisi. 2019 Aug 1;25(2).	
Beaudreuil J, Lasbleiz S, Yelnik A, Bardin T, Orcel P. Effect of dynamic humeral centering on painful active elevation of the arm in subacromial impingement syndrome: A randomized trial. Annals of Physical and Rehabilitation Medicine. 2012(55):e161.	Not including exercise only treatment arm
Bell KJ, Fulcher ML, Rowlands DS, Kerse N. Impact of autologous blood injections in treatment of mid-portion Achilles tendinopathy: double blind randomised controlled trial. Bmj. 2013 Apr 18;346.	Not including exercise only treatment arm
Belley AF, Mercier C, Bastien M, Léonard G, Gaudreault N, Roy JS. Anodal transcranial direct-current stimulation to enhance rehabilitation in individuals with rotator cuff tendinopathy: a triple-blind randomized controlled trial. journal of orthopaedic & sports physical therapy. 2018 Jul;48(7):541-51.	Not including exercise only treatment arm
Bennell K, Wee E, Coburn S, Green S, Harris A, Staples M, Forbes A, Buchbinder R. Efficacy of standardised manual therapy and home exercise programme for chronic rotator cuff disease: randomised placebo controlled trial. Bmj. 2010 Jun 8;340.	Not including exercise only treatment arm
Boesen AP, Hansen R, Boesen MI, Malliaras P, Langberg H. Effect of high-volume injection, platelet-rich plasma, and sham treatment in chronic midportion Achilles tendinopathy: a randomized double-blinded prospective study. The American journal of sports medicine. 2017 Jul;45(9):2034-43.	Not including exercise only treatment arm
Boesen AP, Langberg H, Hansen R, Malliaras P, Boesen MI. High volume injection with and without corticosteroid in chronic midportion achilles tendinopathy. Scandinavian Journal of Medicine & Science in Sports. 2019 Aug;29(8):1223-31.	Not including exercise only treatment arm
Buyuksireci DE, Turk AC. Evaluation of the effectiveness of dexamethasone iontophoresis in patients with subacromial impingement syndrome. Journal of Orthopaedic Science. 2021 Sep 1;26(5):786-91.	Not including exercise only treatment arm
Cacchio A, Rompe JD, Furia JP, Susi P, Santilli V, De Paulis F. Shockwave therapy for the treatment of chronic proximal hamstring tendinopathy in professional athletes. The American journal of sports medicine. 2011 Jan;39(1):146-53.	Not including exercise only treatment arm
Carey TS. Corticosteroid injection worsened recovery and recurrence of tennis elbow; physiotherapy had no effect. Annals of Internal Medicine. 2013 May;158(10):JC8.	Not including exercise only treatment arm
Celik D, Akyuz G, Yeldan I. Comparison of the effects of two different exercise programs on pain in subacromial impingement syndrome. Acta Orthop Traumatol Turc. 2009 Jan 1;43(6):504-9.	Not including exercise only treatment arm
Celik D, Anaforoglu Kulunkoglu B. Photobiomodulation therapy versus extracorporeal shock wave therapy in the treatment of lateral epicondylitis. Photobiomodulation, photomedicine, and laser surgery. 2019 May 1;37(5):269-75.	Not including exercise only treatment arm
Celik D, Atalar AC, Guclu A, Demirhan M. The contribution of subacromial injection to the conservative treatment of impingement syndrome. Acta Orthop Traumatol Turc. 2009 Aug 1;43(4):331-5.	Not including exercise only treatment arm

Cha JY, Kim JH, Hong J, Choi YT, Kim MH, Cho JH, Ko IG, Jee YS. A 12-week rehabilitation program improves body composition, pain sensation, and internal/external torques of baseball pitchers with shoulder impingement symptom. Journal of exercise rehabilitation. 2014 Feb;10(1):35.	Not including exercise only treatment arm
Chary-Valckenaere I, Loeuille D, Jay N, Kohler F, Tamisier JN, Roques CF, Boulange M, Gay G. Spa therapy together with supervised self-mobilisation improves pain, function and quality of life in patients with chronic shoulder pain: a single-blind randomised controlled trial. International journal of biometeorology. 2018 Jun;62(6):1003-14.	Not including exercise only treatment arm
Chen TW, Huei Su J, Lin TY, Lin CW, Chou PS. Effects of eccentric exercise and extracorporeal shock wave therapy on rehabilitation of patients with noncalcific rotator cuff tendinopathy. Clin Res Foot Ankle. 2017;5(222):2.	Not including exercise only treatment arm
Chung B, Wiley JP. Effectiveness of extracorporeal shock wave therapy in the treatment of previously untreated lateral epicondylitis: a randomized controlled trial. The American journal of sports medicine. 2004 Oct;32(7):1660-7.	Not including exercise only treatment arm
Clarke AW, Alyas F, Morris T, Robertson CJ, Bell J, Connell DA. Skin-derived tenocyte-like cells for the treatment of patellar tendinopathy. The American journal of sports medicine. 2011 Mar;39(3):614-23.	Not including exercise only treatment arm
Conroy DE, Hayes KW. The effect of joint mobilization as a component of comprehensive treatment for primary shoulder impingement syndrome. Journal of Orthopaedic & Sports Physical Therapy. 1998 Jul;28(1):3-14.	Not including exercise only treatment arm
Croisier JL, Foidart-Dessalle M, Tinant F, Crielaard JM, Forthomme B. An isokinetic eccentric programme for the management of chronic lateral epicondylar tendinopathy. British journal of sports medicine. 2007 Apr 1;41(4):269-75.	Not including exercise only treatment arm
de Miguel Valtierra L, Moreno JS, Fernández-de-Las-Peñas C, Cleland JA, Arias-Buría JL. Ultrasound-guided application of percutaneous electrolysis as an adjunct to exercise and manual therapy for subacromial pain syndrome: A randomized clinical trial. The Journal of Pain. 2018 Oct 1;19(10):1201-10.	Not including exercise only treatment arm
De Vos RJ, Weir A, van Schie HT, Bierma-Zeinstra SM, Verhaar JA, Weinans H, Tol JL. Platelet-rich plasma injection for chronic Achilles tendinopathy: a randomized controlled trial. Jama. 2010 Jan 13;303(2):144-9.	Not including exercise only treatment arm
Dogan SK, Saime AY, Evcik D. The effectiveness of low laser therapy in subacromial impingement syndrome: a randomized placebo controlled double-blind prospective study. Clinics. 2010 Jan 1;65(10):1019-22.	Not including exercise only treatment arm
EKEN GEDİK D, DOST SÜRÜCÜ G, YILDIRIM A, KARABİBER M. LATERAL EPİKONDİLİT TEDAVİSİNDE OTOLOG KAN ENJEKSİYONUNUN ETKİNLİĞİ: RANDOMİZE KLİNİK ÇALIŞMA. Duzce Medical Journal. 2016 Jan 1;18(1).	Not including exercise only treatment arm

Ellegaard K, Christensen R, Rosager S, Bartholdy C, Torp-Pedersen S, Bandholm T, Danneskiold-Samsøe B, Bliddal H, Henriksen M. Exercise therapy after ultrasound-guided corticosteroid injections in patients with subacromial pain syndrome: a randomized controlled trial. Arthritis research & therapy. 2016 Dec;18(1):1-9.	Not including exercise only treatment arm
Eraslan L, Yuce D, Erbilici A, Baltaci G. Does Kinesiotaping improve pain and functionality in patients with newly diagnosed lateral epicondylitis?. Knee Surgery, Sports Traumatology, Arthroscopy. 2018 Mar;26(3):938-45.	Not including exercise only treatment arm
Faria AP, da Silva EB, Dantas EH. Os efeitos dos diferentes recursos fototerapêuticos sobre a dor em indivíduos portadores de síndrome do impacto do ombro. Fitness & performance journal. 2006(6):354-8.	Not including exercise only treatment arm
García I, Lobo C, López E, Serván JL, Tenías JM. Comparative effectiveness of ultrasonophoresis and iontophoresis in impingement syndrome: a double-blind, randomized, placebo controlled trial. Clinical rehabilitation. 2016 Apr;30(4):347-58.	Not including exercise only treatment arm
Gedrimė DJ, Gedrimas D, Karpavičienė A, Skurvydas A. Effect of Visual and Auditory Feedback Exercises on Shoulder Function in Rotator Cuff Tendonitis Patients. Baltic Journal of Sport and Health Sciences. 2018 Jun 25;2(109).	Not including exercise only treatment arm
Gursel YK, Ulus Y, Bilgic A, Dincer G, van der Heijden GJ. Adding ultrasound in the management of soft tissue disorders of the shoulder: a randomized placebo-controlled trial. Physical therapy. 2004 Apr 1;84(4):336-43.	Not including exercise only treatment arm
Holmgren T, Hallgren HB, Öberg B, Adolfsson L, Johansson K. Effect of specific exercise strategy on need for surgery in patients with subacromial impingement syndrome: randomised controlled study. Bmj. 2012 Feb 20;344.	Not including exercise only treatment arm
Houck J, Neville C, Tome J, Flemister A. Randomized controlled trial comparing orthosis augmented by either stretching or stretching and strengthening for stage II tibialis posterior tendon dysfunction. Foot & ankle international. 2015 Sep;36(9):1006-16.	Not including exercise only treatment arm
Ilhanli I, Guder N, Gul M. Platelet-rich plasma treatment with physical therapy in chronic partial supraspinatus tears. Iranian Red Crescent Medical Journal. 2015 Sep;17(9).	Not including exercise only treatment arm
Ingwersen KG, Jensen SL, Sørensen L, Jørgensen HR, Christensen R, Søgaard K, Juul-Kristensen B. Three months of progressive high-load versus traditional low-load strength training among patients with rotator cuff tendinopathy: primary results from the double-blind randomized controlled RoCTEx trial. Orthopaedic journal of sports medicine. 2017 Aug 23;5(8):2325967117723292.	Not including exercise only treatment arm
Kachanathu SJ, Alenazi AM, Hafez AR, Algarni AD, Alsubiheen AM. Comparison of the effects of short-duration wrist joint splinting combined with physical therapy and physical therapy alone on the management of patients with lateral epicondylitis. European journal of physical and rehabilitation medicine. 2019 Mar 21;55(4):488-93.	Not including exercise only treatment arm
Kang FJ, Chiu YC, Wu SC, Wang TG, Yang JL, Lin JJ. Kinesiology taping with exercise does not provide additional improvement in round shoulder subjects with impingement syndrome: a single-blinded randomized controlled trial. Physical Therapy in Sport. 2019 Nov 1;40:99-106.	Not including exercise only treatment arm

Kedia M, Williams M, Jain L, Barron M, Bird N, Blackwell B, Richardson DR, Ishikawa S, Murphy GA. The effects of conventional physical therapy and eccentric strengthening for insertional achilles tendinopathy. International journal of sports physical therapy. 2014 Aug;9(4):488.	Not including exercise only treatment arm
Koç C, Kurt EE, Koçak FA, Erdem HR, Konar NM. Does balneotherapy provide additive effects to physical therapy in patients with subacute supraspinatus tendinopathy? A randomized, controlled, single-blind study. International Journal of Biometeorology. 2021 Feb;65(2):301-10.	Not including exercise only treatment arm
Kulig K, Reischl SF, Pomrantz AB, Burnfield JM, Mais-Requejo S, Thordarson DB, Smith RW. Nonsurgical management of posterior tibial tendon dysfunction with orthoses and resistive exercise: a randomized controlled trial. Physical Therapy. 2009 Jan 1;89(1):26-37.	Not including exercise only treatment arm
Land H, Gordon S, Watt K. Effect of manual physiotherapy in homogeneous individuals with subacromial shoulder impingement: a randomized controlled trial. Physiotherapy research international. 2019 Apr;24(2):e1768.	Not including exercise only treatment arm
Lee S, Ko Y, Lee W. Changes in pain, dysfunction, and grip strength of patients with acute lateral epicondylitis caused by frequency of physical therapy: a randomized controlled trial. Journal of physical therapy science. 2014;26(7):1037-40.	Not including exercise only treatment arm
Lee WC, Ng GY, Zhang ZJ, Malliaras P, Masci L, Fu SN. Changes on tendon stiffness and clinical outcomes in athletes are associated with patellar tendinopathy after eccentric exercise. Clinical Journal of Sport Medicine. 2020 Jan 1;30(1):25-32.	Not including exercise only treatment arm
Lee WC. The mechanical, physiological and therapeutic effects of eccentric exercise combined with extracorporeal shockwave therapy in athletes with patellar tendinopathy. 2017.	Not including exercise only treatment arm
Mayer F, Hirschmüller A, Müller S, Schuberth M, Baur H. Effects of short-term treatment strategies over 4 weeks in Achilles tendinopathy. British journal of sports medicine. 2007 Jul 1;41(7):e6-.	Not including exercise only treatment arm
McGee C, Kersting E, Palmer-McLean K, Davies GJ. Standard rehabilitation vs standard plus closed kinetic chain rehabilitation for patients with shoulder impingement: A rehabilitation outcomes study. Physical Therapy. 1999;79.	Not including exercise only treatment arm
McQueen KS, Powell RK, Keener T, Whalley R, Calfee RP. Role of strengthening during nonoperative treatment of lateral epicondyle tendinopathy. Journal of Hand Therapy. 2021 Oct 1;34(4):619-26.	Not including exercise only treatment arm
Menek B, Algun ZC, Tarakçı D. Effectiveness of mulligan mobilization on range of motion and function in individuals with subacromial impingement syndrome. InWCO-IOF-ESCEO World Congress on Osteoporosis, Osteoarthritis and Musculoskeletal Diseases 2018. Springer London Ltd.	Not including exercise only treatment arm
Nazligul T, Akpinar P, Aktas I, Hartevioglu C. The effect of interferential current therapy on patients with subacromial impingement syndrome: a randomized, double-blind, sham-controlled study. European Journal of Physical and Rehabilitation Medicine. 2017 Sep 11;54(3):351-7.	Not including exercise only treatment arm

Nilgun BE, Simsek I, Suat ER, Yakut Y, Uygur F. Home-based general versus center-based selective rehabilitation in patients with posterior tibial tendon dysfunction. Acta orthopaedica et traumatologica turcica. 2012 Jan 1;46(4):286-92.	Not including exercise only treatment arm
Notarnicola A, Maccagnano G, Tafuri S, Forcignanò MI, Panella A, Moretti B. CHELT therapy in the treatment of chronic insertional Achilles tendinopathy. Lasers in Medical Science. 2014 May;29(3):1217-25.	Not including exercise only treatment arm
Olaussen M, Holmedal Ø, Mdala I, Brage S, Lindbæk M. Corticosteroid or placebo injection combined with deep transverse friction massage, Mills manipulation, stretching and eccentric exercise for acute lateral epicondylitis: a randomised, controlled trial. BMC músculoskeletal disorders. 2015 Dec;16(1):1-3.	Not including exercise only treatment arm
Pekgöz F, Taşkıran H, Mutlu EK, Atalay A, Çeliker R. Comparison of mobilization with supervised exercise for patients with subacromial impingement syndrome. Turkish journal of physical medicine and rehabilitation. 2020 Jun;66(2):184.	Not including exercise only treatment arm
Pérez-Merino L, Casajuana MC, Bernal G, Faba J, Astilleros AE, González R, Giralt M, Romeu M, Nogués MR. Evaluation of the effectiveness of three physiotherapeutic treatments for subacromial impingement syndrome: a randomised clinical trial. Physiotherapy. 2016 Mar 1;102(1):57-63.	Not including exercise only treatment arm
Ramon S, Russo S, Santoboni F, Lucenteforte G, Di Luise C, de Unzurrunzaga R, Vetrano M, Albano M, Baldini R, Cugat R, Stella G. Focused shockwave treatment for greater trochanteric pain syndrome: a multicenter, randomized, controlled clinical trial. JBJS. 2020 Aug 5;102(15):1305-11.	Not including exercise only treatment arm
Rhon DI, Boyles RB, Cleland JA. One-year outcome of subacromial corticosteroid injection compared with manual physical therapy for the management of the unilateral shoulder impingement syndrome: a pragmatic randomized trial. Annals of internal medicine. 2014 Aug 5;161(3):161-9.	Not including exercise only treatment arm
Rodríguez-Huguet M, Góngora-Rodríguez J, Lomas-Vega R, Martín-Valero R, Díaz-Fernández Á, Obrero-Gaitán E, Ibáñez-Vera AJ, Rodríguez-Almagro D. Percutaneous electrolysis in the treatment of lateral epicondylalgia: A single-blind randomized controlled trial. Journal of Clinical Medicine. 2020 Jul 1;9(7):2068.	Not including exercise only treatment arm
Rodríguez-Huguet M, Góngora-Rodríguez J, Rodríguez-Huguet P, Ibañez-Vera AJ, Rodríguez-Almagro D, Martín-Valero R, Díaz-Fernández Á, Lomas-Vega R. Effectiveness of percutaneous electrolysis in supraspinatus tendinopathy: A single-blinded randomized controlled trial. Journal of Clinical Medicine. 2020 Jun 12;9(6):1837.	Not including exercise only treatment arm
Røe C, Ødegaard TT, Hilde F, Maehlum S, Halvorsen T. No effect of supplement of essential fatty acids on lateral epicondylitis. Tidsskrift for den Norske Laegeforening: Tidsskrift for Praktisk Medicin, ny Raekke. 2005 Oct 1;125(19):2615-8.	Not including exercise only treatment arm
Rosety-Rodríguez M, Ordóñez-Muñoz FJ, Huesa-Jiménez F, Rosety Rodriguez J, Gómez-Rodríguez F, Rosety-Plaza M. Actualización del trabajo excéntrico de cuádriceps en pacientes en edad laboral con	Not including exercise only treatment arm

tendinopatía rotuliana. Patología del aparato locomotor. 2006 Jun;4(2):105-7.	
Santamato A, Panza F, Notarnicola A, Cassatella G, Fortunato F, De Sanctis JL, Valeno G, Kehoe PG, Seripa D, Logroscino G, Fiore P. Is extracorporeal shockwave therapy combined with isokinetic exercise more effective than extracorporeal shockwave therapy alone for subacromial impingement syndrome? A randomized clinical trial. Journal of Orthopaedic & Sports Physical Therapy. 2016 Sep;46(9):714-25.	Not including exercise only treatment arm
Scott A, LaPrade RF, Harmon KG, Filardo G, Kon E, Della Villa S, Bahr R, Moksnes H, Torgalsen T, Lee J, Dragoo JL. Platelet-rich plasma for patellar tendinopathy: a randomized controlled trial of leukocyte-rich PRP or leukocyte-poor PRP versus saline. The American journal of sports medicine. 2019 Jun;47(7):1654-61.	Not including exercise only treatment arm
Smidt N, Van Der Windt DA, Assendelft WJ, Devillé WL, Korthals-de Bos IB, Bouter LM. Corticosteroid injections, physiotherapy, or a wait-and-see policy for lateral epicondylitis: a randomised controlled trial. The Lancet. 2002 Feb 23;359(9307):657-62.	Not including exercise only treatment arm
Söderberg J, Grooten WJ, Äng BO. Effects of eccentric training on hand strength in subjects with lateral epicondylalgia: a randomized-controlled trial. Scandinavian journal of medicine & science in sports. 2012 Dec;22(6):797-803.	Not including exercise only treatment arm
Stasinopoulos D, Stasinopoulos I, Pantelis M, Stasinopoulou K. Comparing the effects of exercise program and low-level laser therapy with exercise program and polarized polychromatic non-coherent light (bioptron light) on the treatment of lateral elbow tendinopathy. Photomedicine and laser surgery. 2009 Jun 1;27(3):513-20.	Not including exercise only treatment arm
Stergioulas A, Stergioula M, Aarskog R, Lopes-Martins RA, Bjordal JM. Effects of low-level laser therapy and eccentric exercises in the treatment of recreational athletes with chronic achilles tendinopathy. The American journal of sports medicine. 2008 May;36(5):881-7.	Not including exercise only treatment arm
Stergioulas A. Effects of low-level laser and plyometric exercises in the treatment of lateral epicondylitis. Photomedicine and laser surgery. 2007 Jun 1;25(3):205-13.	Not including exercise only treatment arm
Struijs PA, Kerkhoffs GM, Assendelft WJ, van Dijk CN. Conservative treatment of lateral epicondylitis: brace versus physical therapy or a combination of both—a randomized clinical trial. The American journal of sports medicine. 2004 Mar;32(2):462-9.	Not including exercise only treatment arm
Thijs KM, Zwerver J, Backx FJ, Steeneken V, Rayer S, Groenenboom P, Moen MH. Effectiveness of shockwave treatment combined with eccentric training for patellar tendinopathy: a double-blinded randomized study. Clinical journal of sport medicine. 2017 Mar 1;27(2):89-96.	Not including exercise only treatment arm
Tumilty S, Mani R, Baxter GD. Photobiomodulation and eccentric exercise for Achilles tendinopathy: a randomized controlled trial. Lasers in medical science. 2016 Jan;31(1):127-35.	Not including exercise only treatment arm
Tumilty S, McDonough S, Hurley DA, Baxter GD. Clinical effectiveness of low-level laser therapy as an adjunct to eccentric exercise for the treatment of Achilles' tendinopathy: a randomized	Not including exercise only treatment arm

controlled trial. Archives of physical medicine and rehabilitation. 2012 May 1;93(5):733-9.	
Tyler TF, Thomas GC, Nicholas SJ, McHugh MP. Addition of isolated wrist extensor eccentric exercise to standard treatment for chronic lateral epicondylosis: a prospective randomized trial. Journal of Shoulder and Elbow surgery. 2010 Sep 1;19(6):917-22.	Not including exercise only treatment arm
van der Vlist AC, van Oosterom RF, van Veldhoven PL, Bierma-Zeinstra SM, Waarsing JH, Verhaar JA, de Vos RJ. Effectiveness of a high volume injection as treatment for chronic Achilles tendinopathy: randomised controlled trial. bmj. 2020 Sep 9;370.	Not including exercise only treatment arm
Warden SJ, Metcalf BR, Kiss ZS, Cook JL, Purdam CR, Bennell KL, Crossley KM. Low-intensity pulsed ultrasound for chronic patellar tendinopathy: a randomized, double-blind, placebo-controlled trial. Rheumatology. 2008 Apr 1;47(4):467-71.	Not including exercise only treatment arm
Yazmalar L, Sarıyıldız MA, Batmaz İ, Alpaycı M, Burkan YK, Özkan Y, Okçu M, Çevik R. Efficiency of therapeutic ultrasound on pain, disability, anxiety, depression, sleep and quality of life in patients with subacromial impingement syndrome: A randomized controlled study. Journal of Back and Musculoskeletal Rehabilitation. 2016 Jan 1;29(4):801-7.	Not including exercise only treatment arm
Yeldan I, Cetin E, Razak Ozdincler A. The effectiveness of low-level laser therapy on shoulder function in subacromial impingement syndrome. Disability and rehabilitation. 2009 Jan 1;31(11):935-40.	Not including exercise only treatment arm
Yuruk ZO, Kirdi N, editors. The effects of radial extracorporeal shock wave therapy on subjective pain and functionality in patients with lateral epicondylitis. Fizyoterapi Rehabilitasyon Conference: 15th Congress of Advances in Physiotherapy; 2014; Ankara Turkey: Turkish Physical Therapy Association.	Not including exercise only treatment arm
Askling CM, Tengvar M, Tarassova O, Thorstensson A. Acute hamstring injuries in Swedish elite sprinters and jumpers: a prospective randomised controlled clinical trial comparing two rehabilitation protocols. British journal of sports medicine. 2014 Apr 1;48(7):532-9.	Not including required tendinopathies
Genç E, Duymaz T. Effectiveness of kinesio taping in bicipital tendinitis treatment: A randomized controlled trial. Annals of Clinical and Analytical Medicine. 2020.	Not including required tendinopathies
Jeong TH, Oh JK, Lee HJ, Yang YJ, Nha KW, Suh JS. The effect of the combined stretching and strengthening exercise on the clinical symptoms in posterior tibial tendon dysfunction patient. Journal of Korean Foot and Ankle Society. 2008;12(1):47-54.	Not including required tendinopathies
Bialoszewski D, Zaborowski G. Usefulness of Manual Therapy in the Rehabilitation of Patients with Chronic Rotator Cuff Injuries. Preliminary Report. Ortop Traumatol Rehabil 2011;1:9-20.	Not tendinopathy specific
Winters JC, Sobel JS, Groenier KH, et al. Comparison of physiotherapy, manipulation, and corticosteroid injection for treating shoulder complaints in general practice: randomised, single blind study. BMJ 1997;3:1320-1325.	Not tendinopathy specific
Alfredson H, Öhberg L. Sclerosing injections to areas of neo-vascularisation reduce pain in chronic Achilles tendinopathy: a	Wrong concept

double-blind randomised controlled trial. Knee Surg Sports Traumatol Arthrosc 2005; 13:338-44.	
Brinks A, van Rijn RM, Willemsen SP, Bohnen AM, Verhaar JA, Koes BW, Bierma-Zeinstra SM. Corticosteroid injections for greater trochanteric pain syndrome: a randomized controlled trial in primary care. Ann Fam Med 2011;1:226-34.	Wrong concept
Ebbesen BH, Mølgaard CM, Olesen JL, et al. No beneficial effect of polidocanol treatment in achilles tendinopathy: a randomised controlled trial. Knee Surg Sports Traumatol Arthrosc 2018;26:2038-44.	Wrong concept
Furia JP. Extracorporeal shockwave therapy in the treatment of chronic insertional Achilles tendinopathy: A congress report. Orthopade 2005;34:571-578.	Wrong concept
Furia JP. High-energy extracorporeal shock wave therapy as a treatment for chronic noninsertional Achilles tendinopathy. Am J Sports Med 2008;36:502-508.	Wrong concept
Gündüz R, Malas FÜ, Borman P, et al. Physical therapy, corticosteroid injection, and extracorporeal shock wave treatment in lateral epicondylitis. Clin Rheumatol 2012; 1:807-12.	Wrong concept
Hoksrud A, Öhberg L, Alfredson H, et al. Ultrasound-guided sclerosis of neovessels in painful chronic patellar tendinopathy: a randomized controlled trial. Am J Sports Med 2006;34:1738-1746.	Wrong concept
Saunders L. Laser versus ultrasound in the treatment of supraspinatus tendinosis: randomised controlled trial. Physiotherapy 2003;1:365-373.	Wrong concept
Schmitt J, Haake M, Tosch A, et al. Low-energy extracorporeal shock-wave treatment (ESWT) for tendinitis of the supraspinatus: a prospective, randomised study. J Bone Joint Surg Am 2001;83:873-6.	Wrong concept
Schmitt J, Tosch A, Hünerkopf M, et al. Extracorporeal shockwave therapy (ESWT) as therapeutic option in supraspinatus tendon syndrome? One year results of a placebo controlled study. Orthopade 2002;1:652-7.	Wrong concept
Skorupska E, Lisinski P, Samborski W. The effectiveness of the conservative versus myofascial pain physiotherapy in tennis elbow patients: double-blind randomized trial of 80 patients. J Musculoskelet Pain 2012; 1:41-50.	Wrong concept
Speed CA, Richards C, Nichols D, et al. Extracorporeal shock-wave therapy for tendonitis of the rotator cuff: a double-blind, randomised, controlled trial. J Bone Joint Surg Am 2002;84:509-12.	Wrong concept
Speed CA, Richards C, Nichols D, et al. Extracorporeal shock-wave therapy for tendonitis of the rotator cuff: a double-blind, randomised, controlled trial. J Bone Surg Joint Am 2002;84:509-512.	Wrong concept
Zwerver J, Hartgens F, Verhagen E, et al. No effect of extracorporeal shockwave therapy on patellar tendinopathy in jumping athletes during the competitive season: a randomized clinical trial. Am J Sports Med 2011;39:1191-1199.	Wrong concept
Akhtar M, Karimi H, Gilani SA, et al. Effects of routine physiotherapy with and without neuromobilization in the management of internal shoulder impingement syndrome: A randomized controlled trial. Pak J Med Sci 2020;36:596-602	Wrong HDI rank

Atya AM. Efficacy of microcurrent electrical stimulation on pain, proprioception accuracy and functional disability in subacromial impingement: RCT. Indian J Physiother Occup Ther 2012;6:15-18.	Wrong HDI rank
Babaei-Ghazani A, Shahrami B, Fallah E, et al. Continuous shortwave diathermy with exercise reduces pain and improves function in Lateral Epicondylitis more than sham diathermy: A randomized controlled trial. J Bodyw Mov Ther 2020; 1:69-76.	Wrong HDI rank
Behera P, Dhillon M, Aggarwal S, et al. Leukocyte-poor platelet-rich plasma versus bupivacaine for recalcitrant lateral epicondylar tendinopathy. J Orthop Surg 2015;23:6-10.	Wrong HDI rank
Bhardwaj P, Dhawan A. The relative efficacy of mobilization with movement versus Cyriax physiotherapy in the treatment of lateral epicondylitis. Indian J Physiother Occup Ther 2011;5:142-146.	Wrong HDI rank
Deshak S, Yeole U, Moralwar S. Effect of Functional Task Exercises on Hand Function and Grip Strength in Patients with Lateral epicondylitis. Indian J Public Health Res Dev 2020;11:927-932.	Wrong HDI rank
Eslamian F, Shakouri SK, Ghojazadeh M, et al. Effects of low-level laser therapy in combination with physiotherapy in the management of rotator cuff tendinitis. Lasers Med Sci 2012; 27:951-8.	Wrong HDI rank
Ibrahim DH, El-Gazzar NM, El-Saadany HM, et al. Ultrasound-guided injection of platelet rich plasma versus corticosteroid for treatment of rotator cuff tendinopathy: effect on shoulder pain, disability, range of motion and ultrasonographic findings. Egypt Rheumatol 2019; 41:157-161.	Wrong HDI rank
Jiang W, Zhuang J, Zhang Y, et al. The effect of platelet-rich plasma in the treatment of external humeral epicondylitis and an analysis of the influencing factors. Int J Clin Exp Med 2020;13:3866-3874.	Wrong HDI rank
Kanniappan V, Sathosh AM. To Compare the Effect of Eccentric Exercises and Isometric Exercises for Achilles Tendinitis in Skaters. Journal Lifestyle Med 2020;10:49-54.	Wrong HDI rank
Kuhkamar MMZ, Hadadnezhad M, Tazji MK. The effect of eight weeks' scapular focused training on pain, proprioception, scapular kinematics and upper extremity performance in male volleyball players with shoulder impingement syndrome: a randomized clinical trial study. Med J Tabriz Uni Med 2020;42:466-475.	Wrong HDI rank
Kumar N, Nehru A, Rajalakshmi D. Effect of taping as a component of conservative treatment for subacromial impingement syndrome. Health 2012; 26:237-41.	Wrong HDI rank
Kumar PG, Balamurugan N, Rajavel R, et al. Comparison between the effectiveness of decline squat exercise and forward lunges in athletes with patellar tendinopathy. Drug Invent Today. 2020;14:997-1000.	Wrong HDI rank
Letafatkar A, Rabiei P, Kazempour S, et al. Comparing the effects of no intervention with therapeutic exercise, and exercise with additional Kinesio tape in patients with shoulder impingement syndrome. A three-arm randomized controlled trial. Clin Rehabil 2021;35:558-567.	Wrong HDI rank
Martins da Silva L, Maciel Bello G, Chuaste Flores B, et al. Kinesio Tape In Shoulder Rotator Cuff Tendinopathy: A Randomized, Blind Clinical Trial. Muscles Ligaments Tendons J 2020; 10:364-375.	Wrong HDI rank
Martins LV, Marziale MH. Assessment of proprioceptive exercises in the treatment of rotator cuff disorders in nursing professionals: a randomized controlled clinical trial. Braz J Phys Ther 2012;16:502-9.	Wrong HDI rank

Martins LV, Marziale MHP. Assessment of proprioceptive exercises in the treatment of rotator cuff disorders in nursing professionals: a randomized controlled clinical trial. Rev Bras Fisioter 2012;16:502-509.	Wrong HDI rank
Moezy A, Sepehrifar S, Dodaran MS. The effects of scapular stabilization based exercise therapy on pain, posture, flexibility and shoulder mobility in patients with shoulder impingement syndrome: a controlled randomized clinical trial. Med J Islam Repub Iran 2014;28:87.	Wrong HDI rank
Moslehi M, Letafatkar A, Miri H. Feedback improves the scapular-focused treatment effects in patients with shoulder impingement syndrome. Knee Surg Sports Traumatol Arthrosc 2021;29:2281-2288.	Wrong HDI rank
Mostafaee N, Divandari A, Negahban H, et al. Shoulder and scapula muscle training plus conventional physiotherapy versus conventional physiotherapy only: a randomized controlled trial of patients with lateral elbow tendinopathy. Physiother Theory Pract 2020;26:1-2.	Wrong HDI rank
Ramteke S, Samal S. To Study the Effect of Rotator Cuff Exercises on Tennis Elbow. Indian J Public Health Res Dev 2020;11:610-613.	Wrong HDI rank
Shakeri H, Keshavarz R, Arab AM, et al. A randomized clinical trial of Kinesio-taping on DASH in patients with subacromial impingement syndrome. J Nov Physiother 2013;3:169.	Wrong HDI rank
Ginn K, Cohen M. Exercise therapy for shoulder pain aimed at restoring neuromuscular control: a randomized comparative clinical trial. Journal of Rehabilitation Medicine. 2005 Mar 1;37(2):115-22.	Wrong outcomes
Østerås H, Arild Torstensen T, Arntzen G, S Østerås B. A comparison of work absence periods and the associated costs for two different modes of exercise therapies for patients with longstanding subacromial pain. Journal of Medical Economics. 2008 Jan 1;11(3):371-81.	Wrong outcomes
De Reu S. The Immediate Effects of an External Rotation Exercise Program Compared with a General Exercise Program in Patients with Rotator Cuff Tendinopathy and Healthy Controls: a Randomised Controlled Trial (Doctoral dissertation, Ghent University).2018.1-41.	Wrong outcomes
Desmeules F, Minville L, Riederer B, et al. Acromio-humeral distance variation measured by ultrasonography and its association with the outcome of rehabilitation for shoulder impingement syndrome. Clin J Sport Med 2004;14:197-205.	Wrong outcomes
Gatz M, Betsch M, Tingart M, et al. Effect of a 12-week Eccentric and Isometric Training in Achilles Tendinopathy on the Gastrocnemius Muscle: an Ultrasound Shear Wave Elastography Study. Musles Ligaments Tendons J 2020; 10:92-99.	Wrong outcomes
Haahr JP, Østergaard S, Dalsgaard J, Norup K, Frost P, Lausen S, Holm EA, Andersen JH. Exercises versus arthroscopic decompression in patients with subacromial impingement: a randomised, controlled study in 90 cases with a one year follow up. Ann Rheum Dis. 2005;64:760-764.	Wrong outcomes
Hakgüder A, Tastekin N, Birtane M, Uzunca K, Zateri C, Süt N. Comparison of the Short-Term Efficacy of Physical Therapy in Subacromial Impingement Syndrome Patients with Stage I and II	Wrong outcomes

Magnetic Resonance Imaging Findings. Turk. J. Rheumatol. 2011;26(2):127-134.	
Hölmich P, Uhrskou P, Ulnits L, et al. Effectiveness of active physical training as treatment for long-standing adductor-related groin pain in athletes: randomised trial. Lancet 1999; 6:439-43.	Wrong outcomes
Kachanathu SJ, Zedan AM, Hafez AR, Alodaibi FA, Alenazi AM, Nuhmani S. Effect of shoulder stability exercises on hand grip strength in patients with shoulder impingement syndrome. Somatosensory & motor research. 2019;36:97-101.	Wrong outcomes
Öhberg L, Alfredson H. Effects on neovascularisation behind the good results with eccentric training in chronic mid-portion Achilles tendinosis? Knee Surg Sports Traumatol Arthrosc 2004;12:465-470.	Wrong outcomes
Romero-Morales C, Javier Martín-Llantino P, Calvo-Lobo C, et al. Ultrasonography effectiveness of the vibration vs cryotherapy added to an eccentric exercise protocol in patients with chronic mid-portion Achilles tendinopathy: A randomised clinical trial. Int Wound J 2019;16:542-549.	Wrong outcomes
Sayana MK, Maffulli N. Eccentric calf muscle training in non-athletic patients with Achilles tendinopathy. J Sci Med Sport. 2007;10:52-8.	Wrong outcomes
Taunton JE, Ryan MB, Wong T. ECCENTRIC-ONLY HEEL DROP TRAINING: EXAMINING A DOSE RESPONSE IN PATIENTS WITH ACHILLES TENDINOSIS. Clin J Sport Med 2004;14:382-383.	Wrong outcomes
Tumilty S, Mani R, Baxter GD. Photobiomodulation and eccentric exercise for Achilles tendinopathy: a randomized controlled trial. Lasers Med Sci 2016;31:127–135.	Wrong outcomes
Brumitt J, Hutchison MK, Kang D, et al. Blood flow restriction training for the rotator cuff: a randomized controlled trial. Int J Sports Physiol Perform 2020;19:1175-1180.	Wrong population
Yiasemides R, Halaki M, Cathers I, et al. Does passive mobilization of shoulder region joints provide additional benefit over advice and exercise alone for people who have shoulder pain and minimal movement restriction? A randomized controlled trial. Phys Ther 2011; 1:178-189.	Wrong population
De Mey K, Danneels L, Cagnie B, et al. Scapular muscle rehabilitation exercises in overhead athletes with impingement symptoms: effect of a 6-week training program on muscle recruitment and functional outcome. Am J Sports Med 2012;40:1906-1915.	Wrong study design
Abat F, Diesel WJ, Gelber PE, Polidori F, Monllau JC, Sanchez-Ibañez JM. Effectiveness of the Intratissue Percutaneous Electrolysis (EPI®) technique and isoinertial eccentric exercise in the treatment of patellar tendinopathy at two years follow-up. MLTJ. 2014;4:188-193.	Wrong study design
Abat F, Gelber PE, Polidori F, Monllau JC, Sanchez-Ibañez JM. Clinical results after ultrasound-guided intratissue percutaneous electrolysis (EPI®) and eccentric exercise in the treatment of patellar tendinopathy. Knee Surg Sports Traumatol Arthrosc. 2015;23:1046-52.	Wrong study design

Baeske R, Hall T, Silva MF. The inclusion of mobilisation with movement to a standard exercise programme for patients with rotator cuff related pain: a randomised, placebo-controlled protocol trial. BMC Musculoskelet Disord 2020;21:1-10.	Wrong study design
Bernhardsson S, Klintberg IH, Wendt GK. Evaluation of an exercise concept focusing on eccentric strength training of the rotator cuff for patients with subacromial impingement syndrome. Clin Rehabil 2011;25:69-78.	Wrong study design
Croisier JL, Forthomme B, Foidart-Dessalle M, et al. Treatment of recurrent tendinitis by isokinetic eccentric exercises. Isokinet Exerc Sci 2001;9:133-141.	Wrong study design
Davidson JH, Vandervoort A, Lessard L, et al. The effect of acupuncture versus ultrasound on pain level, grip strength and disability in individuals with lateral epicondylitis: a pilot study. Physiother Can 2001;53:195-202.	Wrong study design
Fahlström M, Jonsson P, Lorentzon R, Alfredson H. Chronic Achilles tendon pain treated with eccentric calf-muscle training. Knee Surg Sports Traumatol Arthrosc. 2003;11:327-33.	Wrong study design
Gärdin A, Movin T, Svensson L, et al. The long-term clinical and MRI results following eccentric calf muscle training in chronic Achilles tendinosis. Skeletal Radiol 2010; 39:435-442.	Wrong study design
Holden S, Lyng K, Graven-Nielsen T, et al. Isometric exercise and pain in Patellar tendinopathy: a randomized crossover trial. J Sci Med 2020;1:208-14.	Wrong study design
Kaux JF, Forthomme B, Namurois MH, et al. Description of a standardized rehabilitation program based on sub-maximal eccentric following a platelet-rich plasma infiltration for jumper's knee. Muscles Ligaments Tendons J 2014;4:85-89.	Wrong study design
Keene DJ, Soutakbar H, Hopewell S, et al. Development and implementation of the physiotherapy-led exercise interventions for the treatment of rotator cuff disorders for the 'Getting it Right: Addressing Shoulder Pain'(GRASP) trial. Physiotherapy 2020;1:252-266.	Wrong study design
Knobloch K, Schreibmueller L, Longo UG, Vogt PM. Eccentric exercises for the management of tendinopathy of the main body of the Achilles tendon with or without the AirHeel™ Brace. A randomized controlled trial. A: effects on pain and microcirculation. Disabil. Rehabil. 2008;30:1685-91.	Wrong study design
Knobloch K. Eccentric training in Achilles tendinopathy: is it harmful to tendon microcirculation?. Br. J. Sports Med.2007;41:1-5.	Wrong study design
Langberg H, Ellingsgaard H, Madsen T, et al. Eccentric rehabilitation exercise increases peritendinous type I collagen synthesis in humans with Achilles tendinosis. Scand J Med Sci Sports 2007 17:61-66.	Wrong study design
Lee DR, Kim LJ. Internal-and External-Rotation Peak Torque in Little League Baseball Players with Subacromial Impingement Syndrome: Improved by Closed Kinetic Chain Shoulder Training. J Sport Rehabil 2016;25:263-265.	Wrong study design
Littlewood C, Malliaras P, Mawson S, et al. Development of a self-managed loaded exercise programme for rotator cuff tendinopathy. Physiotherapy 2013; 1;99:358-362.	Wrong study design

Lyftogt J. Prolotherapy and Achilles tendinopathy: a prospective pilot study of an old treatment. Australas Musculoskelet Med 2005;10:17-19	Wrong study design
Macías-Hernández SI, García-Morales JR, Hernández-Díaz C, et al. Tolerance and effectiveness of eccentric vs. concentric muscle strengthening in rotator cuff partial tears and moderate to severe shoulder pain. A randomized pilot study. J Clin Orthop Trauma 2021; 1:106-112.	Wrong study design
Maffulli N, Walley G, Sayana MK, Longo UG, Denaro V. Eccentric calf muscle training in athletic patients with Achilles tendinopathy. Disabil. Rehabil. 2008;30:1677-84.	Wrong study design
Malliaras P, Cridland K, Hopmans R, et al. Internet and telerehabilitation-delivered management of rotator cuff–Related shoulder pain (INTEL trial): Randomized controlled pilot and feasibility trial. JMIR mHealth uHealth 2020;8:e24311.	Wrong study design
Miller P, Osmotherly P. Does scapula taping facilitate recovery for shoulder impingement symptoms? A pilot randomized controlled trial. J Man Manip Ther 2009; 1:6E-13E.	Wrong study design
Payne C. Clinical applications of shear wave elastography to achilles tendon imaging and the monitoring of a rehabilitation protocol for achilles tendinopathy (Doctoral dissertation, University of Brighton). 2018.109-187.	Wrong study design
Pearson SJ, Stadler S, Menz H, Morrissey D, Scott I, Munteanu S, Malliaras P. Immediate and short-term effects of short-and long-duration isometric contractions in patellar tendinopathy. Clin J Sport Med. 2020;30:335-340.	Wrong study design
Roddy E, Ogollah RO, Oppong R, Zwierska I, Datta P, Hall A, Hay E, Jackson S, Jowett S, Lewis M, Shufflebotham J. Optimising outcomes of exercise and corticosteroid injection in patients with subacromial pain (impingement) syndrome: a factorial randomised trial. Br. J. Sports Med. 2021;55:262-271.	Wrong study design
Røe C, Brox JI, Bøhmer AS, et al. Muscle activation after supervised exercises in patients with rotator tendinosis. Arch Phys Med Rehabil 2000;8:67-72.	Wrong study design
Sandford FM, Sanders TA, Wilson H, et al. A randomised controlled trial of long-chain omega-3 polyunsaturated fatty acids in the management of rotator cuff related shoulder pain. BMJ Open Sport Exerc Med 2018;4:e000414.	Wrong study design
Savoie A, Mercier C, Desmeules F, Frémont P, Roy JS. Effects of a movement training oriented rehabilitation program on symptoms, functional limitations and acromiohumeral distance in individuals with subacromial pain syndrome. Man Ther. 2015;20:703-8.	Wrong study design
Silbernagel KG, Thomeé R, Eriksson BI, et al. Full symptomatic recovery does not ensure full recovery of muscle-tendon function in patients with Achilles tendinopathy. Br J Sports Med 2007;41:276-280.	Wrong study design
Sosa C, Lorenzo A, Jimenez SL, et al. Eccentric exercise in treatment of patellar tendinopathy in high level basketball players. A randomised clinical trial [Abstract]. J Strength Cond Res 2014;28:1	Wrong study design

Stasinopoulos D, Stasinopoulos I. Comparison of effects of exercise programme, pulsed ultrasound and transverse friction in the treatment of chronic patellar tendinopathy. Clin Rehabil 2004;18:347-352.	Wrong study design
Tyler TF, Nicholas SJ, Schmitt BM, et al. Clinical outcomes of the addition of eccentrics for rehabilitation of previously failed treatments of golfers elbow. Int J Sports Phys Ther 2014;9:365-370	Wrong study design
Valera-Garrido F, Minaya-Muñoz F, Medina-Mirapeix F. Ultrasound-guided percutaneous needle electrolysis in chronic lateral epicondylitis: short-term and long-term results. Acupunct Med 2014;32(6):446-454.	Wrong study design
van Ark M, Rio E, Cook J, et al. Clinical improvements are not explained by changes in tendon structure on UTC following an exercise program for patellar tendinopathy. Am J Phys Med 2018;97:708-714.	Wrong study design
van Rensburg KJ, Atkins E. Does thoracic manipulation increase shoulder range of movement in patients with subacromial impingement syndrome? A pilot study. Int Musculoskelet Med 2012; 1:101-107.	Wrong study design
Worsley P, Warner M, Mottram S, et al. Motor control retraining exercises for shoulder impingement: effects on function, muscle activation, and biomechanics in young adults. J Shoulder Elbow Surg 2013;22:e11-9.	Wrong study design

Supplementary table 7B. Table of included studies (n=110)

Study (first author, year, country)	Design	Tendinopathy Location	Participants (number (n); sex (%female); mean (sd) age; mean (sd) symptom duration in months)	Exercise-Only Treatment arms	Dominant resistance treatment	Original Author Findings
Agregaard 2021 Denmark[1]	RCT	Patellar	N= 44<br>0%female<br>Age 28.8 (5.1)<br>Symptoms 6.9 (2.4)<br>Training status Recreational	2	2* Concentric and eccentric	There were no statistically superior effect of exercising with high (90%) compared to moderate (55%) load magnitude on the mechanical, material or morphological properties.
Agregaard 2021 Denmark[2]	RCT	Patellar	N= 44<br>0%female<br>Age 28.8 (5.1)<br>Symptoms 6.9 (2.4)<br>Training status Recreational	2	2* Concentric and eccentric	There was no superior effect of exercising with a high load magnitude (HSR) compared with a moderate load magnitude (MSR) for the clinical outcome, tendon structure, or tendon function in the treatment of patellar tendinopathy in the short term. Both HSR and MSR showed equally good, continued improvements in outcomes in the long term but did not reach normal values for healthy tendons.
Alfredson 1998 Sweden[3]	Quasi-experimental	Achillies	N= 30<br>% female 20.0<br>Age 44.0 (7.0)<br>Symptoms 25.9 (3-100)**<br>Training status Recreational	1	Eccentric only	Our treatment model with heavy-load eccentric calf muscle training has a very good short-term effect on athletes in their early forties
Alfredson 1999	Quasi-experimental	Achilles	N= 24<br>% female 14.3	1	Eccentric only	Heavy-loaded, eccentric calf-muscle training seems to be a good

Sweden[4]			Age 42.6 (9.0)<br>Symptoms 23.7 (3-100)**<br>Training status Recreational			treatment mode for chronic Achilles tendinosis.
Arias-Buría 2017 Spain[5]	RCT	RCRSP	N= 50<br>% female 26.0<br>Age 48.5 (5.5)<br>Symptoms 71.9 (21.6)<br>Training status Other	1	Concentric and eccentric	This study found that the inclusion of 2 sessions of TrP-DN into an exercise program was effective for improving shoulder pain-related disability at short-, medium-, and long-term; however, no greater improvement in shoulder pain was observed.
Arias-Buría 2015 Spain[6]	RCT	RCRSP	N= 36<br>% female 75.0<br>Age 57.5 (6.4)<br>Symptoms 10.9 (2.6)<br>Training status Other	1	Concentric and eccentric	Ultrasound-guided percutaneous electrolysis combined with eccentric exercises resulted in better short-term outcomes compared to eccentric exercises alone.
Bagcier 2021 Turkey[7]	RCT	RCRSP	N= 65<br>NR%female<br>Age 57.5 (5.2)<br>Symptoms NR<br>Training status Other	1	Concentric and eccentric	Kinesiotaping was superior to sham-KT in terms of all parameters except pain. KT was also found to be as effective as conventional exercise in all parameters. In addition, ultrasound objectively revealed that the supraspinatus tendinitis can be reduced and acromiohumeral distance can be increased.
Bahr 2006 Norway[8]	RCT	Patellar	N= 40<br>% female 12.5<br>Age 30.5 (7.9)<br>Symptoms 34 (28.7)<br>Training status Other	1	Eccentric only	No added benefit was observed for surgical treatment to eccentric strength training. Eccentric training should be offered for 12 weeks before tenotomy is considered for the treatment of patellar tendinopathy.

Balius<br>2016<br>Spain[9]	RCT	Achilles	N=37<br>% female 20.4<br>Age 41.4 (11.7)<br>Symptoms NR<br>Training status<br>Other	6	3*Eccentric only	Findings confirmed the therapeutic potential of eccentric exercise at reactive and degenerative stages of tendinopathy.<br>MCVC supplementation decreased pain more than eccentric exercise alone (reactive tendinopathy)<br>Personalized stretching regime supplemented with MCVC may be appropriate for some patients
Berg<br>2021<br>Norway[10]	RCT	RCRSP	N= 21<br>47.6%female<br>Age 48.5 (13)<br>Symptoms 43 (57.5)<br>Training status<br>Other	1	Concentric and eccentric	HIIT rotator cuff exercise seems to be a feasible intervention in subacromial pain syndrome, increasing endurance performance more than usual care alone.
Beyer<br>2015<br>Denmark[11]	RCT	Achilles	N= 58<br>% female 31.9<br>Age 48.0 (2.0)<br>Symptoms 18.1 (4.3)<br>Training status<br>Other	2	Eccentric only; Concentric and eccentric	Both traditional eccentric exercise and HSR yield positive, equally good and lasting clinical results in patients with Achilles tendinopathy. HSR is associated with greater patient satisfaction after 12 weeks but not after 52 weeks.
Blume<br>2015<br>United States[12]	RCT	RCRSP	N= 34<br>% female 58.0<br>Age 49.4 (15.6)<br>Symptoms 22.7 (24.3)<br>Training status<br>Other	2	Concentric only; Eccentric only	Both eccentric and concentric PRE programs resulted in improved function, AROM, and strength in patients with SAIS. However, no difference was found between the two exercise modes, suggesting that therapists may use exercises that utilize either exercise mode in their treatment of SAIS.
Boudreau<br>2019<br>Canada[13]	RCT	RCRSP	N= 42<br>% female 52.4<br>Age 42.9 (12.0)	2	2*Concentric and eccentric	No additional benefit was found to adding coactivation to regular rotator cuff strengthening exercises at 6-weeks.

			Symptoms 43.0 (46.6)<br>Training status Other			
Breda<br>2022<br>Netherlands[14]	RCT	Patellar	N= 76<br>23.7%female<br>Age 26.5 (3.5)<br>Symptoms NR<br>Training status Recreational	2	Concentric and eccentric; Eccentric only	Patellar tendon stiffness, assessed with shear-wave elastography, is unsuitable to use as a single predictive measurement for clinical outcome. Decreasing stiffness during the course of exercise therapy is associated with improved clinical outcome in athletes recovering from patellar tendinopathy.
Breda<br>2020<br>Netherlands[15]	RCT	Patellar	N= 76<br>% female 23.7<br>Age 24 (3.9)<br>Symptoms 98.5 (NR)<br>Training status Performance	2	Isometric; Eccentric only	In patients with patellar tendinopathy, progressive tendon-loading exercises resulted in a significantly better clinical outcome after 24 weeks than eccentric exercise therapy. Progressive tendon-loading exercises are superior to eccentric exercise therapy and are therefore recommended as initial conservative treatment for patellar tendinopathy.
Chaconas<br>2017<br>United States[16]	RCT	RCRSP	N=46<br>% female 41.7<br>Age 45.9 (17.4)<br>Symptoms 49.1 (80)<br>Training status Other	2	Eccentric only	An eccentric program targeting the external rotators was superior to a general exercise program for strength, pain, and function after six months. The findings suggest eccentric training may be efficacious to improve self-report function and strength for those with SAPS.
Cheng<br>2007	RCT	RCRSP	N=94<br>% female<br>Age 32.4 (10.2)	2	2*Concentric and eccentric	An eccentric program targeting the external rotators was superior to a general exercise program for

Hong Kong, China (SAR)[17]			Symptoms 23.4<br>Training status<br>Other			strength, pain, and function after six months. The findings suggest eccentric training may be efficacious to improve self-report function and strength for those with subacromial pain syndrome.
Cho<br>2017<br>Korea (Republic of)[18]	Quasi-experimental	Patellar	N= 30<br>% female 46.7<br>Age 33.1 (29.1)<br>Symptoms 15.1 (16.1)<br>Training status<br>Other	1	Eccentric only	A rehabilitation exercise programme was more effective at improving pain, strength and function in patellar tendinopathy that injection therapy alone.
Christiansen<br>2021<br>Denmark[19]	RCT	RCRSP	N= 208<br>54.3%female<br>Age 52.3 (12)<br>Symptoms 19 (6.3)<br>Training status<br>Other	3	Concentric and eccentric	In people with subacromial pain, group-based exercise, individually supervised exercise and home-based supervised exercise regimens have similar benefits. The home exercise intervention was associated with lowest costs.
Corum<br>2021<br>Turkey[20]	RCT	Lateral elbow/tennis elbow	N= 50<br>60%female<br>Age 43 (7.6)<br>Symptoms 12 (13.6)<br>Training status<br>Other	1	Eccentric only	The radial extracorporeal shock wave therapy seems to provide no significantly superior benefit than supervised exercises with neuromuscular inhibition at least until the three months in the treatment of LE.
de Jonge<br>2008<br>Netherlands[21]	RCT	Achilles	N= 70<br>% female NR<br>Age 44.6 (26-59) **<br>Symptoms 30.7 (2-204) **<br>Training status<br>Other	1	Eccentric only	Eccentric exercises with or without a night splint improved functional outcome at one year follow-up. At follow-up there was no significant difference in clinical outcome when a night splint was used in addition to an eccentric exercise.

de Vos 2007 Netherlands[22]	RCT	Achilles	N= 63<br>% female 41.3<br>Age 44.6 (8)<br>Symptoms 30.6 (50.6)<br>Training status Recreational	1	Eccentric only	A night splint has no added benefit to eccentric exercises in the treatment of chronic midportion Achilles tendinopathy. There was no significant difference between the two groups in VISA-A score and patient satisfaction.
Dejaco 2017 Netherlands[23]	RCT	RCRSP	N=36<br>% female 47.3<br>Age 49.5 (11.3)<br>Symptoms 19.7 (20.1)<br>Training status Other	2	Eccentric only; Concentric and eccentric	12-week-isolated eccentric training programme of the RC is beneficial for shoulder function and pain after 26 weeks in patients with RC tendinopathy. However, it is no more beneficial than a conventional exercise programme for the RC and scapular muscles.
Dimitrios 2012 Greece[24]	Quasi-experimental	Patellar	N= 60<br>% female 36.7<br>Age 47.6 (5.9)<br>Symptoms 4.5 (NR)<br>Training status Other	2	Eccentric only	Eccentric training and static stretching exercises is superior to eccentric training alone to reduce pain and improve function in patients with patellar tendinopathy at the end of the treatment and at follow-up.
Dimitrios 2013 Greece[25]	Quasi-experimental	Lateral elbow/tennis elbow	N= 60<br>36.7% female<br>Age 47.5 (5.9)<br>Symptoms NR<br>Training status Other	2	Eccentric only, Isometric	Both supervised and home exercise programmes were found to be significantly effective in reducing pain and improving functional status. A specific supervised exercise programme was superior to a specific home exercise programme in reducing pain and improving function in patients with LET at the end of the treatment and at the 3 month follow-up.
Dogan 2021 Turkey[26]	RCT	RCRSP	N= 40<br>57.5%female<br>Age 46 (7.9)<br>Symptoms 10.25 (8.4)	1	Concentric and eccentric	Our results suggest that both PT and corticosteroid injection have beneficial effects on shoulder mobility and pain relief in SIS. PT should be an alternative and

			Training status<br>Other			effective treatment method to corticosteroid injection in SIS.
Dupuis<br>2018<br>Canada[27]	RCT	RCRSP	N=43<br>% female 55.8<br>Age 33.3 (11.7)<br>Symptoms 0.9 (0.3)<br>Training status<br>Other	2	Isometric	Both groups showed statistically significant improvements on symptoms and function at 2 weeks and 6 weeks but there was no difference between the short-term effect of cryotherapy and a gradual reloading exercise programme.
Eliason<br>2021<br>Sweden[28]	RCT	RCRSP	N= 120<br>50.8%female<br>Age 44.9 (9.4)<br>Symptoms 5.2 (3.6)<br>Training status<br>Other	1	Concentric and eccentric	In patients with subacromial pain syndrome guided exercises improved shoulder function compared with no treatment. Add-on joint mobilization decreased pain in the short-term compared with exercise alone or no treatment.
Ganderton<br>2018<br>Australia[29]	RCT	Gluteal	N=90<br>%female 100<br>Age 61.83 (7.81)<br>Symptoms NR<br>Training status<br>Other	2	Concentric and eccentric	Lack of treatment effect was found with the addition of an exercise program to a comprehensive education on GTPS management. The improved outcomes of the responders in the GLoBE group indicate that there may be a subgroup of patients with a GTPS diagnosis that benefit from a GLoBE intervention program.
Gatz<br>2020<br>Germany[30]	RCT	Achilles	N= 42<br>% female 35.7<br>Age 50.0 (12.0)<br>Symptoms 27.5 (23.8)<br>Training status<br>Other	2	Eccentric only; Isometric	No additional clinical benefits of adding ISOs to a basic EE program could be found in this preliminary randomized controlled trial study over a period of 3 months. SWE was able to differentiate between insertional and midportion tendon tissue and localize reported symptoms to sublocations but this did not correlate with better clinical scores

						(VISA-A) over a 3-month follow-up period.
Giray 2019 Turkey[31]	RCT	Lateral elbow/tennis elbow	N= 30<br>% female 86.7<br>Age 44.46 (9.92)<br>Symptoms 1.69 (NR)<br>Training status Other	1	Eccentric only	Kinesiotaping in addition to exercises is more effective than sham taping and exercises alone in improving pain in daily activities and arm disability due to lateral epicondylitis.
Habets 2021 Netherlands[32]	RCT	Achilles	N= 40<br>%female<br>Age 44.9 (9)<br>Symptoms 9.4 (8.2)<br>Training status Recreational	2	Eccentric only; Concentric and eccentric	No differences in clinical effects were found between Alfredson and Silbernagel loading at up to 1-year follow-up. Both programs significantly improved clinical symptoms, and given their high adherence rates, offering either of them as a homebased program with limited supervision appears to be an effective treatment strategy for midportion AT.
Hallgren 2014 Sweden[33]	RCT	RCRSP	N= 50<br>% female 37.0<br>Age 52 (30-65)**<br>Symptoms 18 (6-186)*<br>Training status Other	2	Eccentric only	Specific exercises produced positive short-term improvements at 1-year follow-up and reduces the need for surgery. Full-thickness tear and a low CMS score appear to be predictors of poor outcome.
Hallgren 2017 Sweden[34]	RCT	RCRSP	N= 108<br>% female 34.1<br>Age 58 (NR)<br>Symptoms NR<br>Training status Other	2	Concentric and eccentric	More patients in the specific exercise group managed to avoid surgery compared to the unspecific exercise group at 5-year follow-up supporting it's prescription as an initial treatment for patients with subacromial pain.
Heron 2017	RCT	RCRSP	N= 120<br>% female 41.0<br>Age 49.9 (NR)	3	2*Concentric and eccentric	Open chain, closed chain, and range of movement exercises all seem to be effective in bringing

United Kingdom[35]			Symptoms NR<br>Training status<br>Other			about short-term changes in pain and disability in patients with rotator cuff tendinopathy.
Hopewell 2021 United Kingdom[36]	RCT	RCRSP	N= 708<br>49.3%female<br>Age 55.5 (13.1)<br>Symptoms 4.3 (0.5)<br>Training status<br>Other	2	Concentric and eccentric	Progressive exercise was not superior to a best-practice advice session with a physiotherapist. Subacromial corticosteroid injection improved shoulder pain and function, but provided only modest short-term benefit. Best-practice advice in combination with corticosteroid injection was expected to be most cost-effective, although there was substantial uncertainty
Hotta 2020 Brazil[37]	RCT	RCRSP	N=60<br>% female 70<br>Age 49 (9)<br>Symptoms 28.5 (24)<br>Training status<br>Other	2	Concentric and eccentric	The inclusion of the isolated scapular stabilization exercises, emphasizing retraction and depression of the scapula, to a progressive general periscapular strengthening protocol did not add benefits to self-reported shoulder pain and disability, muscle strength, and ROM in patients with subacromial pain syndrome.
Johansson 2005 Sweden[38]	RCT	RCRSP	N=85<br>% female 69.4<br>Age 49 (7.5)<br>Symptoms NR<br>Training status<br>Other	1	Isometric	Acupuncture was more effective than ultrasound when applied in addition to home exercises.
Jonsson 2005 Sweden[39]	RCT	Patellar	N= 15<br>% female 13.3<br>Age 24.9 (8.2)<br>Symptoms 17.5 (13.2)	2	Eccentric only; Concentric only	Eccentric, but not concentric, quadriceps training on a decline board, seems to reduce pain in jumper's knee.

			Training status<br>Performance			
Ketola<br>2009<br>Finland[40]	RCT	RCRSP	N=134<br>% female 62.9<br>Age 47.1(23.3-60.0)**<br>Symptoms 2.6 (NR)<br>Training status<br>Other	1	Concentric and eccentric	Arthroscopic acromioplasty provides no clinically important effects over a structured and supervised exercise programme alone in terms of subjective outcome or cost-effectiveness when measured at 24 months.
Knobloch<br>2008<br>Italy[41]	RCT	Achilles	N= 92<br>% female 35.0<br>Age 47.5 (11.0)<br>Symptoms NR<br>Training status<br>Recreational	1	Eccentric only	Patients with tendinopathy of the main body of the AT experienced improved clinical outcome with both management options. Although tendon microcirculation was optimized in the combined group of eccentric training and AirHeel Brace, these micro-vascular advantages do not translate into superior clinical performance when compared with eccentric training alone.
Knobloch<br>2007<br>Germany[42]	RCT	Achilles	N= 20<br>% female 45.0<br>Age 32.5 (11.0)<br>Symptoms NR<br>Training status	1	Eccentric only	An eccentric-training program performed daily over 12 weeks reduced the increased paratendinous capillary blood flow in Achilles tendinopathy by as much as 45% and decreased pain level based on a visual analog scale. Local paratendon oxygenation was preserved while paratendinous postcapillary venous filling pressures were reduced after 12 weeks of eccentric training, which appears to be beneficial from the perspective of microcirculation.
Knobloch<br>2007	RCT	Achilles	N= 118<br>% female 40	1	Eccentric only	Achilles tendon oxygen saturation is increased, and capillary venous

Germany[43]			Age 48.5 (12)<br>Symptoms NR<br>Training status<br>Other			clearance facilitated using an Achilles wrap in addition to daily 12-week eccentric training
Kongsgaard<br>2009<br>Denmark[44]	RCT	Patellar	N= 37<br>% female 0<br>Age 32.4 (8.8)<br>Symptoms 18.7 (12.3)<br>Training status<br>Recreational	2	Eccentric only; Concentric and eccentric	Corticosteroid injection has good short-term but poor long-term clinical effects, in patellar tendinopathy. Heavy-slow resistance exercise has good short- and long-term clinical effects accompanied by pathology improvement and increased collagen turnover.
Kromer<br>2014<br>Germany[45]	RCT	RCRSP	N= 90<br>% female 51.1<br>Age 51.8 (11.2)<br>Symptoms 24.1 (35.1)<br>Training status<br>Other	1	Concentric and eccentric	The use of MT including Physiotherapy provides no additional benefits and is more expensive in comparison to exercise only interventions.
Kromer<br>2013<br>Germany[46]	RCT	RCRSP	N= 90<br>% female 51.1<br>Age 51.8 (11.2)<br>Symptoms 7.8 (9.8)<br>Training status<br>Other	1	Concentric and eccentric	Individually adapted exercises were effective in the treatment of patients with shoulder impingement syndrome. Individualized manual Physiotherapy contributed only a minor amount to the improvement in pain intensity.
Littlewood<br>2016<br>United Kingdom[47]	RCT	RCRSP	N= 60<br>% female 50.3<br>Age 54.7 (NR)<br>Symptoms 14.6 (NR)<br>Training status<br>Other	1	Concentric and eccentric	Self-management programme based on a single exercise were comparable to usual Physiotherapy in the short-, mid- and long-term.

Luginbuhl<br>2008<br>Switzerland[48]	RCT	Lateral elbow/tennis elbow	N= 30<br>% female 72.7<br>Age 47 (9)<br>Symptoms 10 (11)<br>Training status Other	1	Isometric	No beneficial effect of neither the forearm support band nor the strengthening exercises could be found.
Maenhout<br>2013<br>Belgium[49]	RCT	RCRSP	N= 61<br>% female 59.0<br>Age 39.8 (13.0)<br>Symptoms NR<br>Training status Other	2	Concentric and eccentric; Eccentric only	Adding heavy load eccentric training resulted in a higher gain in isometric strength at 90 degree of scapular abduction but was not superior for decreasing pain and improving shoulder function. The addition of a limited amount of Physiotherapy sessions combined with a daily home exercise programme is highly effective in patients with impingement.
Mafi<br>2001<br>Sweden[50]	RCT	Achilles	N= 44<br>% female 45.5<br>Age 48.3 (8.8)<br>Symptoms 20.5 (3-120)**<br>Training status Other	2	Eccentric only; Concentric only	Eccentric calf muscle training showed superior results to concentric training in the treatment of chronic Achilles tendinosis based on patient satisfaction and return to activity level.
Manias<br>2006<br>United Kingdom[51]	RCT	Lateral elbow/tennis elbow	N= 40<br>% female 67.5<br>Age 42.86 (6.23)<br>Symptoms NR<br>Training status Other	2	2*Eccentric only	An exercise programme consisting of eccentric and static stretching exercises had reduced the pain in patients with lateral epicondyle tendinopathy at the end of the treatment and at the follow up whether or not ice was included.
Martinez-Silvestrini<br>2005<br>United States[52]	Quasi-experimental	Lateral elbow/tennis elbow	N= 81<br>% female 46.8<br>Age 45.5 (7.7)<br>Symptoms NR<br>Training status Other	3	Concentric only; Eccentric only	Eccentric strengthening for the wrist extensors in subjects with lateral epicondylitis demonstrated improvement at six weeks but was not statistically different from that achieved with a conservative

						program with stretching or a concentric strengthening program.
Marzetti 2014 Italy[53]	RCT	RCRSP	N= 48 % female 61.4 Age 62.1 (12.5) Symptoms NR Training status Other	2	Concentric and eccentric	Neurocognitive rehabilitation is effective in reducing pain and improving function in patients with shoulder impingement syndrome, with benefits maintained for at least 24 weeks.
McCormack 2016 United States[54]	RCT	Achilles	N= 15 % female 68.8 Age 53.6 (38-69)** Symptoms 9.9 (NR) Training status Other	1	Eccentric only	Soft tissue treatment (Astym) plus eccentric exercise was more effective than eccentric exercise alone at improving function during both short- (26 weeks) and long-term (52 weeks) follow-up periods.
Mulligan 2016 United States[55]	RCT	RCRSP	N=50 % female 65 Age 50.1 (10.7) Symptoms 7.9 (7.4) Training status Other	1	Concentric and eccentric	Patients with SAIS demonstrate improvement in pain and function with a standardized program of physical therapy regardless of group exercise sequencing.
Nørregaard 2007 Denmark[56]	RCT	Achilles	N= 35 % female 49.0 Age 42.0 (2.0)*** Symptoms 28.4 (8.8)*** Training status Other	2	Eccentric only	Symptoms gradually improved during the 1-year follow-up period and were significantly better assessed by pain and symptoms after 3 weeks and all later visits. However, no significant differences could be observed between the two groups.
Nowotny 2018 Germany[57]	RCT	Lateral elbow/tennis elbow	N= 31 % female 57 Age 46 (NR) Symptoms NR Training status Other	1	Eccentric only	The use of an elbow orthosis appears to reduce pain and improve other subjective outcome measures. However, the long-term results do not appear to be any

						greater than those received through Physiotherapy alone.
Østerås<br>2010<br>Norway[58]	RCT	RCRSP	N=61<br>% female 20.5<br>Age 43.9 (13)<br>Symptoms 40.2 (56.3)<br>Training status Other	2	2*Concentric and eccentric	In long-term subacromial pain syndrome, high dosage medical exercise therapy is superior to a conventional low dosage exercise programme
Park<br>2010<br>Korea (Republic of)[59]	RCT	Lateral elbow/tennis elbow	N=31<br>% female 61.3<br>Age 50.2 (34-63)**<br>Symptoms 6.3 (2-17)**<br>Training status NR	1	Isometric	Isometric strengthening exercises done early in the course of LE (within 4 weeks) provides a clinically significant improvement.
Pearson<br>2012<br>New Zealand[60]	RCT	Patellar	N= 40<br>% female 62.5<br>Age 50.0 (8.2)<br>Symptoms 11.0 (10.0)<br>Training status Other	1	Eccentric only	There is some evidence for small short-term symptomatic improvements with the addition of autologous blood injection to standard treatment for Achilles tendinopathy.
Pearson<br>2018<br>Australia[61]	RCT	Achilles	N= 16<br>% female 0<br>Age 28 (4.25)<br>Symptoms 34.17 (1.95)<br>Training status Performance	2	2* Isometric	Pain was significantly reduced after isometric loading on both SLDS and hop tests. Pain and quadriceps function improved over the 4 weeks. Short-duration isometric contractions are found to be as effective as longer duration contractions for relieving patellar tendon pain when total time under tension is equalized.
Pekyavas<br>2016	RCT	RCRSP	N=70<br>% female NR	1	Concentric and eccentric	HILT and MT were found to be more effective in reducing pain and

Turkey[62]			Age 47.1 (13.8)<br>Symptoms NR<br>Training status Other			disability and improving ROM in patient with SAIS.
Petersen<br>2007<br>Germany[63]	RCT	Achilles	N= 86<br>% female 40.0<br>Age 42.5 (11.1)<br>Symptoms 7.4 (2.3)<br>Training status Recreational	1	Eccentric only	The AirHeel brace is as effective as eccentric training in the treatment of chronic Achilles tendinopathy. There is no added benefit to combining both treatments.
Peterson<br>2011<br>Sweden[64]	RCT	Lateral elbow/tennis elbow	N= 81<br>% female 42<br>Age 48.25 (8.35)<br>Symptoms 23.3 (35.9)<br>Training status Other	2	Concentric and eccentric	Exercise appears to be superior to the control group in reducing pain in chronic lateral epicondylosis.
Peterson<br>2014<br>Sweden[65]	RCT	Lateral elbow/tennis elbow	N= 120<br>% female 47.5<br>Age 47.9 (8.1)<br>Symptoms NR<br>Training status Other	1	Eccentric only; Concentric only	Eccentric graded exercise reduced pain and increased muscle strength in chronic tennis elbow more effectively than concentric graded exercise at follow-up. However, there were no significant differences in function or quality of life measures between the two groups.
Praet<br>2019<br>Australia[66]	RCT	Achilles	N= 20<br>% female 35.0<br>Age 43.7 (7.9)<br>Symptoms 54 (90)<br>Training status Recreational	1	Eccentric only	Oral supplementation of specific collagen peptides may accelerate the clinical benefits of a well-structured calf-strengthening and return-to-running programme in patients with chronic Achilles tendinopathy.
Rabusin<br>2020<br>Australia[67]	RCT	Achilles	N= 100<br>% female 52.0<br>Age 45.85 (9.4)	1	Eccentric only	In adults with mid-portion Achilles tendinopathy, heel lifts were more effective than calf muscle eccentric

			Symptoms 20.25 (NR)<br>Training status<br>Other			exercise in reducing pain and improving function at 12 weeks.
Rabusin<br>2021<br>Australia[68]	RCT	Achilles	N= 100<br>52%female<br>Age 45.9 (9.4)<br>Symptoms 20.9 (6.5)<br>Training status<br>Other	1	Eccentric only	In adults with mid-portion Achilles tendinopathy, heel lifts were more effective than calf muscle eccentric exercise in reducing pain and improving function at 12 weeks. However, there is uncertainty in the estimate of effect for this outcome and patients may not experience a clinically worthwhile difference between interventions.
Rio<br>2017<br>Australia[69]	RCT	Patellar	N= 20<br>% female 10.0<br>Age 22.5 (4.7)<br>Symptoms NR<br>Training status<br>Performance	2	Concentric and eccentric; Isometric	Both isometric and isotonic contraction protocols appear efficacious for in-season athletes to reduce pain, however, isometric contractions demonstrated significantly greater immediate analgesia throughout the 4-week trial.
Romero-Morales<br>2020<br>Spain[70]	RCT	Achilles	N= 61<br>% female 26<br>Age 41.6 (8.7)<br>Symptoms 4.25 (3.5)<br>Training status<br>Other	2	Eccentric only	Authors encourage the use of vibration with respect to cryotherapy added to eccentric exercise programs in order to enhance multifidus cross-sectional area in addition to lower limb functionality in individuals who suffer from chronic non-insertional AT.
Rompe<br>2007<br>Germany[71]	RCT	Achilles	N= 75<br>% female 61.3<br>Age 48.5 (10.6)<br>Symptoms 10.8 (8.5)<br>Training status<br>Other	1	Eccentric only	At 4-month follow-up, eccentric loading and low-energy shock-wave therapy showed comparable results. The wait-and-see strategy was ineffective for the management of chronic recalcitrant Achilles tendinopathy.

Rompe 2009 Germany[72]	RCT	Achilles	N= 68 % female 55.9 Age 49.7 (9.9) Symptoms 14.5 (6.0) Training status Other	1	Concentric and eccentric	The likelihood of recovery after 4 months was higher after a combined approach of both eccentric loading and shock-wave therapy compared to eccentric loading alone.
Rompe 2009 Germany[73]	RCT	Gluteal (including GTPS)	N= 68 % female 55.9 Age 49.7 (9.9) Symptoms 14.5 (6) Training status Other	1	Eccentric only	Both corticosteroid injection and home training were significantly less successful than was shock wave therapy at 4-month follow-up. Corticosteroid injection was significantly less successful than was home training or shock wave therapy at 15-month follow-up.
Rompe 2008 Germany[74]	RCT	Achilles	N= 50 % female 60.0 Age 39.8 (11) Symptoms 25.55 (9.45) Training status Other	1	Eccentric only	Eccentric loading as applied in the present study showed inferior results to low-energy shock wave therapy as applied in patients with chronic recalcitrant tendinopathy of the insertion of the Achilles tendon at four months follow-up.
Roos 2004 Sweden[75]	RCT	Achilles	N= 44 % female 52.3 Age 45 (26-60)** Symptoms 5.5 (1-180)* Training status Recreational	1	Eccentric only	Eccentric exercises reduce pain and improve function in patients with Achilles tendinopathy.
Ruffino 2021 Argentina[76]	RCT	Patellar	N= 41 2.4%female Age 29.6 (7) Symptoms 13.4 (10.8) Training status	2	Concentric and eccentric; Isokinetic	Inertial flywheel resistance three times a week during 12 weeks resulted in similar pain and function benefit at 12 weeks compared with the heavy slow resistance training among people

			Recreational			with patellar tendinopathy. Flywheel training is another exercise option for managing people with patellar tendinopathy.
Sahbaz<br>2021<br>Turkey[77]	RCT	Lateral elbow/tennis elbow	N= 74<br>81%female<br>Age 49.7 (7.6)<br>Symptoms NR<br>Training status<br>Other	1	Eccentric only	In the treatment of chronic LE, platelet-rich plasma combined with exercise seems to be superior to exercise or extracorporeal shock wave therapy in terms of pain and functionality in chronic LE patients.
Schydlowsky<br>2022<br>Denmark[78]	RCT	RCRSP	N= 126<br>48.4%female<br>Age 61 (13.2)<br>Symptoms NR<br>Training status<br>Other	2	Concentric and eccentric	We found no significant difference between a comprehensive supervised training regimen including heavy training principles, and a home-based training program in patients with SIS.
Şenbursa<br>2011<br>Turkey[79]	RCT	RCRSP	N= 47<br>% female NR<br>Age 49.0 (9.3)<br>Symptoms NR<br>Training status<br>Other	2	2*Concentric and eccentric	Supervised exercise, supervised and MT, and home-based exercise are all effective and promising treatments for patients with subacromial impingement syndrome. The addition of an initial MT may improve outcomes with exercise.
Sevier<br>2015<br>United States[80]	RCT	Lateral elbow/tennis elbow	N= 90<br>% female 57.9<br>Age 46.95 (6.55)<br>Symptoms NR<br>Training status<br>Other	1	Eccentric only	Astym therapy is an effective treatment option for patients with LE tendinopathy, as an initial treatment, and after an eccentric exercise program has failed.
Shim<br>2007<br>Korea[81]	RCT	Lateral elbow/tennis elbow	N= 63<br>%female<br>Age 51.1 (8.5)<br>Symptoms 12.5 (21.7)<br>Training status	1	Isometric	Polydeoxyribonucleotide injections combined with EX exhibited a greater improvement in mean , Mayo elbow performance score and mean common extensor tendon depth compared to EX

			Other			only or EX combined with extracorporeal shockwave therapy for LE within the 12 weeks follow-up.
Silbernagel 2007 Sweden[82]	RCT	Achilles	N= 38 % female 47.4 Age 46.0 (8.0) Symptoms 36.2 (66.5) Training status Other	2	2*Concentric and eccentric	Our treatment protocol which gradually increases the load on the Achilles tendon and calf muscle, demonstrated significant improvements. Continuing tendon loading activity such as running and jumping with the use of a pain-monitoring model did not have any adverse effect.
Silbernagel 2001 Sweden[83]	RCT	Achilles	N= 47 % female 22.5 Age 44.0 (12.5) Symptoms 30.5 (40.7) Training status Recreational	2	2*Concentric and eccentric	The eccentric overload protocol used in the present study can be recommended for patients with chronic pain from the Achilles tendon. More patients achieved full recovery, improved pain and ROM in the Exp group compared to the control group.
Şimşek, 2013 Turkey[84]	RCT	RCRSP	N= 38 % female 65.8 Age 51.0 (18-69)** Symptoms NR Training status Other	1	Isokinetic	Findings were inconclusive and require further research.
Slider 2013 United States[85]	RCT		N=24 %female 79.2 Age 24.0 (9.0) Symptoms NR Training status Recreational	2	Isokinetic; Concentric and eccentric	In general, subjects with an acute hamstring strain injury treated with either the PATS or PRES rehabilitation program demonstrated a similar degree of muscle recovery at the time of return to sport. Despite this, there were no subjects who exhibited complete resolution of injury on MRI, and 2 of the 4 subjects who

						reinjured themselves did so within the first 2 weeks after return to sport.
Solomons 2020 Canada[86]	RCT	Achilles	N= 52<br>46%female<br>Age 48 (7)<br>Symptoms 18 (15)<br>Training status Other	3	Eccentric only	The addition of intramuscular stimulation to standard rehabilitation for Achilles tendinopathy did not result in any improvement over the expected clinical benefit achieved with exercisebased rehabilitation alone.
Stasinopoulos 2017 Cyprus[87]	RCT	Lateral elbow/tennis elbow	N= 34<br>% female 55.8<br>Age 43.7 (4.6)<br>Symptoms 6 (NR)<br>Training status Recreational	3	Eccentric only; 2*Concentric and eccentric	Eccentric training, eccentric-concentric training, and eccentric-concentric training combined with isometric contraction reduced pain and improved function at the end of the treatment and follow-up. The eccentric-concentric training combined with isometric contraction produced the largest effect at the end of the treatment and follow-up.
Stasinopoulos 2006 Greece[88]	Quasi-experimental	Lateral elbow/tennis elbow	N= 75<br>% female 38.6%<br>Age 40.3 (5.8)<br>Symptoms 5 (NR)<br>Training status Other	1	Eccentric only	Cyriax Physiotherapy, a supervised exercise programme, and polarized polychromatic non-coherent light reduced pain and improved function at the end of the treatment and at any of the follow-up time points. The supervised exercise programme produced the largest effect in the short, intermediate and long term.
Stasinopoulos 2010 Greece[89]	Quasi-experimental	Lateral elbow/tennis elbow	N= 70<br>% female 52.9<br>Age 45.1 (5.8)<br>Symptoms 5 (NR)<br>Training status NR	2	2*Eccentric only	Supervised exercise programme is superior to home exercise programme to reduce pain and improve function in patients with LET at the end of the treatment and at the follow-up.

Stasinopoulos 2013 Greece[90]	RCT	Lateral elbow/tennis elbow	N= 60<br>% female 36.7<br>Age 48.0 (5.9)<br>Symptoms 4.5 (NR)<br>Training status Other	2	Isometric; Eccentric only	A specific supervised exercise programme is superior to a specific home exercise programme in reducing pain and improving function in patients with lateral epicondyle tendinopathy at the end of the treatment and at the 3 month follow-up.
Stefansson 2019 Iceland[91]	RCT	Achilles	N= 58<br>% female 20.0<br>Age NR<br>Symptoms NR<br>Training status Other	1	Eccentric only	Similar results for pressure massage and eccentric exercise. Combining pressure massage and eccentric exercise did not improve outcomes
Steunebrink 2013 Netherlands[92]	RCT	Patellar	N= 33<br>% female 24.2<br>Age 32.9 (10)<br>Symptoms 11 (8)<br>Training status Recreational	1	Resistance	Continuous topical GTN treatment in addition to an eccentric exercise programme does not improve clinical outcome compared to placebo patches and an eccentric exercise programme in patients with chronic patellar tendinopathy.
Stevens 2014 United Kingdom[93]	RCT	Achilles	N= 28<br>% female 60.7<br>Age 48.7 (10.8)<br>Symptoms 7.4 (4.0)<br>Training status Other	2	2*Eccentric only	Performing a 6-week do-as-tolerated program of eccentric heel-drop exercises compared to the recommended 180 repetitions per day, did not lead to lesser improvement for individuals with midportion Achilles tendinopathy, based on VISA-A and VAS scores.
Svernlov 2001 Sweden[94]	Quasi-experimental	Lateral elbow/tennis elbow	N= 57<br>% female 61.3<br>Age 50.15 (NR)<br>Symptoms 6.3 (NR)<br>Training status Other	1	Eccentric only	Significant improvements observed for VAS and grip strength warrants clinical use of this regime.
Tonks 2007	RCT	Lateral elbow/tennis elbow	N= 34<br>% female NR<br>Age 44.3 (7.1)	1	Isometric	Patients who received steroid injection were statistically significantly better for all outcome

United Kingdom[95]			Symptoms NR<br>Training status<br>Other			measures at follow up. No statistically significant effect of Physiotherapy nor interaction between Physiotherapy and injection was found.
Turgut<br>2017<br>Turkey[96]	RCT	RCRSP	N= 30<br>% female 46.7<br>Age 36.45 (17.5)<br>Symptoms 6.28 (5.4)<br>Training status<br>Other	2	2*Concentric and eccentric	Progressive exercise training independent from specific scapular stabilization exercises provides decreased disability and pain severity in impingement syndrome. All groups showed improvement, however, there were no significant differences between the groups.
Vallés-Carrascosa<br>2018<br>Spain[97]	RCT	RCRSP	N= 22<br>% female 54<br>Age 59.0 (58.5-70.0)*<br>Symptoms<br>Training status<br>Other	2	2*Eccentric only	Both rotator cuff eccentric exercise protocols with scapular stabilising and stretching of upper trapezius were equally effective in improving pain, function, and active ROM in the short-term in patients with subacromial syndrome.
vanArk<br>2016<br>Australia[98]	RCT	Patellar	N= 19<br>% female 6.9<br>Age 23 (4.7)<br>Symptoms 35.8 (33.8)<br>Training status<br>Recreational	2	Isometric; Concentric and eccentric	This study found favourable results for athletes with patellar tendinopathy without modification of the training. Both isometric and isotonic exercise programs reduced pain and improve function in athletes with patellar tendinopathy during a season.
Vinuesa-Montoya<br>2017<br>Spain[99]	RCT	RCRSP	N= 40<br>% female 26.8<br>Age 47.0 (9.0)<br>Symptoms 6.2 (3.8)<br>Training status<br>Other	1	Concentric and eccentric	Cervicothoracic manipulative treatment with mobilisation plus exercise therapy may improve intensity of pain and ROM compared with home exercise alone.
Visnes<br>2005<br>Norway[100]	RCT	Patellar	N= 29<br>% female 38.5<br>Age 26.58 (NR)	1	Eccentric only	There was no effect on knee function (VISA) from a 12-week program with eccentric training

			Symptoms 73.6 (62.3)<br>Training status Performance			among a group of volleyball players with patellar tendinopathy who continued to train and compete during the treatment period. Whether the training would be effective if the patients did not participate in sports activity is not known.
Vuvan<br>2019<br>Australia[101]	RCT	Lateral elbow/tennis elbow	N= 39<br>% female 28<br>Age 48.5 (9)<br>Symptoms 4 (NR)<br>Training status Other	2	Isometric	Unsupervised isometric exercise was effective in improving pain and disability, but not perceived rating of change and pain-free grip strength when compared with wait-and-see at 8 wk. With only one of the three primary outcomes being significantly improved, it is doubtful if isometric exercises can be an efficacious standalone treatment.
Walther<br>2004<br>Germany[102]	RCT	RCRSP	N= 60<br>% female 43.3<br>Age 50.7 (NR)<br>Symptoms 27.3 (NR)<br>Training status Other	2	Isometric	There were no statistically significant differences among the groups. Guided self-training can lead to results similar to those of conventional Physiotherapy.
Wegener<br>2016<br>Australia[103]	RCT	Lateral elbow/tennis elbow	N= 40<br>% female 70<br>Age 49.52 (8.09)<br>Symptoms NR<br>Training status NR	1	Eccentric only	Whilst all groups improved on key outcomes, it is possible that exercise alone and/or natural recovery were responsible for improvements.
Wen<br>2011<br>United States[104]	RCT	Lateral elbow/tennis elbow	N= 28<br>% female 46.4<br>Age 46 (7.3)<br>Symptoms 3.3 (2.2)	1	Eccentric only	The authors were unable to show any statistical advantage to eccentric exercises for lateral epicondylosis compared with local modalities and stretching exercises.

			Training status<br>Other			
Werner<br>2002<br>Germany[105]	RCT	RCRSP	N=20<br>% female 50<br>Age 51.75 (NR)<br>Symptoms 27.5<br>Training status<br>Other	2	Isometric	Strengthening of the centering muscles around the humeral head lead to positive outcomes for subacromial impingement. Self-training after instruction showed no difference to physiotherapist-supervised exercises.
Wiedmann<br>2017<br>Germany[106]	RCT	Achilles	N= 20<br>% female 65.0<br>Age 43.0 (6.0)<br>Symptoms NR<br>Training status<br>Other	1	Eccentric only	Eccentric training improved the VISA-A and VAS scores after 12 weeks more than Physiotherapy treatment.
Yelland<br>2011<br>Australia[107]	RCT	Achilles	N= 43<br>% female NR<br>Age 46.7 (NR)<br>Symptoms 17 (NR)<br>Training status<br>Other	1	Eccentric only	Prolotherapy and particularly eccentric loading exercises combined with prolotherapy gave more rapid improvements in Achilles tendinosis symptoms than eccentric loading exercises alone. Long term VISA-A scores were similar.
Yilmaz<br>2022<br>Turkey[108]	RCT	Lateral elbow/tennis elbow	N= 40<br>65%female<br>Age 42.8 (8.9)<br>Symptoms 29.9 (33.7)<br>Training status<br>Other	1	Eccentric only	Radial nerve mobilization techniques are more effective on pain than conservative rehabilitation therapy in LE patients, and this effect continues after treatment.
Young<br>2005<br>Australia[109]	RCT	Patellar	N= 17<br>% female 23.5<br>Age 27.3 (1.8)<br>Symptoms NR<br>Training status<br>Performance	2	Eccentric only; Concentric and eccentric	Both exercise protocols improved pain and sporting function in volleyball players over 12 months. The decline squat protocol offers greater clinical gains during a rehabilitation programme for

						patellar tendinopathy in athletes who continue to train and play with pain.
Yu 2013 Korea (Republic of) [110]	Quasi-experimental	Achilles	N= 32<br>% female 0.0<br>Age 30.3 (1.6)<br>Symptoms 11.7 (2.1)<br>Training status Other	2	Eccentric only; Concentric only	Eccentric strengthening was more effective than concentric strengthening in reducing pain and improving function in patients with Achilles tendinopathy.

Key: * = median (interquartile range); ** = mean (range); *** = mean (standard error of the mean); MVCV = mean dietary supplement containing mucopolysaccharides, type I collages & vitamin C; AT = Achilles Tendinopathy; GTPS = Greater trochanteric pain syndrome; F-ESWT=electromagnetic focused extracorporeal shockwave treatment; PATS = progressive agility and trunk stabilization; PRES = progressive running and eccentric strengthening; HVI = high-volume injection; PRP = platelet-rich plasma; VISA-A=Victorian Institute of Sports Assessment self-administered Achilles questionnaire; VAS = visual analogue scale; HSR = heavy slow resistance training; ROM = range of motion; SAIS = Subacromial impingement syndrome; SIS = Shoulder impingement syndrome; RCT=randomised controlled trial; RSP = Round shoulder posture; DASH = Disabilities of the Arm, Shoulder and Hand; SF-36 = The 36-Item Short Form Survey; RC = rotator cuff; TrP-DN = trigger point dry needling; CMS = Constant-Murley score; HILT = high-intensity laser therapy; MT = manual therapy; NSAID = a nonsteroidal anti-inflammatory drug; IFC = interferential current; SAPS = Subacromial pain syndrome; LET=lateral epicondylitis tendinopathy; LLLT = low-level laser therapy; PHLE = Progressive high-load exercise; LLE = low-load exercise; AROM = active range of motion; PRE = progressive resistance exercise

Supplementary 7C – Included Studies References (n=110)

1. Agergaard AS, Svensson RB, Hoeffner R, et al. Mechanical properties and UTE-T2* in Patellar tendinopathy: The effect of load magnitude in exercise-based treatment. *Scand J Med Sci Sports*. 2021;31(10):1981-1990. doi:10.1111/sms.14013
2. Agergaard AS, Svensson RB, Malmgaard-Clausen NM, et al. Clinical Outcomes, Structure, and Function Improve With Both Heavy and Moderate Loads in the Treatment of Patellar Tendinopathy: A Randomized Clinical Trial. *Am J Sports Med*. 2021;49(4):982-993. doi:10.1177/0363546520988741
3. Alfredson H, Pietilä T, Jonsson P, et al. Heavy-load eccentric calf muscle training for the treatment of chronic Achilles tendinosis. *Am J Sports Med* 1998;26:360-366.
4. Alfredson H, Nordström P, Pietilä T, et al. Bone mass in the calcaneus after heavy loaded eccentric calf-muscle training in recreational athletes with chronic achilles tendinosis. *Calcif Tissue Int* 1999;64:450-455.
5. Arias-Buría JL, Fernández-de-Las-Peñas C, Palacios-Ceña M, et al. Exercises and dry needling for subacromial pain syndrome: A randomized parallel-group trial. *J Pain* 2017;18:11-18.
6. Arias-Buría JL, Truyols-Domínguez S, Valero-Alcaide R, et al. Ultrasound-guided percutaneous electrolysis and eccentric exercises for subacromial pain syndrome: a randomized clinical trial. *Evidence-Based Complementary and Alternative Medicine* 2015;315219-9.
7. Bağcıer F, Külcü DG, Mesci N, Temel MH. The effect of kinesiotaping on pain, functionality and ultrasound parameters in patients with shoulder impingement syndrome: A randomised sham-controlled study. *Turk Osteoporoz Dergisi* 2021;26(3):151-159.5
8. Bahr R, Fossan B, Løken S, et al. Surgical treatment compared with eccentric training for patellar tendinopathy (jumper's knee): a randomized, controlled trial. *JBJS* 2006;88:1689-1698.
9. Balius R, Álvarez G, Baró F, et al. A 3-arm randomized trial for Achilles tendinopathy: eccentric training, eccentric training plus a dietary supplement containing mucopolysaccharides, or passive stretching plus a dietary supplement containing mucopolysaccharides. *Current Therapeutic Research* 2016;78:1-7.
10. Berg OK, Paulsberg F, Brabant C, et al. High-Intensity Shoulder Abduction Exercise in Subacromial Pain Syndrome. *Med Sci Sports Exerc.* 2021;53(1):1-9
11. Beyer R, Kongsgaard M, Hougs Kjær B, et al. Heavy slow resistance versus eccentric training as treatment for Achilles tendinopathy: a randomized controlled trial. *Am J Sports Med* 2015;43:1704-1711.
12. Blume C, Wang-Price S, Trudelle-Jackson E, et al. Comparison of eccentric and concentric exercise interventions in adults with subacromial impingement syndrome. *International journal of sports physical therapy* 2015;10:441.
13. Boudreau N, Gaudreault N, Roy J, et al. The Addition of Glenohumeral Adductor Coactivation to a Rotator Cuff Exercise Program for Rotator Cuff Tendinopathy: A Single-Blind Randomized Controlled Trial. *J Orthop Sports Phys Ther* 2019;49:126-135.
14. Breda SJ, de Vos RJ, Krestin GP, Oei EHG. Decreasing patellar tendon stiffness during exercise therapy for patellar tendinopathy is associated with better outcome. *J Sci Med Sport*. 2022;25(5):372-378
15. Breda SJ, Oei EHG, Zwerver J, et al. Effectiveness of progressive tendon-loading exercise therapy in patients with patellar tendinopathy: a randomised clinical trial. *Br J Sports Med* 2020;55:501-9.

16. Chaconas EJ, Kolber MJ, Hanney WJ, et al. Shoulder external rotator eccentric training versus general shoulder exercise for subacromial pain syndrome: a randomized controlled trial. *International journal of sports physical therapy* 2017;12:1121-1133.
17. Cheng AS, Hung L. Randomized controlled trial of workplace-based rehabilitation for work-related rotator cuff disorder. *J Occup Rehabil* 2007;17:487-503.
18. Cho S, Shin Y. Effect of rehabilitation and prolotherapy on pain and functional performance in patients with chronic patellar tendinopathy. *Gazzetta Medica Italiana Archivio per le Scienze Mediche* 2017;176:330-337.
19. Christiansen DH, Hjort J. Group-based exercise, individually supervised exercise and home-based exercise have similar clinical effects and cost-effectiveness in people with subacromial pain: a randomised trial. *J Physiother*. 2021;67(2):124-131.
20. Çorum M, Başoğlu C, Yavuz H, Aksoy C. Comparison of the effectiveness of radial extracorporeal shock wave therapy and supervised exercises with neuromuscular inhibition technique in lateral epicondylitis: A randomized-controlled trial. *Turk J Phys Med Rehabil*. 2021;67(4):439-448.
21. de Jonge, S. de Vos R, van Schie H,T.M. Verhaar, J, et al. One-year follow-up of a randomised controlled trial on added splinting to eccentric exercises in chronic midportion Achilles tendinopathy. *Br J Sports Med* 2008;44:673-677.
22. De Vos RJ, Weir A, Visser R, et al. The additional value of a night splint to eccentric exercises in chronic midportion Achilles tendinopathy: a randomised controlled trial. *Br J Sports Med* 2007;41:e5
23. Dejaco B, Habets B, van Loon C, et al. Eccentric versus conventional exercise therapy in patients with rotator cuff tendinopathy: a randomized, single blinded, clinical trial. *Knee Surg Sports Traumatol Arthrosc* 2017;25:2051-2059.
24. Dimitrios S, Pantelis M, Kalliopi S. Comparing the effects of eccentric training with eccentric training and static stretching exercises in the treatment of patellar tendinopathy. A controlled clinical trial. *Clin Rehabil* 2012;26:423-430.
25. Dimitrios S, Pantelis M. Comparing Two Exercise Programmes for the Management of Lateral Elbow Tendinopathy (Tennis Elbow/Lateral Epicondylitis) - A Controlled Clinical Trial. The Open Access Journal of Science and Technology. 2013;1(4):1-8.
26. Dogan C, Ketenci S, Uzuner B, Şen HE, Bilgici A, Alayli G, Ömer KU. Comparison of subacromial corticosteroid injection and physical therapy in patients with subacromial impingement syndrome: A prospective, randomized trial. Journal of Experimental and Clinical Medicine. 2021 Oct 1;38(4):511-5.
27. Dupuis F, Barrett E, Dubé M, et al. Cryotherapy or gradual reloading exercises in acute presentations of rotator cuff tendinopathy: a randomised controlled trial. *BMJ open sport & exercise medicine* 2018;4: e000477.
28. Eliason A, Harringe M, Engström B, Werner S. Guided exercises with or without joint mobilization or no treatment in patients with subacromial pain syndrome: A clinical trial. *J Rehabil Med*. 2021;53(5):jrm00190.
29. Ganderton C, Semciw A, Cook J, Moreira E, Pizzari T. Gluteal loading versus sham exercises to improve pain and dysfunction in postmenopausal women with greater trochanteric pain syndrome: a randomized controlled trial. *J Women's Health* 2018;27(6):815-829.
30. Gatz M, Betsch M, Dirrichs T, et al. Eccentric and Isometric Exercises in Achilles Tendinopathy Evaluated by the VISA-A Score and Shear Wave Elastography. *Sports health* 2020;12:373-81.
31. Giray E, Karali-Bingul D, Akyuz G. The Effectiveness of Kinesiotaping, Sham Taping or Exercises Only in Lateral Epicondylitis Treatment: A Randomized Controlled Study. *PM R* 2019;11:681-693.

32. Habets B, van Cingel REH, Backx FJG, van Elten HJ, Zuithoff P, Huisstede BMA. No Difference in Clinical Effects When Comparing Alfredson Eccentric and Silbernagel Combined Concentric-Eccentric Loading in Achilles Tendinopathy: A Randomized Controlled Trial. *Orthop J Sports Med*. 2021;9(10):23259671211031254.
33. Hallgren HC, Holmgren T, Oberg B, et al. A specific exercise strategy reduced the need for surgery in subacromial pain patients. *Br J Sports Med* 2014;48:1431-1436.
34. Hallgren HC, Adolfsson LE, Johansson K, et al. Specific exercises for subacromial pain: Good results maintained for 5 years. *Acta Orthopaedica* 2017;88:600-605.
35. Heron SR, Woby SR, Thompson DP. Comparison of three types of exercise in the treatment of rotator cuff tendinopathy/shoulder impingement syndrome: a randomised control trial assessing. *Physiotherapy* 2017;103:167-173.
36. Hopewell S, Keene DJ, Heine P, et al. Progressive exercise compared with best-practice advice, with or without corticosteroid injection, for rotator cuff disorders: the GRASP factorial RCT [published correction appears in Health Technol Assess. 2022 Aug;25(48):159-160]. *Health Technol Assess*. 2021;25(48):1-158.
37. Hotta GH, Gomes de Assis Couto A, Cools AM, et al. Effects of adding scapular stabilization exercises to a periscapular strengthening exercise program in patients with subacromial pain syndrome: A randomized controlled trial. *Musculoskelet Sci Pract* 2020;49:102171.
38. Johansson KM, Adolfsson LE, Foldevi MOM. Effects of acupuncture versus ultrasound in patients with impingement syndrome: randomized clinical trial. *Phys Ther* 2005;85:490-501.
39. Jonsson P, Alfredson H. Superior results with eccentric compared to concentric quadriceps training in patients with jumper's knee: a prospective randomised study. *Br J Sports Med* 2005;39:847-50
40. Ketola S, Lehtinen J, Arnala I, et al. Does arthroscopic acromioplasty provide any additional value in the treatment of shoulder impingement syndrome?: a two-year randomised controlled trial. *TheJ Bone Joint Surg. British volume* 2009;91:1326-1334.
41. Knobloch K, Schreibmueller L, Longo UG, et al. Eccentric exercises for the management of tendinopathy of the main body of the Achilles tendon with or without an AirHeel Brace. A randomized controlled trial. B: effects of compliance. *Disabil Rehabil* 2008;30.
42. Knobloch K, Schreibmueller L, Kraemer R, et al. Eccentric training and an Achilles wrap reduce Achilles tendon capillary blood flow and capillary venous filling pressures and increase tendon oxygen saturation in insertional and midportion tendinopathy. *Am J Sports Med* 2007;35:673.
43. Knobloch K, Kraemer R, Jagodzinski M, et al. Eccentric training decreases paratendon capillary blood flow and preserves paratendon oxygen saturation in chronic achilles tendinopathy. J Orthop Sports *Phys Ther* 2007;37:269-276.
44. Kongsgaard M, Kovanen V, Aagaard P, et al. Corticosteroid injections, eccentric decline squat training and heavy slow resistance training in patellar tendinopathy. *Scand J Med Sci Sports* 2009;19:790-802.
45. Kromer TO, de Bie RA, Bastiaenen CHG. Effectiveness of physiotherapy and costs in patients with clinical signs of shoulder impingement syndrome: One-year follow-up of a randomized controlled trial. *J Rehabil Med* 2014;46:1029-1036.
46. Kromer TO, de Bie R,A., Bastiaenen CHG. Physiotherapy in patients with clinical signs of shoulder impingement syndrome: a randomized controlled trial. *J Rehabil Med* 2013;45:488-497.
47. Littlewood C, Bateman M, Brown K, et al. A self-managed single exercise programme versus usual physiotherapy treatment for rotator cuff tendinopathy: A randomised controlled trial (the SELF study). *Clin Rehabil* 2016;30:686-696.

48. Luginbuhl R, Brunner F, Schneeberger AG. No effect of forearm band and extensor strengthening exercises for the treatment of tennis elbow: a prospective randomised study. *Chir Organi Mov* 2008;91:35-40.
49. Maenhout AG, Mahieu NN, De Muynck M, et al. Does adding heavy load eccentric training to rehabilitation of patients with unilateral subacromial impingement result in better outcome? A randomized, clinical trial. *Knee Surg Sports Traumatol Arthrosc* 2013;21:1158-1167.
50. Mafi N, Lorentzon R, Alfredson H. Superior short-term results with eccentric calf muscle training compared to concentric training in a randomized prospective multicenter study on patients with chronic Achilles tendinosis. *Knee Surg Sports Traumatol Arthrosc* 2001;9:42-47.
51. Manias P, Stasinopoulos D. A controlled clinical pilot trial to study the effectiveness of ice as a supplement to the exercise programme for the management of lateral elbow tendinopathy [with consumer summary]. *Br J Sports Med* 2006;40:81-85.
52. Martinez-Silvestrini J, Newcomer KL, Gay RE, et al. Chronic lateral epicondylitis: comparative effectiveness of a home exercise program including stretching alone versus stretching supplemented with eccentric or concentric strengthening. *J Hand Ther* 2005;18:411-420.
53. Marzetti E, Rabini A, Piccinini G, et al. Neurocognitive therapeutic exercise improves pain and function in patients with shoulder impingement syndrome: a single-blind randomized controlled clinical trial. *Eur J Phys Rehabil Med* 2014;50:255-264.
54. McCormack JR, Underwood FB, Slaven EJ, et al. Eccentric Exercise Versus Eccentric Exercise and Soft Tissue Treatment (Astym) in the Management of Insertional Achilles Tendinopathy. *Sports health* 2016;8:230-7.
55. Mulligan EP, Huang M, Dickson T, et al. The Effect of Axioscapular and Rotator Cuff Exercise Training Sequence in Patients with Subacromial Impingement Syndrome: a Randomized Crossover Trial. *International journal of sports physical therapy* 2016;11:94-107.
56. Nørregaard J, Larsen CC, Bieler T, et al. Eccentric exercise in treatment of Achilles tendinopathy. *Scand J Med Sci Sports* 2007;17:133-138.
57. Nowotny J, El-Zayat B, Goronzy J, et al. Prospective randomized controlled trial in the treatment of lateral epicondylitis with a new dynamic wrist orthosis. *Eur J Med Res* 2018;23:1-7.
58. Østerås H, Torstensen TA, Østerås B. High-dosage medical exercise therapy in patients with long-term subacromial shoulder pain: a randomized controlled trial. *Physiother Res Int* 2010;15:232-242.
59. Park JY, Park HK, Choi JH, et al. Prospective evaluation of the effectiveness of a home-based program of isometric strengthening exercises: 12-month follow-up. *Clin Orthop Surg* 2010;2:173-178.
60. Pearson J, Rowlands D, Highet R. Autologous blood injection to treat achilles tendinopathy? A randomized controlled trial. *J Sport Rehab* 2012;21:218-24.
61. Pearson SJ, Stadler S, Menz H, et al. Immediate and Short-Term Effects of Short-and Long-Duration Isometric Contractions in Patellar Tendinopathy. *Clin J Sport Med* 2018.
62. Pekyavas NO, Baltaci G. Short-term effects of high-intensity laser therapy, manual therapy, and Kinesio taping in patients with subacromial impingement syndrome. *Lasers in medical science* 2016;31:1133-1141.
63. Petersen W, Welp R, Rosenbaum D. Chronic Achilles tendinopathy: a prospective randomized study comparing the therapeutic effect of eccentric training, the AirHeel brace, and a combination of both. *Am J Sports Med* 2007;35:1659-1667.
64. Peterson M, Butler S, Eriksson M, et al. A randomized controlled trial of exercise versus wait-list in chronic tennis elbow (lateral epicondylosis). *Ups J Med Sci* 2011;116:269-279.

65. Peterson M, Butler S, Eriksson M, et al. A randomized controlled trial of eccentric versus concentric graded exercise in chronic tennis elbow (lateral elbow tendinopathy) [with consumer summary]. *Clin Rehabil* 2014;28:862-872 2014.
66. Praet S, Alzyadat T, Purdam C, et al. Oral supplementation of specific collagen peptides accelerates improvement in Achilles tendon pain and function in combination with a tailored exercise program. *J Bodywork Movement Ther* 2018;22:862-3.
67. Rabusin CL, Menz HB, McClelland JA, et al. Efficacy of heel lifts versus calf muscle eccentric exercise for mid-portion Achilles tendinopathy (HEALTHY): a randomised trial. *Br J Sports Med* 2020:55:486-92.
68. Rabusin CL, Menz HB, McClelland JA*, et al.* Efficacy of heel lifts versus calf muscle eccentric exercise for mid-portion Achilles tendinopathy (HEALTHY): a randomised trial *British Journal of Sports Medicine* 2021;**55:**486-492.
69. Rio E, Purdam C, Girdwood M, et al. Isometric Exercise to Reduce Pain in Patellar Tendinopathy In-Season; Is It Effective "on the Road?". *Clin J Sport Med* 2017;29:188-92
70. Romero-Morales C, Martin-Llantino P, Calvo-Lobo C, et al. Vibration increases multifidus cross-sectional area versus cryotherapy added to chronic non-insertional Achilles tendinopathy eccentric exercise [with consumer summary]. *Phys Ther Sport* 2020;42:61-67.
71. Rompe JD, Nafe B, Furia JP, Maffulli N. Eccentric loading, shock-wave treatment, or a wait-and-see policy for tendinopathy of the main body of tendo Achillis: a randomized controlled trial. *Am J Sports Med* 2007;35(3):374-383.
72. Rompe JD, Furia J, Maffulli N. Eccentric loading versus eccentric loading plus shock-wave treatment for midportion achilles tendinopathy: a randomized controlled trial. *Am J Sports Med* 2009;37:463-470.
73. Rompe JD, Segal NA, Cacchio A, et al. Home training, local corticosteroid injection, or radial shock wave therapy for greater trochanter pain syndrome. *Am J Sports Med* 2009;37:1981-1990
74. Rompe JD, Furia J, Maffulli N. Eccentric loading compared with shock wave treatment for chronic insertional achilles tendinopathy: A randomized, controlled trial.*J Bone Joint Surg* 2008;90:52-61.
75. Roos EM, Engstrom M, Lagerquist A, et al. Clinical improvement after 6 weeks of eccentric exercise in patients with mid-portion Achilles tendinopathy -- a randomized trial with 1-year follow-up [with consumer summary]. *Scand J Med Sci Sports* 2004;14:286-295.
76. Ruffino D, Malliaras P, Marchegiani S, Campana V. Inertial flywheel vs heavy slow resistance training among athletes with patellar tendinopathy: A randomised trial. *Physical Therapy in Sport.* 2021 Nov 1;52:30-7.
77. Şahbaz T, Medin Ceylan C, Karacay BÇ, Korkmaz MD, Dıracoğlu D. Comparison of platelet-rich plasma and extracorporeal shock wave therapy in patients with chronic lateral epicondylitis: A prospective, randomized-controlled study. *Turk J Phys Med Rehabil.* 2021;67(4):490-501.
78. Schydlowsky, P., Szkudlarek, M. & Madsen, O.R. Comprehensive supervised heavy training program versus home training regimen in patients with subacromial impingement syndrome: a randomized trial. *BMC Musculoskelet Disord.* 2022; 23 (52).
79. Şenbursa G, Baltaci G, Atay ÖA. The effectiveness of manual therapy in supraspinatus tendinopathy. Acta *Orthop Traumatol Turc* 2011;45:162-167.
80. Sevier TL, Stegink-Jansen C. Astymtreatment vs. eccentric exercise for lateral elbow tendinopathy: a randomized controlled clinical trial. Peer J 2015;3:e967.
81. Shim BJ, Seo EM, Hwang JT, et al. Comparison of the effectiveness of extensor muscle strengthening exercise by itself, exercise with polydeoxyribonucleotide injection, and

exercise with extracorporeal shockwave therapy in lateral epicondylitis: a randomized controlled trial. *Clin Shoulder Elb*. 2021;24(4):231-238

82. Silbernagel KG, Thomeé R, Eriksson BI, et al. Continued sports activity, using a pain-monitoring model, during rehabilitation in patients with Achilles tendinopathy: a randomized controlled study. *Am J Sports Med* 2007;35:897-906.
83. Silbernagel KG, Thomeé R, Thomeé P, et al. Eccentric overload training for patients with chronic Achilles tendon pain--a randomised controlled study with reliability testing of the evaluation methods. *Scand J Med Sci Sports* 2001;11:197-206.
84. Şimşek HH, Balki S, Keklik SS, et al. Does Kinesio taping in addition to exercise therapy improve the outcomes in subacromial impingement syndrome? A randomized, double-blind, controlled clinical trial. Acta *Orthop Traumatol Turc* 2013;47:104-110.
85. Silder AM, Sherry MA, Sanfilippo J, et al. Clinical and morphological changes following 2 rehabilitation programs for acute hamstring strain injuries: a randomized clinical trial. *J Orthop Sports Phys Ther* 2013;43:284-99.
86. Solomons L, Lee JJY, Bruce M, White LD, Scott A. Intramuscular stimulation vs sham needling for the treatment of chronic midportion Achilles tendinopathy: A randomized controlled clinical trial. *PLoS One*. 2020;15(9):e0238579.
87. Stasinopoulos D, Stasinopoulos I. Comparison of effects of eccentric training, eccentric-concentric training, and eccentric-concentric training combined with isometric contraction in the treatment of lateral elbow tendinopathy. *J Hand Ther* 2017;30:13-19.
88. Stasinopoulos D, Stasinopoulos I. Comparison of effects of Cyriax physiotherapy, a supervised exercise programme and polarized polychromatic non-coherent light (Bioptron light) for the treatment of lateral epicondylitis. *Clin Rehabil* 2006;20:12-23.
89. Stasinopoulos D, Stasinopoulos I, Pantelis M, et al. Comparison of effects of a home exercise programme and a supervised exercise programme for the management of lateral elbow tendinopathy. *Br J Sports Med* 2010;44:579-583.
90. Stasinopoulos D, Manias P. Comparing two eccentric exercise programmes for the management of Achilles tendinopathy. A pilot trial. *J Bodywork Movement Ther* 2013;17(3):309-315.
91. Stefansson SH, Brandsson S, Langberg H, et al. Using Pressure Massage for Achilles Tendinopathy: A Single-Blind, Randomized Controlled Trial Comparing a Novel Treatment Versus an Eccentric Exercise Protocol. *Orthopaedic journal of sports medicine* 2019;7:2325967119834284.
92. Steunebrink M, Zwerver J, Brandsema R, et al. Topical glyceryl trinitrate treatment of chronic patellar tendinopathy: a randomised, double-blind, placebo-controlled clinical trial. *Br J Sports Med* 2013;47:34-39.
93. Stevens M, Tan C. Effectiveness of the Alfredson protocol compared with a lower repetition-volume protocol for midportion Achilles tendinopathy: a randomized controlled trial. *J Orthop Sports Phys Ther* 2014;44:59-67.
94. Svernlov B, Adolfsson L. Non-operative treatment regime including eccentric training for lateral humeral epicondylalgia. *Scand J Med Sci Sports* 2001;11:328-334.
95. Tonks JH, Pai SK, Murali SR. Steroid injection therapy is the best conservative treatment for lateral epicondylitis: A prospective randomised controlled trial. *Int J Clin Pract* 2007;61:240-246.
96. Turgut E, Duzgun I, Baltaci G. Effects of Scapular Stabilization Exercise Training on Scapular Kinematics, Disability, and Pain in Subacromial Impingement: A Randomized Controlled Trial. *Arch Phys Med Rehabil* 2017;98:1915.
97. Vallés-Carrascosa E, Gallego-Izquierdo T, Jiménez-Rejano JJ, et al. Pain, motion and function comparison of two exercise protocols for the rotator cuff and scapular stabilizers in patients with subacromial syndrome. *J Hand Ther* 2018;31:227-37.

98. Van Ark M, Cook JL, Docking SI, et al. Do isometric and isotonic exercise programs reduce pain in athletes with patellar tendinopathy in-season? A randomised clinical trial. J Sci Med Sport 2016;19:702-706.
99. Vinuesa-Montoya S, Aguilar-Ferrándiz ME, Matarán-Peñarrocha GA, et al. A Preliminary Randomized Clinical Trial on the Effect of Cervicothoracic Manipulation Plus Supervised Exercises Vs a Home Exercise Program for the Treatment of Shoulder Impingement. *Journal of chiropractic medicine.* 2017;16:85-93.
100. Visnes H, Hoksrud A, Cook J, et al. No effect of eccentric training on jumper's knee in volleyball players during the competitive season: a randomized clinical trial. *Scand J Med Sci Sports* 2005;15:215.
101. Vuvan, V, Vicenzino B, Mellor R, et al. Unsupervised Isometric Exercise versus Wait-and-See for Lateral Elbow Tendinopathy. *Med Sci Sports Exerc* 2020;52:287-295.
102. Walther M, Werner A, Stahlschmidt T, et al. The subacromial impingement syndrome of the shoulder treated by conventional Physiotherapy, self-training, and a shoulder brace: Results of a prospective, randomized study. *J Shoulder Elbow Surg* 2004;13:417-423.
103. Wegener RL, Brown T, O'Brien L. A randomized controlled trial of comparative effectiveness of elastic therapeutic tape, sham tape or eccentric exercises alone for lateral elbow tendinosis. *Hand Therapy* 2016;21:131-139.
104. Wen DY, Schultz BJ, Schaal B, et al. Eccentric strengthening for chronic lateral epicondylosis: a prospective randomized study. *Sports health* 2011;3:500-503.
105. Werner A, Walther M, Ilg A, et al. Self-training versus conventional Physiotherapy in subacromial impingement syndrome. *Z Orthop Ihre* 2002;140:375-380.
106. Wiedmann M, Mauch F, Huth J, et al. Treatment of mid-portion Achilles tendinopathy with eccentric training and its effect on neovascularization. *Sports Orthopaedics and Traumatology* 2017;33:278-285.
107. Yelland MJ, Sweeting KR, Lyftogt JA, et al. Prolotherapy injections and eccentric loading exercises for painful Achilles tendinosis: a randomised trial. *Br J Sports Med* 2011;45: 421-8.
108. Yilmaz K, Yigiter Bayramlar K, Ayhan C, Tufekci O. Investigating the effects of neuromobilization in lateral epicondylitis. *J Hand Ther.* 2022;35(1):97-106.
109. Young MA, Cook JL, Purdam CR, et al. Eccentric decline squat protocol offers superior results at 12 months compared with traditional eccentric protocol for patellar tendinopathy in volleyball players [with consumer summary]. *Br J Sports Med* 2005;39:102-105.
110. Yu J, Park D, Lee G. Effect of eccentric strengthening on pain, muscle strength, endurance, and functional fitness factors in male patients with achilles tendinopathy. *Am J Phys Med Rehabil* 2013;92:68-76.
