# Supplementary material for: Effect of resistance exercise dose components for tendinopathy management: a systematic review with meta-analysis
Source: Br J Sports Med. 2023 May 11;57(20):1327–34. doi: 10.1136/bjsports-2022-105754 (PMC10579176; doi:10.1136/bjsports-2022-105754)
Supplement: Supplementary data [file bjsports-2022-105754supp008.pdf]

SF8: Risk of Bias and confidence in cumulative evidence assessments

Supplementary table 8A. Risk of Bias assessments per study. Assessments were made for each outcome in a study with the mode value selected and presented here. In the case of non-randomised studies, the first two domains (randomisation, allocation concealment) were judged as high risk as there was no control over allocation; the remaining domains were judged as for randomised studies.

**Author, Year** (reference)*	**Random sequence generation**	**Allocation concealment**	**Blinding of participants/personnel**	**Blinding of outcome assessment**	**Incomplete outcome bias**	**Selective reporting**	**Other bias****
Agregaard et al 2021[1]	Low risk	Low risk	High risk	Low risk	Low risk	Low risk	High risk
Agregaard et al 2021[2]	Low risk	Low risk	High risk	Low risk	Low risk	Low risk	Unclear
Alfredson et al 1998[3]	High risk	Unclear	High risk	Unclear	Low risk	Unclear	High risk
Alfredson et al 1999[4]	High risk	High risk	Unclear	Unclear	Low risk	Unclear	High risk
Arias-Buría et al 2015[5]	Low risk	Low risk	High risk	Low risk	Low risk	Unclear	Low risk
Arias-Buría et al 2017[6]	Low risk	Low risk	Unclear	Low risk	Unclear	Low risk	High risk
Bagcier et al 2021[7]	Low risk	Unclear	Low risk	Low risk	Low risk	Low risk	Low risk
Bahr et al 2006[8]	Low risk	Low risk	High risk	Low risk	Low risk	Unclear	Low risk
Balius et al 2016[9]	Low risk	Low risk	Unclear	Low risk	Low risk	Unclear	Low risk
Berg et al 2021[10]	Low risk	Unclear	Unclear	Unclear	Low risk	Low risk	High risk
Beyer et al 2015[11]	Low risk	Low risk	Low risk	Low risk	Low risk	Low risk	High risk
Blume et al 2015[12]	Unclear	Low risk	Low risk	Low risk	Low risk	Unclear	Low risk
Boudreau et al 2019[13]	Low risk	Low risk	Low risk	Low risk	Low risk	Low risk	Low risk
Breda et al 2020[14]	Low risk	Low risk	High risk	Low risk	Low risk	High risk	High risk
Breda et al 2022[15]	Low risk	Low risk	High risk	Low risk	Low risk	Low risk	High risk
Chaconas et al 2017[16]	Low risk	Unclear	Unclear	Low risk	High risk	Unclear	High risk
Cheng et al 2007[17]	High risk	High risk	Unclear	Unclear	Unclear	Unclear	High risk
Cho et al 2017[18]	High risk	High risk	Unclear	Unclear	Low risk	Low risk	Unclear

**Author, Year** (reference) *	**Random sequence generation**	**Allocation concealment**	**Blinding of participants/personnel**	**Blinding of outcome assessment**	**Incomplete outcome bias**	**Selective reporting**	**Other bias****
Christiansen et al 2021[19]	Low risk	Low risk	Unclear	Unclear	Low risk	Low risk	High risk
Corum et al 2021[20]	Low risk	Low risk	High risk	Low risk	High risk	Low risk	High risk
de Jonge et al 2008[21]	Unclear	Low risk	Low risk	Low risk	Low risk	Low risk	Low risk
de Vos et al 2007[22]	Low risk	Unclear	Low risk	Low risk	Unclear	Low risk	High risk
Dejaco et al 2017[23]	Low risk	Low risk	Low risk	Low risk	Low risk	High risk	Low risk
Dimitrios et al 2012[24]	High risk	High risk	Low risk	Low risk	Low risk	Unclear	High risk
Dimitrios et al 2012[25]	High risk	High risk	Low risk	Low risk	Low risk	Unclear	High risk
Dogan et al 2021[26]	Unclear	Unclear	Unclear	Unclear	Unclear	Unclear	High risk
Dupuis et al 2018[27]	Low risk	Low risk	High risk	Low risk	Low risk	Low risk	High risk
Eliason et al 2021[28]	High risk	Low risk	Low risk	Low risk	Low risk	Low risk	High risk
Ganderton et al 2018[29]	Low risk	Low risk	Low risk	Low risk	Low risk	Low risk	High risk
Gatz et al 2020[30]	Low risk	Low risk	Low risk	Low risk	Unclear	Unclear	High risk
Giray et al 2019[31]	Low risk	Low risk	High risk	Low risk	Low risk	Low risk	High risk
Habets et al 2021[32]	Low risk	Low risk	Unclear	Low risk	Low risk	Low risk	Low risk
Hallgren et al 2014[33]	High risk	Low risk	High risk	Low risk	Unclear	Low risk	Low risk
Hallgren et al 2017[34]	Unclear	Unclear	Low risk	Low risk	Low risk	Low risk	Low risk
Heron et al 2017[35]	Low risk	Low risk	Low risk	Low risk	High risk	High risk	Low risk
Hopewell et al 2021[36]	Low risk	Low risk	High risk	Low risk	Low risk	Low risk	Low risk
Hotta et al 2020[37]	Low risk	Low risk	High risk	Low risk	Low risk	Low risk	High risk
Johansson et al 2005[38]	Low risk	Unclear	Unclear	Low risk	Low risk	Unclear	High risk

**Author, Year** (reference)*	**Random sequence generation**	**Allocation concealment**	**Blinding of participants/personnel**	**Blinding of outcome assessment**	**Incomplete outcome bias**	**Selective reporting**	**Other bias****
Jonsson 2009[39]	Unclear	Unclear	Unclear	Unclear	Unclear	Unclear	Unclear
Ketola et al 2009[40]	Low risk	Low risk	Unclear	Low risk	Low risk	Unclear	High risk
Knobloch et al 2007[41]	Low risk	Low risk	Unclear	Unclear	High risk	Unclear	High risk
Knobloch et al 2007[42]	Unclear	Unclear	High risk	Low risk	Unclear	Unclear	High risk
Knobloch et al 2008[43]	Unclear	Low risk	Unclear	Unclear	Unclear	Unclear	High risk
Kongsgaard et al[44]	Low risk	Low risk	High risk	Low risk	Low risk	Unclear	Low risk
Kromer et al 2013[45]	Low risk	Low risk	Low risk	Low risk	Low risk	Low risk	Low risk
Kromer et al 2014[46]	Low risk	Low risk	High risk	High risk	Low risk	Low risk	Low risk
Littlewood et al 2016[47]	Low risk	Low risk	High risk	High risk	Unclear	Unclear	High risk
Luginbuhl et al 2008[48]	Unclear	Unclear	Unclear	Unclear	Unclear	Unclear	High risk
Maenhout et al 2013[49]	Unclear	High risk	High risk	High risk	Low risk	Unclear	Low risk
Mafi et al 2001[50]	Low risk	Unclear	Unclear	Unclear	Unclear	Unclear	High risk
Manias et al 2006[51]	High risk	High risk	High risk	High risk	Low risk	Unclear	Unclear
Martinez-Silvestrini et al 2005[52]	Unclear	Unclear	Unclear	Unclear	Low risk	Unclear	High risk
Marzetti et al 2014[53]	Low risk	Low risk	Low risk	Low risk	Low risk	Low risk	Low risk
McCormack et al 2016[54]	Low risk	Low risk	Unclear	Unclear	Low risk	Low risk	Low risk
Mulligan et al 2016[55]	Low risk	Low risk	Low risk	Low risk	Low risk	Unclear	High risk
Nørregaard et al 2007[56]	Low risk	Low risk	Unclear	Unclear	Unclear	Unclear	High risk

**Author, Year** (reference)*	**Random sequence generation**	**Allocation concealment**	**Blinding of participants/personnel**	**Blinding of outcome assessment**	**Incomplete outcome bias**	**Selective reporting**	**Other bias****
Nowotny et al 2018[57]	Low risk	Unclear	Low risk	Low risk	High risk	Unclear	High risk
Østerås et al 2010[58]	Low risk	Low risk	High risk	High risk	Low risk	Unclear	High risk
Park et al 2010[59]	Low risk	Unclear	Unclear	Unclear	Unclear	Unclear	High risk
Pearson et al 2012[60]	Unclear	Unclear	High risk	Unclear	Low risk	Unclear	High risk
Pearson et al 2018[61]	Low risk	Low risk	High risk	Low risk	Low risk	Low risk	High risk
Pekyavas et al 2016[62]	Low risk	Low risk	High risk	Low risk	Unclear	Unclear	Low risk
Petersen et al 2007[63]	Low risk	Unclear	Unclear	Unclear	Unclear	Unclear	High risk
Peterson et al 2011[64]	Low risk	Low risk	Unclear	High risk	Low risk	Low risk	Low risk
Peterson et al 2014[65]	Low risk	Unclear	Low risk	High risk	Low risk	Low risk	Low risk
Praet et al 2019[66]	Low risk	Low risk	Low risk	Low risk	Low risk	Unclear	Low risk
Rabusin et al 2020[67]	Low risk	Low risk	High risk	High risk	Low risk	Low risk	High risk
Rabusin et al 2021[68]	Low risk	Low risk	High risk	High risk	Low risk	Low risk	High risk
Rio et al 2017[69]	Low risk	Low risk	Low risk	Low risk	Low risk	Low risk	High risk
Romero-Morales et al 2020[70]	Unclear	Unclear	Unclear	Unclear	Low risk	Low risk	High risk
Rompe et al 2007[71]	Low risk	Low risk	Unclear	Low risk	Low risk	Unclear	Low risk
Rompe et al 2008[72]	Low risk	Low risk	Unclear	Low risk	Low risk	Unclear	Unclear
Rompe et al 2009[73]	Unclear	Low risk	High risk	High risk	Low risk	Unclear	Low risk
Rompe et al 2009[74]	Low risk	Low risk	High risk	Low risk	Low risk	Unclear	Low risk
Roos et al 2004[75]	Low risk	Unclear	Unclear	Low risk	Low risk	Unclear	Low risk
Ruffino et al 2021[76]	Low risk	Low risk	High risk	Low risk	Low risk	Unclear	High risk
Sahbaz et al 2021[77]	Low risk	Unclear	Unclear	Unclear	Low risk	Low risk	Unclear
Schydlowsky 2022[78]	Unclear	Low risk	High risk	Low risk	Low risk	Low risk	High risk

**Author, Year** (reference) *	**Random sequence generation**	**Allocation concealment**	**Blinding of participants/personnel**	**Blinding of outcome assessment**	**Incomplete outcome bias**	**Selective reporting**	**Other bias****
Şenbursa et al 2011[79]	Low risk	Unclear	Unclear	Unclear	Low risk	Unclear	Low risk
Sevier et al 2015[80]	Low risk	Unclear	High risk	High risk	High risk	Unclear	High risk
Shim et al 2021[81]	Low risk	Low risk	High risk	Low risk	Low risk	Unclear	High risk
Silbernagel et al 2001[82]	Unclear	Unclear	Unclear	Unclear	Unclear	Unclear	High risk
Silbernagel et al 2007[83]	Low risk	Low risk	High risk	High risk	Low risk	Unclear	Low risk
Simşek et al 2013[84]	Unclear	Unclear	Unclear	Low risk	Unclear	Unclear	Unclear
Slider et al 2013[85]	Low risk	Unclear	Low risk	Low risk	Low risk	Unclear	Unclear
Solomons et al 2020[86]	Low risk	Low risk	Low risk	Low risk	Low risk	Low risk	Low risk
Stasinopoulos 2013[87]	High risk	High risk	High risk	Low risk	Low risk	Unclear	High risk
Stasinopoulos et al 2006[88]	High risk	High risk	Unclear	Low risk	Low risk	Unclear	High risk
Stasinopoulos et al 2010[89]	High risk	High risk	Low risk	Low risk	Low risk	Unclear	High risk
Stasinopoulos et al 2017[90]	Low risk	Unclear	Low risk	Low risk	Low risk	Unclear	High risk
Stefansson et al 2019[91]	Low risk	Unclear	High risk	Low risk	High risk	Unclear	Low risk
Steunebrink et al 2013[92]	Low risk	Low risk	Low risk	Low risk	Low risk	Unclear	Low risk
Stevens et al 2014[93]	Unclear	Unclear	High risk	High risk	Unclear	Unclear	High risk
Svernlov et al 2001[94]	High risk	High risk	Unclear	Unclear	Unclear	Unclear	High risk
Tonks et al 2007[95]	Low risk	Low risk	Low risk	High risk	High risk	Low risk	Low risk
Turgut et al 2017[96]	Low risk	Unclear	Unclear	Unclear	High risk	Unclear	Low risk
Vallés-Carrascosa	Low risk	Low risk	Low risk	High risk	Low risk	Low risk	High risk

**Author, Year** (reference)*	**Random sequence generation**	**Allocation concealment**	**Blinding of participants/personnel**	**Blinding of outcome assessment**	**Incomplete outcome bias**	**Selective reporting**	**Other bias****
et al 2018[97]							
vanArk et al 2016[98]	Low risk	Low risk	Low risk	Unclear	Unclear	Low risk	Low risk
Vinuesa-Montoya et al 2017[99]	Low risk	Low risk	High risk	Low risk	Low risk	Low risk	Low risk
Visnes et al 2005[100]	Low risk	Low risk	High risk	Low risk	Unclear	Unclear	Unclear
Vuvan et al 2019[101]	Low risk	Low risk	High risk	High risk	Low risk	Low risk	Low risk
Walther et al 2004[102]	Unclear	Unclear	Unclear	Unclear	Low risk	Unclear	Unclear
Wegener et al 2016[103]	Low risk	Low risk	High risk	Low risk	Low risk	Unclear	High risk
Wen et al 2011[104]	Unclear	Unclear	Low risk	Unclear	High risk	Unclear	High risk
Werner et al 2002[105]	Low risk	Unclear	Unclear	Unclear	Unclear	Unclear	High risk
Wiedmann et al 2017[106]	Low risk	Low risk	Unclear	Unclear	Unclear	Unclear	High risk
Yelland et al 2011[107]	Low risk	Low risk	High risk	Low risk	Low risk	Unclear	Low risk
Yilmaz et al 2022[108]	Unclear	Low risk	Unclear	Low risk	Low risk	Unclear	High risk
Young et al 2005[109]	Unclear	Unclear	High risk	Low risk	High risk	Unclear	High risk
Yu et al 2013[110]	Low risk	Low risk	Low risk	Unclear	Low risk	Unclear	Unclear

*Included studies reference list in supplementary SF7-B.

**Note: The "other bias" category captures any bias not covered in the other domains. Specifically in this review we used the examples below to help with judging "other bias". Criteria for 'High risk' on assessing "other bias" include:

- Measurement of the outcome differed between intervention groups. Outcomes would usually be comparable across intervention groups in studies with pre-specific outcomes. For example: does one treatment group result in more frequent clinic visits (e.g., home exercise program vs. physiotherapy-led treatment)?
- Inappropriate outcome measurement tools used and/or uncertainty in their validity and reliability e.g., self-reported measures may have higher risk of bias than clinically observed outcomes.
- Design-specific bias:
  - Duration of follow-up that is different across comparison groups within a study, this difference could be a source of bias.

  - The issue of study populations that are systematically different between comparison groups within a study (e.g., important baseline imbalances) may be a source of bias; the population selected for the focus of the study (e.g., inclusion and exclusion criteria) would need to be considered.
- Failure of study to maintain fidelity to the intervention protocol resulting in performance bias.
- Conflict of Interest from Sponsor Bias resulting in:
  - The selection of designs and hypotheses; choosing non-inferiority rather than superiority approaches, picking comparison drugs and doses, choosing outcomes, or using composite endpoints (e.g., quality of life) without presenting data on individual endpoints.
  - Selective outcome reporting e.g., “cherry-picking” from multiple endpoints.
  - Biased presentation of results.
  - Publication bias.

Supplementary table 8B. Overall RoB and confidence in cumulative evidence assessment for moderator levels in meta-regression investigating resistance exercise intensity.

**Moderator**	**Model**	**Overall RoB**	**Inconsistency**	**Imprecision**	**Indirectness**	**Small study-effects**	**Confidence in Evidence**
Intensity: Body mass	All tendinopathies/ Large-effects	Low risk	Low risk	High risk	Low risk	High risk	Low
Intensity: Additional	All tendinopathies/ Large-effects	Low risk	Low risk	Low risk	Low risk	High risk	Moderate
Intensity: Body mass	Achilles/ Large-effects	Low risk	Low risk	High risk	Low risk	High risk	Low
Intensity: Additional	Achilles / Large-effects	Low risk	Low risk	High risk	Low risk	High risk	Low
Intensity: Body mass	RCRSP/ Large-effects	Low risk	Low risk	High risk	Low risk	High risk	Low
Intensity: Additional	RCRSP/ Large-effects	Low risk	Low risk	Low risk	Low risk	High risk	Moderate
Intensity: Body mass	Patellar/ Large-effects	Low risk	High risk	High risk	Low risk	High risk	Very low
Intensity: Additional	Patellar/ Large-effects	Low risk	Low risk	High risk	Low risk	High risk	Low
Intensity: Body mass	All tendinopathies/ Small-effects	Low risk	Low risk	High risk	Low risk	High risk	Low
Intensity: Additional	All tendinopathies/ Small -effects	Low risk	Low risk	Low risk	Low risk	High risk	Moderate
Intensity: Body mass	Achilles/ Small-effects	Low risk	High risk	High risk	Low risk	High risk	Very low
Intensity: Additional	Achilles / Small-effects	Low risk	High risk	High risk	Low risk	High risk	Very low
Intensity: Body mass	RCRSP / Small-effects	Low risk	High risk	High risk	Low risk	High risk	Very low
Intensity: Additional	RCRSP / Small-effects	Low risk	Low risk	Low risk	Low risk	High risk	Moderate

Supplementary table 8C. Overall RoB and confidence in cumulative evidence assessment for moderator levels in meta-regression investigating resistance exercise frequency.

**Moderator**	**Model**	**Overall RoB**	**Inconsistency**	**Imprecision**	**Indirectness**	**Small study-effects**	**Confidence in evidence**
Frequency: Less than daily	All tendinopathies/ Large-effects	Low risk	High risk	High risk	Low risk	High risk	Low
Frequency: Daily	All tendinopathies/ Large-effects	Low risk	Low risk	Low risk	Low risk	High risk	Moderate
Frequency: More than once per day	All tendinopathies/ Large-effects	Low risk	Low risk	Low risk	Low risk	High risk	Moderate
Frequency: Less than daily	Achilles/ Large-effects	Low risk	High risk	High risk	Low risk	High risk	Very low
Frequency: Daily	Achilles/ Large-effects	Low risk	Low risk	High risk	Low risk	High risk	Low
Frequency: More than once per day	Achilles/ Large-effects	Low risk	Low risk	Low risk	Low risk	High risk	Moderate
Frequency: Less than daily	RCRSP/ Large-effects	Low risk	Low risk	Low risk	Low risk	High risk	Moderate
Frequency: Daily	RCRSP/ Large-effects	Low risk	Low risk	Low risk	Low risk	High risk	Moderate
Frequency: More than once per day	RCRSP/ Large-effects	Low risk	Low risk	High risk	Low risk	High risk	Low
Frequency: Less than daily	All tendinopathies/ Small-effects	Low risk	High risk	High risk	Low risk	High risk	Very low
Frequency: Daily	All tendinopathies/ Small-effects	Low risk	Low risk	High risk	Low risk	High risk	Low
Frequency: More than once per day	All tendinopathies/ Small-effects	Low risk	Low risk	Low risk	Low risk	High risk	Moderate
Frequency: Less than daily	Achilles/ Small-effects	Low risk	High risk	High risk	Low risk	High risk	Very low
Frequency: Daily	Achilles/ Small-effects	Low risk	Low risk	High risk	Low risk	High risk	Low
Frequency: More than once per day	Achilles/ Small-effects	High risk	High risk	Low risk	Low risk	High risk	Very low
Frequency: Less than daily	RCRSP/ Small-effects	Low risk	Low risk	Low risk	Low risk	High risk	Moderate
Frequency: Daily	RCRSP/ Small-effects	Low risk	High risk	High risk	Low risk	High risk	Very low

Frequency: More than once per day	RCRSP/ Small-effects	Low risk	Low risk	High risk	Low risk	High risk	Low

Supplementary table 8D. Overall RoB and confidence in cumulative evidence assessment for moderator levels in meta-regression investigating resistance exercise volume.

**Moderator**	**Model**	**Overall RoB**	**Inconsistency**	**Imprecision**	**Indirectness**	**Small study-effects**	**Confidence in evidence**
Volume: Low	All tendinopathies/ Large-effects	Low risk	Low risk	Low risk	Low risk	High risk	Moderate
Volume: High	All tendinopathies/ Large-effects	Low risk	Low risk	Low risk	Low risk	High risk	Moderate
Volume: Low	Achilles/ Large-effects	Low risk	High risk	High risk	Low risk	Low risk	Low
Volume: High	Achilles / Large-effects	Low risk	Low risk	Low risk	Low risk	High risk	Moderate
Volume: Low	RCRSP/ Large-effects	Low risk	Low risk	Low risk	Low risk	High risk	Moderate
Volume: High	RCRSP/ Large-effects	Low risk	Low risk	Low risk	Low risk	High risk	Moderate
Volume: Low	Patellar/ Large-effects	Low risk	Low risk	High risk	Low risk	High risk	Low
Volume: High	Patellar/ Large-effects	Low risk	Low risk	High risk	Low risk	High risk	Low
Volume: Low	Elbow/ Large-effects	Low risk	Low risk	High risk	Low risk	High risk	Low
Volume: High	Elbow/ Large-effects	Low risk	Low risk	High risk	Low risk	High risk	Low
Volume: Low	All tendinopathies/ Small-effects	Low risk	Low risk	Low risk	Low risk	High risk	Moderate
Volume: High	All tendinopathies/ Small-effects	Low risk	Low risk	Low risk	Low risk	High risk	Moderate
Volume: Low	RCRSP/ Small-effects	Low risk	Low risk	Low risk	Low risk	High risk	Moderate
Volume: High	RCRSP/ Small-effects	Low risk	Low risk	High risk	Low risk	High risk	Low
Volume: Low	Patellar/ Small-effects	Low risk	Low risk	High risk	Low risk	High risk	Low
Volume: High	Patellar/ Small-effects	Low risk	Low risk	High risk	Low risk	High risk	Low
