# Supplementary material for: Effect of resistance exercise dose components for tendinopathy management: a systematic review with meta-analysis
Source: Br J Sports Med. 2023 May 11;57(20):1327–34. doi: 10.1136/bjsports-2022-105754 (PMC10579176; doi:10.1136/bjsports-2022-105754)
Supplement: Supplementary data [file bjsports-2022-105754supp009.pdf]

SF9: Moderator analyses comparing averaged pooled effect sizes

Supplementary table 9A. Moderator analysis comparing average pooled effect size for body weight interventions versus interventions including additional external load. Results presented across all tendinopathies and individual tendinopathies.

Moderator		**Pooled** SMD_{pre} **estimate [95% CrI]**	**Probability**	**Study VPC [75% CrI]**	**Outcome VPC [75% CrI]**	**Measurement occasion VPC [75% CrI]**	**Confidence in evidence**
**Large Effects (All tendinopathies)**	Body mass 169 outcomes 28 treatment arms)	0.91 [0.58 to 1.2]	*p*(Body weight < Additional) = 0.998	0.78 [0.74 to 0.84]	0.18 [0.14 to 0.23]	0.02 [0.00 to 0.06]	Low
	Additional external (544 outcomes 90 treatment arms)	1.4 [1.2 to 1.6]					Moderate
**Large Effects (Achilles)**	Body weight (91 outcomes 14 treatment arms)	0.87 [0.43 to 1.2]	*p*(Body weight < Additional) = 0.979	0.85 [0.75 to 0.92]	0.10 [0.04 to 0.19]	0.05 [0.00 to 0.10]	Low
	Additional external (84 outcomes 21 treatment arms)	1.4 [1.1 to 1.8]					Low
**Large Effects (RCRSP)**	Body weight (60 outcomes 7 treatment arms)	0.60 [0.22 to 0.89]	*p*(Body weight < Additional) = 0.990	0.35 [0.22 to 0.50]	0.57 [0.44 to 0.71]	0.05 [0.01 to 0.11]	Low
	Additional external (216 outcomes 32 treatment arms)	1.0 [0.84 to 1.2]					Moderate
**Large Effects (Patellar)**	Body weight (10 outcomes 3 treatment arms)	0.76 [0.40 to 0.97]	*p*(Body weight < Additional) = 0.903	0.48 [0.21 to 0.68]	0.45 [0.26 to 0.72]	0.06 [0.00 to 0.14]	Very Low
	Additional external (93 outcomes 18 treatment arms)	1.1 [0.88 to 1.4]					Low
**Small Effects (All tendinopathies)**	Body weight (96 outcomes 11 treatment arms)	0.40 [0.21 to 0.53]	*p*(Body weight < Additional) = 0.619	0.70 [0.63 to 0.77]	0.27 [0.20 to 0.34]	0.02 [0.00 to 0.05]	Low

	Additional external (331 outcomes 49 treatment arms)	0.44 [0.33 to 0.55]					Moderate
**Small Effects (Achilles)**	Body weight (71 outcomes 6 treatment arms)	0.36 [-0.17 to 0.92]	*p*(Body weight < Additional) = 0.721	0.53 [0.34 to 0.71]	0.47 [0.29 to 0.65]	0.00 [0.00 to 0.02]	Very Low
	Additional external (51 outcomes 8 treatment arms)	0.56 [0.07 to 1.0]					Very Low
**Small Effects (RCRSP)**	Body weight (12 outcomes 2 treatment arms)	0.48 [0.13 to 0.84]	*p*(Body weight < Additional) = 0.520	0.76 [0.63 to 0.86]	0.22 [0.13 to 0.35]	0.00 [0.00 to 0.01]	Very Low
	Additional external (181 outcomes 21 treatment arms)	0.48 [0.36 to 0.60]					Moderate

Large Effects: Effect sizes obtained from outcomes measuring: 1) Disability; 2) Pain on loading/activity; 3) Pain without further specification; 4) Function; and 5) Pain over a specified time. Small Effects: Effect sizes obtained from outcomes measuring: 1) Quality of Life; 2) Physical Functional Capacity. CrI= credible interval. VPC= variance partition coefficient. RCRSP= rotator cuff related shoulder pain.

Supplementary table 9B. Moderator analysis comparing average pooled effect size for different training frequencies. Results presented across all tendinopathies and individual tendinopathies.

Moderator		Pooled SMD_{pre} estimate [95% CrI]	Probability	Study VPC [75% CrI]	Outcome VPC [75% CrI]	Measurement occasion VPC [75% CrI]	Confidence in evidence
**Large Effects (All tendinopathies)**	Less than daily (270 outcomes 45 treatment arms)	1.5 [1.3 to 1.7]	*p*(Less than daily > Once per day) = 0.992	0.77 [0.71 to 0.82]	0.19 [0.15 to 0.24]	0.04 [0.00 to 0.08]	Low
	Once per day (192 outcomes 33 treatment arms)	1.0 [0.69 to 1.3]	*p*(Once per day < More than once per day) = 0.693				Moderate
	More than once per day (305 outcomes 51 treatment arms)	1.2 [1.0 to 1.4]	*p*(Less than daily > More than once per day) = 0.951				Moderate
**Large Effects (Achilles)**	Less than daily 10 outcomes 3 treatment arms)	2.2 [1.2 to 3.4]	*p*(Less than daily > Once per day) = 0.989	0.85 [0.75 to 0.92]	0.10 [0.05 to 0.18]	0.04 [0.00 to 0.11]	Very low
	Once per day (40 outcomes 10 treatment arms)	1.0 [0.51 to 1.5]	*p*(Once per day < More than once per day) = 0.712				Low
	More than once per day (133 outcomes 24 treatment arms)	1.2 [0.86 to 1.5]	*p*(Less than daily > More than once per day) = 0.976				Moderate
**Large Effects (RCRSP)**	Less than daily (121 outcomes 18 treatment arms)	1.0 [0.72 to 1.4]	*p*(Less than daily > Once per day) = 0.815	0.42 [0.28 to 0.56]	0.49 [0.36 to 0.63]	0.09 [0.01 to 0.17]	Moderate
	Once per day (76 outcomes 11 treatment arms)	0.78 [0.42 to 1.1]	*p*(Once per day < More than once per day) = 0.986				Moderate
	More than once per day (104 outcomes 13 treatment arms)	1.4 [0.97 to 1.7]	*p*(Less than daily < More than once per day) > 0.896				Low
**Small Effects (All tendinopathies)**	Less than daily (174 outcomes 21 treatment arms)	0.60 [0.46 to 0.74]	*p*(Less than daily > Once per day) = 0.999	0.67 [0.58 to 0.74]	0.30 [0.23 to 0.39]	0.02 [0.00 to 0.06]	Moderate
	Once per day (156 outcomes	0.28 [0.10 to 0.45]	*p*(Once per day < More than once per day) = 0.802				Moderate

	20 treatment arms)						
	More than once per day (107 outcomes 19 treatment arms)	0.39 [0.22 to 0.53]	*p*(Less than daily > More than once per day) =0.976				Low
**Small Effects (Achilles)**	Less than daily (26 outcomes 2 treatment arms)	0.79 [0.20 to 1.4]	*p*(Less than daily > Once per day) = 0.997	0.28 [0.12 to 0.48]	0.71 [0.51 to 0.87]	0.00 [0.00 to 0.03]	Very low
	Once per day (51 outcomes 5 treatment arms)	0.28 [-0.07 to 0.63]	*p*(Once per day > More than once per day) = 0.533				Low
	More than once per day (46 outcomes 8 treatment arms)	0.28 [0.01 to 0.60]	*p*(Less than daily > More than once per day) = 0.998				Moderate
**Small Effects (RCRSP)**	Less than daily (105 outcomes 14 treatment arms)	0.53 [0.37 to 0.68]	*p*(Less than daily > Once per day) = 0.948	0.62 [0.47 to 0.75]	0.30 [0.18 to 0.43]	0.07 [0.00 to 0.17]	Moderate
	Once per day (46 outcomes 5 treatment arms)	0.27 [0.04 to 0.51]	*p*(Once per day > More than once per day) = 0.602				Very low
	More than once per day (44 outcomes 6 treatment arms)	0.29 [0.03 to 0.55]	*p*(Less than daily > More than once per day) = 0.902				Low

Large Effects: Effect sizes obtained from outcomes measuring: 1) Disability; 2) Pain on loading/activity; 3) Pain without further specification; 4) Function; and 5) Pain over a specified time. Small Effects: Effect sizes obtained from outcomes measuring: 1) Quality of Life; 2) Physical Functional Capacity. CrI= credible interval. VPC= variance partition coefficient. RCRSP= rotator cuff related shoulder pain.

Supplementary table 9C. Moderator analysis comparing average pooled effect size for binary resistance volume categorisation. Results presented across all tendinopathies and individual tendinopathies.

**Moderator**		**Pooled** SMD_{pre} **estimate [95% CrI]**	**Probability**	**Study VPC [75% CrI]**	**Outcome VPC [75% CrI]**	**Measurement occasion VPC [75% CrI]**	**Confidence in evidence**
**Large Effects (All tendinopathies)**	Lower Volume (377 outcomes 63 treatment arms)	1.2 [0.95 to 1.3]	*p*(Higher volume < Lower volume) = 0.553	0.80 [0. 74 to 0.85]	0.17 [0.13 to 0.21]	0.03 [0.00 to 0.07]	Moderate
	Higher Volume (355 outcomes 60 treatment arms)	1.2 [0.95 to 1.3]					Moderate
**Large Effects (Achilles)**	Lower Volume (29 outcomes 3 treatment arms)	1.1 [0.15 to 2.1]	*p*(Higher volume > Lower volume) = 0.601	0.85 [0.73 to 0.92]	0.10 [0.04 to 0.20]	0.04 [0.00 to 0.11]	Low
	Higher Volume (142 outcomes 32 treatment arms)	1.2 [0.93 to 1.6]					Moderate
**Large Effects (RCRSP)**	Lower Volume (220 outcomes 33 treatment arms)	0.97 [0.76 to 1.2]	*p*(Higher volume > Lower volume) = 0.989	0.44 [0.32 to 0.57]	0.45 [0.34 to 0.58]	0.10 [0.01 to 0.18]	Moderate
	Higher Volume (75 outcomes 7 treatment arms)	1.5 [1.1 to 1.9]					Moderate
**Large Effects (Patellar)**	Lower Volume (62 outcomes 11 treatment arms)	1.0 [0.62 to 1.4]	*p*(Higher volume > Lower volume) = 0.54	0.68 [0.46 to 0.82]	0.29 [0.14 to 0.49]	0.03 [0.00 to 0.09]	Low
	Higher Volume (47 outcomes 12 treatment arms)	1.0 [0.66 to 1.4]					Low
**Large Effects (Lateral elbow)**	Lower Volume (63 outcomes 13 treatment arms)	1.7 [0.70 to 2.5]	*p*(Higher volume < Lower volume) = 0.612	0.73 [0.57 to 0.84]	0.24 [0.14 to 0.39]	0.02 [0.00 to 0.04]	Low
	Higher Volume (91 outcomes 10 treatment arms)	1.6 [0.62 to 2.5]					Low
	Lower Volume	0.56 [0.37 to 0.74]		0.71 [0.63 to 0.78]	0.27 [0.20 to 0.35]	0.02 [0.00 to 0.05]	Moderate

**Small Effects (All tendinopathies)**	(224 outcomes 34 treatment arms)		*p*(Higher volume < Lower volume) = 0.782				
	Higher Volume (183 outcomes 25 treatment arms)	0.42 [0.26 to 0.59]					Moderate
**Small Effects (RCRSP)**	Lower Volume (165 outcomes 19 treatment arms)	0.47 [0.33 to 0.61]	*p*(Higher volume > Lower volume) = 0.792	0.75 [0.60 to 0.86]	0.23 [0.13 to 0.37]	0.01 [0.00 to 0.03]	Moderate
	Higher Volume (20 outcomes 3 treatment arms)	0.61 [0.28 to 0.96]					Low
**Small Effects (Lateral elbow)**	Lower Volume (18 outcomes 8 treatment arms)	0.45 [0.05 to 0.82]	*p*(Higher volume < Lower volume) = 0.633	0.53 [0.08 to 0.84]	0.46 [0.15 to 0.90]	0.00 [0.00 to 0.02]	Low
	Higher Volume (41 outcomes 6 treatment arms)	0.38 [0.02 to 0.78]					Low

Large Effects: Effect sizes obtained from outcomes measuring: 1) Disability; 2) Pain on loading/activity; 3) Pain without further specification; 4) Function; and 5) Pain over a specified time. Small Effects: Effect sizes obtained from outcomes measuring: 1) Quality of Life; 2) Physical Functional Capacity. CrI= credible interval. VPC= variance partition coefficient. RCRSP= rotator cuff related shoulder pain.
